# The burden of cough in idiopathic pulmonary fibrosis and other interstitial lung diseases: a systematic evidence synthesis

**DOI:** 10.1186/s12931-024-02897-w

**Published:** 2024-08-27

**Authors:** Rhiannon Green, Michael Baldwin, Nick Pooley, Kate Misso, Maureen PMH Rutten-van Mölken, Nina Patel, Marlies S. Wijsenbeek

**Affiliations:** 1 Market Access, Maverex Limited, Manchester, UK; 2 Value and Patient Access, Boehringer Ingelheim International GmbH, Ingelheim am Rhein, Germany; 3 Erasmus School of Health Policy and Management, Erasmus University Rotterdam, Rotterdam, The Netherlands; 4 Inflammation Medicine, Boehringer Ingelheim Pharmaceuticals Inc, Ridgefield, CT USA; 5 Respiratory Medicine, Pulmonary Medicine, Erasmus Medical Center, University Medical Center Rotterdam, ‘s-Gravendijkwal 230, Rotterdam, 3015 CE The Netherlands

**Keywords:** Chronic cough, Burden, Quality of life, Health-related quality of life, Idiopathic pulmonary fibrosis, Progressive pulmonary fibrosis, Interstitial lung disease, Connective tissue disease-associated interstitial lung disease, Sarcoidosis, Cough

## Abstract

**Background:**

Cough remains a persistent symptom in patients with idiopathic pulmonary fibrosis (IPF) and other interstitial lung diseases (ILDs). To inform future research, treatment and care models, we conducted the first systematic synthesis of evidence on its associated burden.

**Methods:**

A literature search was performed for articles published between January 2010 and October 2023 using databases including Embase, MEDLINE and the Cochrane Library. Studies in patients with IPF and other ILDs reporting cough-related measures were eligible for inclusion. Included studies were categorised based on the types of ILD they examined and their design. Study details, patient characteristics and outcomes were extracted, and the risk of bias was assessed. A narrative synthesis approach was employed to interpret the findings.

**Results:**

Sixty-one studies were included: 33 in IPF, 18 in mixed-ILDs, six in connective tissue disease-associated-ILDs and four in sarcoidosis. Across the studies, a range of tools to assess cough and its impact were used. The most frequently used measures of cough were cough severity visual analogue scale (VAS) and objective cough counts, whereas the most frequently used health-related quality of life (HRQoL)/impact measures were the St. George’s Respiratory Questionnaire (SGRQ) and Leicester Cough Questionnaire (LCQ). In IPF, studies consistently reported correlations between various cough and HRQoL measures, including between cough VAS scores and objective cough counts, LCQ scores and SGRQ scores. Similar correlations were observed in studies in other ILDs, but data were more limited. Qualitative studies in both IPF and other ILDs consistently highlighted the significant cough-related burden experienced by patients, including disruption of daily activities, fatigue and social embarrassment. Although there were no studies specifically investigating the economic burden of cough, one study in patients with fibrotic ILD found cough severity was associated with workplace productivity loss.

**Conclusions:**

Our study underscores the heterogeneity in assessing cough and its impact in IPF and other ILDs. The findings confirm the negative impact of cough on HRQoL in IPF and suggest a comparable impact in other ILDs. Our synthesis highlights the need for standardised assessment tools, along with dedicated studies, particularly in non-IPF ILDs and on the economic burden of cough.

**Supplementary Information:**

The online version contains supplementary material available at 10.1186/s12931-024-02897-w.

## Introduction

Interstitial lung disease (ILD) encompasses a heterogenous group of respiratory conditions characterised by inflammation and/or fibrosis of the lung parenchyma [1, 2]. Idiopathic pulmonary fibrosis (IPF), the most common and well-studied type of ILD, is associated with progressive lung function decline and poor prognosis [3]. Several other types of ILD, including hypersensitivity pneumonitis (HP), sarcoidosis and connective tissue disease-associated ILD (CTD-ILD) such as systemic sclerosis associated-ILD (SSc-ILD) and rheumatoid arthritis-associated ILD (RA-ILD), carry a risk of developing a similar progressive phenotype, referred to as ‘progressive pulmonary fibrosis’ or ‘progressive-fibrosing ILD’ (PPF/PF-ILD) [2, 4].

Among patients with ILD, cough is a prevalent symptom and can sometimes manifest as the initial sign of the disease [5–7]. Cough tends to persist over time in ILD populations, with chronic cough reported in 50─90% of patients with IPF [8, 9]. The pathophysiology of cough in ILD is considered multifactorial, involving mechanisms such as mechanical distortion, heightened cough sensitivity reflex, increased mucus production, the presence of inflammatory mediators and the influence of comorbidities such as gastroesophageal reflux disease, asthma, non-asthmatic eosinophilic bronchitis and obstructive sleep apnoea [6, 10–14]. It has been suggested that cough may contribute to a profibrotic feedback loop that drives disease progression in ILD [6, 9]. While there is some evidence that cough is an independent predictor of prognosis in IPF [15], findings have been mixed [16].

Although advances have been made in the treatment of IPF and other ILDs [17, 18], the lack of specific treatments for cough remains a significant concern for patients, with up to one-third of patients with ILD ranking cough as their worst symptom [7]. To inform future treatment and care models, it is vital to understand the breadth and magnitude of the burden of cough in these populations.

While the wide-ranging and multifaceted impact of cough has been explored in various populations [19–22], there has been no previous systematic synthesis of literature focussed on the impact in IPF and other ILDs. The objective of this review is therefore to bridge this knowledge gap by providing the first systematic evidence synthesis of the full spectrum of humanistic and economic impact related to cough in IPF and other ILDs. Through this synthesis, we aim to shed light on the multidimensional impact of cough, identify any gaps in existing literature, discuss considerations for future research and ultimately help pave the way for the development of effective treatment and support strategies for cough in IPF/ILD.

## Methods

### Search strategy

The protocol for this systematic review was prospectively registered with PROSPERO (CRD42022369379). Search strategies were developed following the guidance provided by the Centre for Reviews and Dissemination (CRD) and the Cochrane Handbook. Electronic databases, including Embase (OVID), MEDLINE (OVID), PubMed, Europe PMC, Cochrane Database of Systematic Reviews (CDSR), Cochrane Central Register of Controlled Trials (CENTRAL) and NHS Economic Evaluation Database (NHS EED), were initially searched on 31 August 2022, to identify studies on the burden of cough in IPF and other ILDs. Update searches were conducted on 3–4 October 2023.

Searches included combinations of relevant indexing and free-text terms. Terms were adapted to meet the syntax requirements of each database, and Boolean operators were employed to combine concepts effectively. Searches were limited to articles published between January 2010 and October 2023. The full search strategy, including the search terms and syntax used for each database, is provided in the Supplementary Methods within Additional file 1.

Database searches were supplemented with hand searching and berry-picking techniques, including free-text searches using Google Scholar, and a review of the reference lists of included studies.

### Study selection

Studies in patients with IPF and other ILDs reporting cough characteristics and relevant humanistic or economic outcomes were eligible for inclusion. Non-human studies, case studies (based on study design), letters, editorials, commentaries and studies that did not report relevant outcomes were excluded. Two independent reviewers used Rayyan software to screen the retrieved articles based on the predefined inclusion and exclusion criteria shown in Table 1. Initially, titles and abstracts were reviewed within the software to assess their eligibility for inclusion. Subsequently, the full texts of potentially eligible articles were assessed for final inclusion. Discrepancies between reviewers were resolved through discussion with a third reviewer until consensus was reached. For articles reviewed in full, reasons for exclusion were recorded.

**Table 1:** Inclusion/exclusion criteria

Inclusion criteria
Studies in patients with IPF and other ILDs: ILDs including idiopathic conditions (IPF and non-IPF idiopathic interstitial pneumonias), autoimmune-related ILDs, exposure related conditions, chronic sarcoidosis, hypersensitivity pneumonitis and coal workers’ pneumoconiosis
Studies reporting relevant cough-related outcomes: Humanistic burden of cough – patient reported through use of health-related quality of life instruments or qualitative assessment (focus group/interview series), caregiver health, and caregiver quality of life Economic burden of cough – healthcare resource use, direct and indirect costs, productivity losses
RCTs with relevant cough-related baseline measurements
Observational studies including cohort and population-based studies reporting relevant cough-related measures

Exclusion criteria
Patients without interstitial lung disease or without cough symptoms
In vitro studies
Studies that do not report relevant outcomes
Studies published prior to 2010
Non-English full-text articles
Non-human studies
Not containing original data
Case reports and case series
Conference abstracts
Editorials, commentaries, and letters

ILD: interstitial lung disease; IPF: idiopathic pulmonary fibrosis; RCT: randomised controlled trial

### Data extraction and synthesis

For included articles, data extraction was performed by a primary reviewer using a standardised data-extraction template. The extracted data included article details, study design, participant demographics, cough characteristics, intervention details, control/comparison groups, outcome measures and relevant findings. A narrative synthesis approach was employed to summarise and interpret the findings from the included studies. This involved the use of text and tables to provide a comprehensive summary of the data and identify key trends.

Included studies were stratified by the types of ILD they examined and their design. Additionally, to allow for an analysis of heterogeneity and comparison of outcomes across different study designs and populations, studies were also grouped by whether chronic cough was specified, the prevalence of cough and whether cough was the major focus of the study:

- Chronic cough: Studies in patients with chronic cough (definition not standard across studies).
- Majority cough population: Studies where the majority of patients reported cough (not further specified).
- Minority cough population: Studies where < 50% of patients reported cough (not further specified) or the prevalence of cough was not reported.
- Broader includes: Studies where the prevalence of cough was not reported and cough was not the focus, but cough outcome measures were collected.

The risk of bias was assessed by two independent reviewers using design-specific appraisal tools. These included the appraisal tool for cross-sectional studies (AXIS) [23], the Cochrane risk-of-bias tool 2 for randomised controlled trials (RCTs) [24], the Critical Appraisal Skills Programme for qualitative studies [25] and the consensus-based standards for the selection of health measurement instruments for studies validating patient-reported outcomes [26].

## Results

**Fig. 1:**
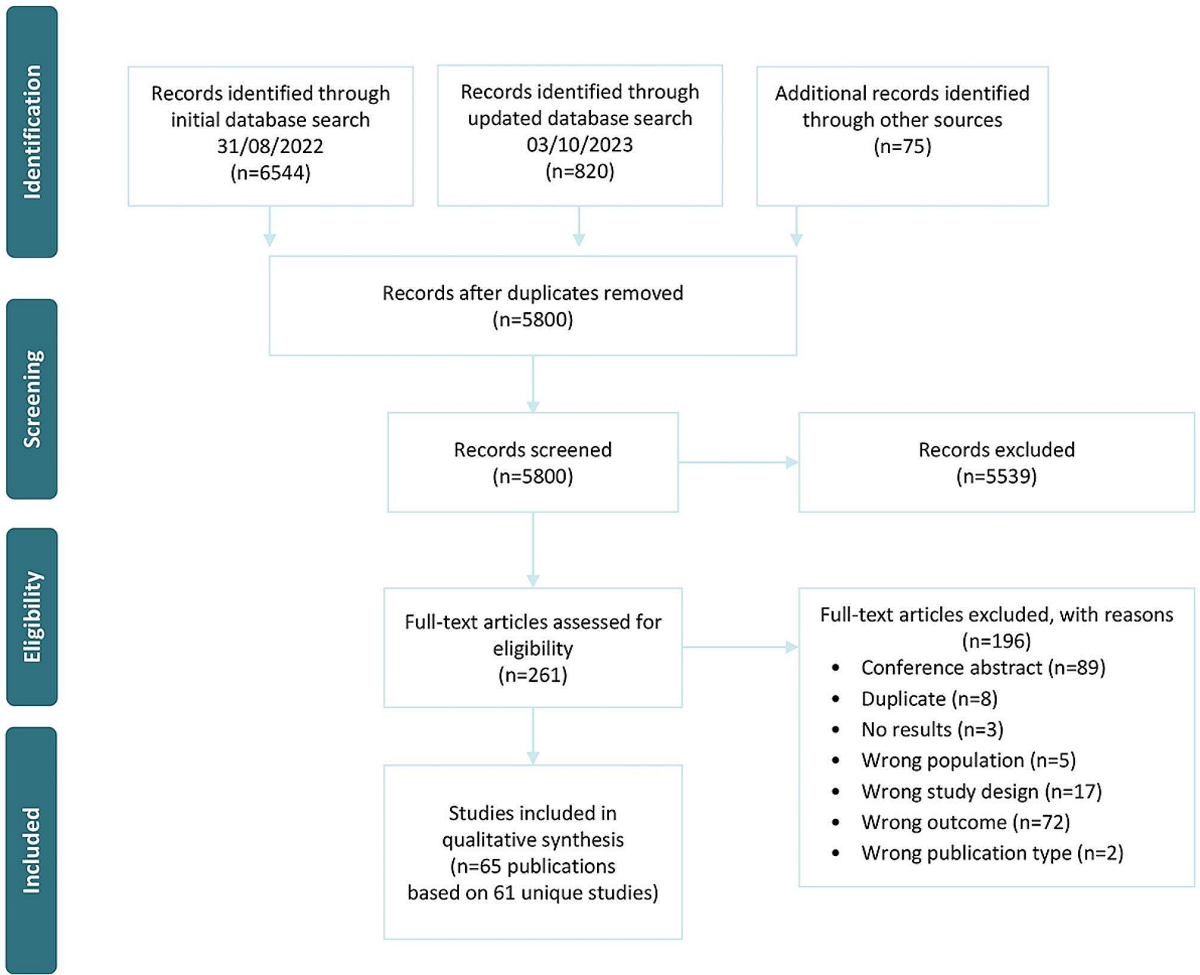
Flow of articles through the different phases of the systematic synthesis

The searches yielded 7,439 articles, with 7,364 identified through electronic database searches and 75 through free-text and hand-searching. After screening, 261 unique articles remained and were subject to full-text assessment, with 65 eligible articles, based on 61 unique studies, ultimately included in the synthesis. The study selection process is illustrated in Fig. 1.

**Fig. 2:**
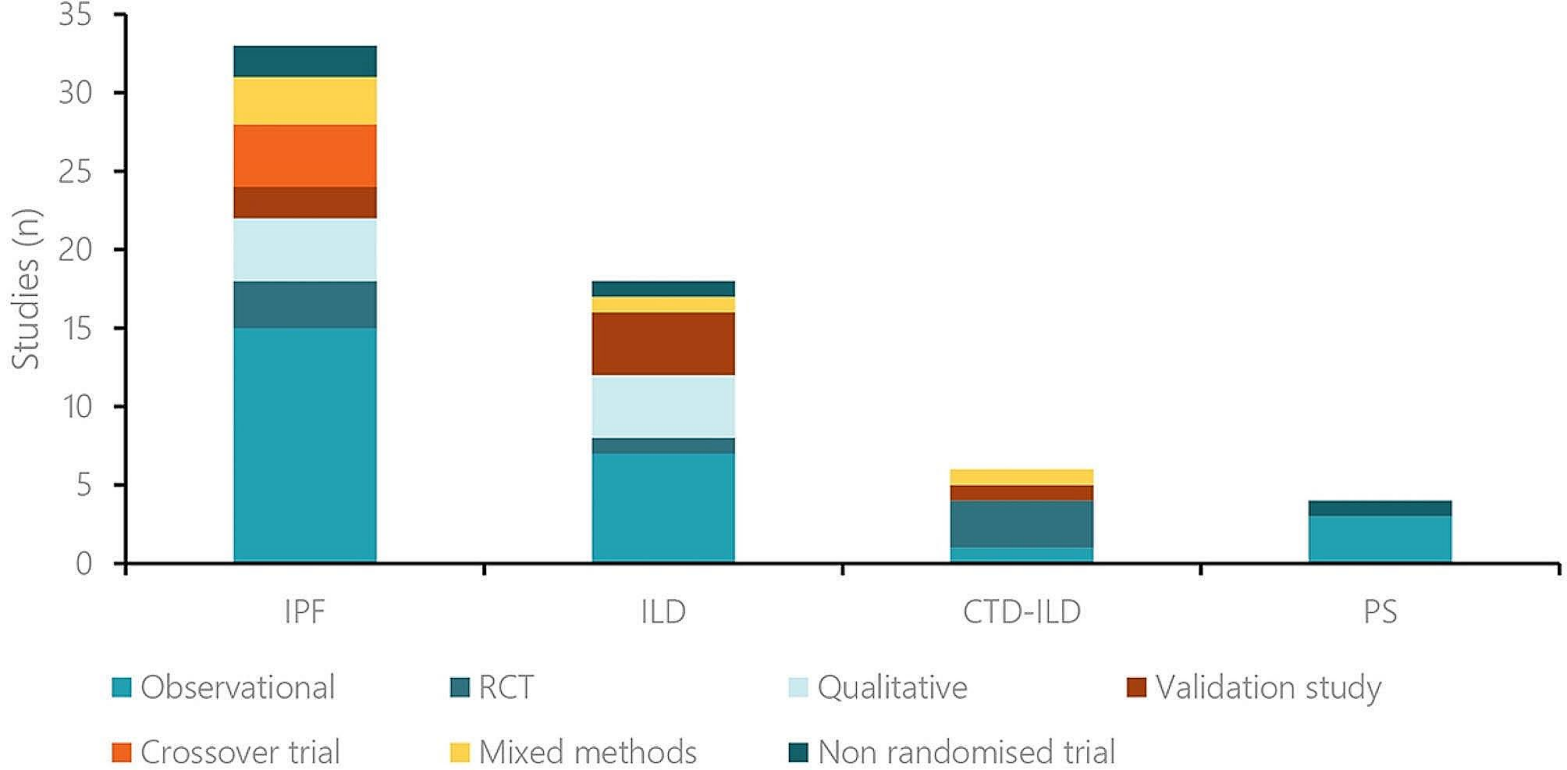
Overview of included studies by disease group and study design* *Totals were calculated based on the number of unique studies rather than articles. For IPF, two articles were based on the same trial (NCT00600028), counted as a single trial and two articles were derived from the same cohort/registry study (PROOF), counted as one observational study. Similarly, for CTD-ILD, three articles were based on the same trial, counted as a single trial (NCT00883129). CTD-ILD, connective tissue disease-associated ILD; ILD, interstitial lung disease; IPF, idiopathic pulmonary fibrosis; PS, pulmonary sarcoidosis; RCT, randomised controlled trial

Among the unique studies, there were 33 focussed on patients with IPF, 18 on patients with mixed ILDs (various types of ILD, including CTD-ILDs in some cases), six on patients with CTD-ILD, and four on patients with sarcoidosis. The number of unique studies by disease group and study design is illustrated in Fig. 2. For each disease group, a comprehensive overview of the study designs, patient characteristics and outcomes for the included studies is available in the Supplementary Results within Additional file 2. Key findings are summarised in the subsections below.

Based on design-specific appraisal tools, 29 studies were rated as having a low risk of bias and 28 as having a medium risk. Four studies were not assessed for bias due to the lack of suitable tools to assess their design. An overview of the bias assessment for each study is provided in the Supplementary Results within Additional file 2.

While treatment effects are not the focus of this review and we do not discuss impact of treatments on cough outcomes, a proportion of the studies were investigating pharmacological interventions including antibiotics, antifibrotics, corticosteroids, opioids, anti-acid medication, immunosuppressants, neuromodulators, sodium cromoglycate and thalidomide.

### Studies in IPF

#### IPF: study characteristics and measures

Among the 33 studies in patients with IPF, there were nine interventional trials [27–35], 15 observational studies [8, 36–49], two validation studies [50, 51], three mixed-methods studies [52–54] and four qualitative studies [55–58]. An overview of the quantitative and mixed-methods studies is provided in Table 2.

**Table 2:** Quantitative and mixed-methods studies in IPF

Author year	Cough category	Study design	Arm	FVC % pred.	BL cough severity measures	BL HRQoL/impact measures	Group comparisons	Findings related to the burden of cough
**Interventional trials**								
Birring 2017	Chronic cough	Crossover RCT	IPF: 24	73 (15)	LCM 24-hr cough frequency: NR	LCQ: 12.9 (3.2)	LCQ scores were lower in CIC patients, and the response of IPF to treatment suggests the mechanism of cough may be disease-specific	Significant correlation between daytime cough frequency, VAS cough severity (r = 0.683) and LCQ (r=–0.682)
					VAS: 61.5 (13.0)	KBILD: 56.2 (10.5)		
			CIC: 27	–	LCM 24-hr cough frequency: NR	LCQ: 10.5 (15.3)		
					VAS: 70.5 (15.3)	KBILD: NA		
Dutta 2019	Chronic cough	Pilot trial	Omeprazole: 23	73.1 (17.1)	24-hr cough monitor: 9.6/hr (4.2–18.3)	LCQ: 15.3 (3.3)	There was a greater reduction in 24-hr cough frequency in the omeprazole group compared with placebo	Change in objective cough not reflected in subjective measures
						Physical: 5.1 (1.1)		
						Physiological: 5.0 (1.4)		
						Social: 5.2 (1.1)		
			Placebo: 22	77.9 (17.6)	24-hr cough monitor: 8.9/hr (6.8–12.8)	LCQ: 15.1 (3.2)		
						Physical: 5.2 (1)		
						Physiological: 4.8 (1.2)		
						Social: 5.1 (1.3)		
Lechtzin 2013/ Horton 2012	Chronic cough	Crossover RCT	23	70.4 (13.7)	VAS: 64.8 (21.4)	CQLQ: 60.5 (12)	–	Cough VAS correlated with SGRQ and physical and functional CQLQ; cough aetiology discussed
						SGRQ: 57.4 (18.8)		
Martinez 2021	Chronic cough	Crossover RCT	Gefapixant: 40	All patients: 51. Ratio: 83.6 (10.60)	LCM mean cough count: 46.2 (43.06)	CQLQ: 56.5 (13.26)	–	Reductions in cough severity measures were not reflected in cough-related QoL
					VAS: 56.0 (24.03) (https://classic.clinicaltrials.gov/ct2/show/results/NCT02502097)	Cough Severity Diary: 4.5 (1.75)		
			Placebo: 40		LCM mean cough count: 48 (55.17)	CQLQ: 56.8 (11.25)		
					VAS: 53.9 (22.8)	Cough Severity Diary: 4.1 (2.04)		
Martinez 2022	Chronic cough	RCT	RVT-1601 10 mg: 29	66.4 (16.8)	24-hr cough count: 38.6./hr (23.3)	LCQ: 11.4 (3.90)	–	There were no significant differences in LCQ or cough counts between treatment groups
					VAS: 71.9 (13.3)			
			RVT-1601 40 mg: 25	68.0 (14.2)	24-hr cough count: 38.7/hr (22.0)	LCQ: 11.1 (3.54)		
					VAS: 73.7 (15.67)			
			RVT-1601 80 mg: 27	69.2 (17.5)	24-hr cough count: 37.1/hr (19.5)	LCQ: 11.0 (3.53)		
					VAS: 69.8 (12.34)			
			Placebo: 27	71.7 (18.7)	24-hr cough count: 36.6/hr (20.3)	LCQ: 11.6 (3.40)		
					VAS: 69.2 (16.29)			
Lee 2023	Chronic cough	Single-arm trial	30	77.50 (67.75–92.50)	–	LCQ: 16.77 (15.63–19.13)	33.3% of patients achieved MCID after treatment (median difference of total LCQ score: 2.38, range: 1.79–3.32)	There was no significant improvement in cough-related QoL (median difference 0.12, range: − 0.73–0.97, P = 0.772) or respiratory-related QoL measurements
						Physical: 5.44 (4.88–6)		
						Psychological: 5.57 (4.96–6.61)		
						Social: 6 (5.5–6.75)		
						SGRQ: 30.59 (19.41–37.82)		
						Symptoms: 46.44 (35.08–55.96)		
						Activity: 45.1 (28.52–61.34)		
						Impact: 13.55 (6.08–23.78)		
Guler 2021	Chronic cough	Crossover RCT	20	66 (17)	VAS: 5.6 (2.3)*	SGRQ: 57.2 (18.6)	–	LCQ correlated negatively with VAS severity (r=–0.42) and VAS correlated moderately with SGRQ (r = 0.42)
					Cough index /h (n = 15)	LCQ: 11.7 (3.7)		
					Wake: 6.2 (4.4–8.2)	Physical: 4.3 (1.1)		
					Sleep: 0.6 (0.1–1.1)	Physiological: 3.6 (1.4)		
					Cough Attack Index /h (n = 15)	Social: 3.8 (1.5)		
					Wake: 0.9 (0.7–1.3)			
					Sleep: 0.1 (0–0.4)			
Wilson 2021	Broader includes	RCT	341	56.2 (8.9)	VAS: 44.7 (27.0)	LCQ: 16.08 (3.55)	–	Cough correlation with QoL not reported, after 18 months the co-trimoxazole group had better VAS cough but not LCQ or KBILD
						KBILD: 53.7 (9.71)		
Jastrzębski 2023	Broader includes	Single arm trial	87	82.02 (17.65)	–	LCQ: 14.47 (3.74)	–	After 12 months of pirfenidone treatment, 12% of patients experienced improvements in their QoL and cough and dyspnoea reduction but better mean LCQ was not statistically significant (14.47 ± 3.74 vs. 15.24 ± 3.61; P = 0.26).
						SGRQ: 46.69 (20.62)		
						Symptoms: 52.04 (21.24)		
						Activity: 55.63 (23.31)		
						Impact: 39.54 (23.95)		
						SF-36 PCS: 42.33 (8.73)		
						SF-36 MCS: 44.60 (11.85)		
**Observational studies**								
Saari 2023	Chronic cough	Case-control study	IPF with chronic cough: 46	78.50 (73.7–82.3)	Cough response to paint or fumes: 5.5 (5–7)	Cough-disturbing sleep: 5.5 (4–6)	IPF patients with chronic cough had significantly lower LCQ scores than those without. Scores for cough disturbing sleep and cough bout frequency per day were also lower. There were no significant differences between the IPF chronic cough and community-based chronic cough groups in LCQ total scores and the LCQ physical, psychological, and social impact scores or individual LCQ question scores	The prevalence of chronic cough was 68% among patients with IPF. The results suggested that in early-stage IPF, disease cough is undistinguishable from a community-based chronic cough
						LCQ: 14.8 (11.5–18.1)		
					Cough bout frequency per day: 4 (3–6)	Physical: 4.9 (3.9–6.1)		
						Psychological: 4.6 (3.7–5.9)		
						Social: 5.5 (3.7–6.5)		
			IPF without chronic cough: 22	84.5 (77.0–91.25)	Cough response to paint or fumes: 6 (6–7)	Cough-disturbing sleep: 7 (6–7)		
						LCQ: 18.2 (16.4–19.4)		
						Physical: 5.9 (5.1–6.4)		
					Cough bout frequency per day: 6 (5.75–7)	Psychological: 6 (5–6.7)		
						Social: 6.4 (5.8–6.8)		
			Chronic cough: 184	NR	Cough response to paint or fumes: 6 (4–7)	Cough-disturbing sleep: 6 (4–6)		
						LCQ: 15.4 (13–17.5)		
						Physical: 5.1 (4.5–5.6)		
					Cough bout frequency per day: 5 (4–5)	Psychological: 4.7 (3.9–5.7)		
						Social: 5.5 (4.5–6.3)		
Glaspole 2017	Majority cough	Cohort study	516	81.0 (22.5)	VAS: 40.3 (21–56)	SGRQ: 46.6 (20.9)	–	Cough was an independent predictor of QoL
						HADS-A: 4 (2–7)		
						HADS-D: 4 (2–7)		
Jones 2011	Majority cough	Cross-sectional study	IPF: 27	80.4 (20.9)	VAS: 38 (15–60)	LCQ: 15.9 (11.9–19.5)	IPF patients had significantly higher median cough symptom scores than healthy controls	Subjective cough measures showed strong correlation
					CSS: 4 (2–6)			
			Healthy control: 30	120.6 (13.7)	VAS: 0 (0–4.5)	LCQ: 20.8 (20.5–21)		
					CSS: 0 (0–0)			
Wuyts 2018/ Wuyts 2022	Majority cough	Cohort study	277	80.6 (19.9)	VAS: 30.5 (25.2)	SGRQ: 47.0 (20.2)	–	SGRQ total scores and SGRQ impact scores remained stable over time, but cough VAS, SGRQ activity scores and SGRQ symptom scores increased in all patients. Cough was not significantly associated with mortality
					Median (IQR): 24.0 (9.0–50.0)	EQ-5D VAS: 61.1 (19.2)		
					Minimum–maximum: 0.0–100.0	Current health perception poor/very poor: 15.9%		
Key 2010	Majority cough	Cohort study	19	78.5 (24.4)	Median 24-hr cough rate 9.4/h (1.5–39.4)	LCQ: 15.4 (6.95–20.88)	In *post hoc* analyses, cough rates in IPF were higher than healthy volunteers and patients with asthma and similar to patients with chronic cough	Strong correlations between objective cough frequency and cough VAS and cough-related QoL
					VAS: 32 (2–77)	Physical: 5.13 (2.38–6.63)		
						Psychological: 5.29 (1.57–7)		
						Social: 5.75 (2.25–7)		
Yount 2016	Majority cough	Cross-sectional study	220	–	–	ATAQ-IPF cough scale: 23.6 (5.8)	–	Cough was significantly associated with worse QoL
						FACIT: 2.5 (1.2)		
Tzouvelekis 2020	Minority cough	Cohort study	101	77.0 (21.2)	–	LCQ: 107.6 (30.2)**	–	Depression severity had a significant association with cough
						BDI-II: 13.7 (8.4)		
						KBILD: 69.3 (18.7)		
						SGRQ: 39.7 (23.7)		
Scholand 2014	Minority cough	Genetic study	68	72.57 (20.43)	–	LCQ: 16.16 (3.66)	–	Results suggested a genetic component to cough burden in IPF
Saunders 2023	Minority cough	Prospective cohort	632	74.1 (17.6)	–	LCQ: 16.1 (IQR 6.5)	There was no significant difference in survival between those with mild (LCQ > 14), moderate (LCQ > 10–<14) or worst cough (LCQ < 10). Patients with progressive disease experienced worsening cough-related QoL with a 12-month change in LCQ score of -2.2 (± 5.0) for the comparison between stable and progressive groups (P < 0.001)	Greater cough burden was not associated with worse survival when corrected for age, gender, BL lung function, and smoking history (HR 1.01; 95% CI = 0.97–1.03, P = 0.34). Longitudinal assessment of LCQ data suggested that the impact of cough-related QoL changes little over time for the majority of patients with IPF
						Physical: 5.1 (IQR 1.7)		
						Psychological: 5 (IQR 2.4)		
						Social: 5 (IQR 2.5)		
Prasad 2021	Broader includes	Cohort study	54	69.9 (16.7)	–	LCQ: 14.46 (0.71)	–	LCQ not associated with physical activity decline
						Physical: 4.77 (0.21)		
						Psychological: 4.83 (0.25)		
						Social: 4.86 (0.25)		
						SGRQ: 47.2 (2.84)		
						HADS-A: 6.11 (0.62), HADS-D: 5.74 (0.59)		
Park 2022	Broader includes	Cohort study	Without airway disease: 64	77.47 (1.9)	VAS: 3.31 (0.36)*	CQLQ: 47 (1.66)	No significant difference in baseline CAT, CQLQ and SGRQ scores between groups	Respiratory and cough-related QoL declined more in patients with IPF and airway disease than IPF alone
						SGRQ: 29.09 (2.65)		
						CAT: 14.78 (1.14)		
						EQ-5D index: 0.85 (0.071)		
			With airway disease: 6	75 (5.03)	VAS: 4.33 (1.2)*	CQLQ: 49.83 (8.6)		
						SGRQ: 33.16 (9.05)		
						CAT: 13.5 (13.9)		
						EQ-5D index: 0.744 (0.064)		
de Andrade 2021	Broader includes	Cohort study	Implementation score ≤ 0.6: 360	71.9 (62.7–82.1)	–	**CASA-Q**	There were no associations between the implementation score and patient-reported outcomes except a trend in SGRQ impact domain score	Cough burden not associated with guideline implementation; correlation with HRQoL was not presented
						Impact: 81.3 (59.4–96.9)		
						Symptom: 58.3 (41.7–83.3)		
						SGRQ: 33.2 (19.4–47.8)		
						EQ-5D index: 0.8 (0.7–1.0)		
						EQ-5D VAS: 80 (70–90)		
						**SF-36**		
						Physical: 41.8 (34–50)		
						Mental: 54.3 (45.9–59.7)		
			Implementation score > 0.6: 367	68.2 (57.2–79.9)	–	**CASA-Q**		
						Impact: 81.3 (59.4–96.9)		
						Symptom: 62.5 (41.7–75.0)		
						SGRQ: 40.6 (29.4–53.3)		
						EQ-5D index: 0.8 (0.7–1.0)		
						EQ-5D VAS: 75 (60–85)		
						**SF-36**		
						Physical: 37.9 (32.1–44.4)		
						Mental: 54.1 (46–59.9)		
Case 2020	Broader includes	Cohort study	662	NR	–	**CASA-Q**	–	CASA-Q was not associated with death or lung transplant after adjustment
						Symptoms: 58.3 (41.7–75)		
						Impact: 78.1 (56.3–93.8)		
						SGRQ: 39.5 (25.8–52.9)		
						**SF-36**		
						Physical: 39.2 (31.4–46.6)		
						Mental: 54.1 (46.5–58.8)		
						EQ-5D index: 0.8 (0.7–1.0)		
Kim 2022	Broader includes	Cohort study	CPFE: 119	71.8 (63.4–90.8)	–	**CASA-Q**	The effect of cough on HRQoL was significantly lower in CPFE than IPF as measured by CASA-Q. The only other significant difference was SGRQ activity domain, with worse scores in CPFE	The reasons for the lower impact of cough in patients with CPFE are unclear, but the more frequent use of inhaler therapy and systemic corticosteroids may be partly responsible
						Symptoms 66.7 (50.0–83.3)		
						Impact: 87.5 (68.8–96.9)		
						SGRQ: 41.8 (27.3–53.7)		
						EQ-5D index: 0.8 (0.7–0.9)		
						EQ-5D VAS: 70 (60–85)		
						**SF-12**		
						Physical: 37.5 (32.2–43.6)		
						Mental: 53.3 (45.1–60.2)		
			IPF: 815	69.4 (58.8–79.0)	–	**CASA-Q**		
						Symptoms: 58.3 (41.7–75)		
						Impact: 75 (56.3–93.8)		
						SGRQ: 39.5 (25.1–53.7)		
						EQ-5D index: 0.8 (0.7–1.0)		
						EQ-5D VAS: 75 (61.0–85)		
						**SF-12**		
						Physical: 39.4 (31.1–46.6)		
						Mental: 54.1 (45.8–59.2)		
Hollmen 2023	Broader includes	Cross-sectional, online survey	111	NR	–	I-Prefer questionnaire	Cough was more likely to prevent men, those on pirfenidone (vs nintedanib) and patients with severe IPF from doings things (P < 0.001)	Around 30% of the patients restricted their time outside to 1–3 hours; 58% restricted outdoor time to under 1 hour. Coughing prevented around 60% of the patients from their daily activities (shortness of breath prevented around 80%)
**Validation studies**								
Swigris 2010	Broader includes	Validation study	95	NR	–	ATAQ-IPF scores: 210 (46)	–	Cough domain included in the QoL tool
						ATAQ-IPF cough: 16 (7)		
Swigris 2018	Broader includes	Validation study using pooled RCT data	1061	79.6 (17.8)	–	**CASA-Q**	–	CASA-Q had moderate correlation with SGRQ domains
						Symptoms: 59.9 (23.2)		
						Impact: 75.2 (23)		
						SGRQ: 39.5 (18.9)		
**Mixed-methods studies**								
Igai 2022	Broader includes	Mixed methods	12	–	–	CAT cough: 1.75 (0.83)	–	There was a significant difference in the SGRQ-I symptom domain but not in the other domains or the CAT cough score after the intervention
						SGRQ-I: 54.19 (17.97)		
						HADS-A: 13.25 (2.05)		
						HADS-D: 14.25 (3.82)		
Bacci 2018	Majority cough	Cross-sectional qualitative study	Phase 1: 30	59.3 (9.5)	83% patients reported cough	–	–	95% of patients endorsed cough as a symptom
			Phase 2: 168	70.2 (13.21)	Urge to cough: 1.7 (0.8)	**E-RS: IPF Domain Scores**		The findings indicated that RS-Breathlessness and RS-Chest were most sensitive to disease severity, then RS-Cough and RS-Sputum
						Breathlessness: 6.3 (3.6)		
					Discomfort due to cough: 1.2 (1.0)	Cough 1.7 (0.8)		
						Chest 3.0 (2.1)		
					Intensity of cough: 1.2 (0.9)	Sputum 2.1 (1.5)		
Gries 2013	Majority cough	Mixed methods	18	87.2 (30.7)	–	**CASA-Q**	Burden slightly worse than in COPD or chronic bronchitis	All the cough items in CASA-Q were generally perceived as highly relevant
						Symptom: 46.8 (19.2)		
						Impact: 57.1 (22.3)		
						**Self-reported overall health**:		
						Very good/Good: 10 (55.5%)		
						Fair/Poor: 8 (44.5%)		

Mean (SD) or Median (range or IQR)

*Reported on alternative 0–10 cm scale

** Reported on alternative 100-point scale

ATAQ-IPF, A Tool to Assess Quality of Life in Idiopathic Pulmonary Fibrosis; BL, baseline; CAT, COPD Assessment Test; CI, confidence interval; CIC, chronic idiopathic cough; COPD, chronic obstructive pulmonary disease; CPFE, combined pulmonary fibrosis and emphysema; CQLQ, Cough Quality of Life Questionnaire; CSS, cough severity score; EQ-5D, EuroQol EQ-5D; FACIT, Functional Assessment of Chronic Illness Therapy; FVC, forced vital capacity; HADS-A, Hospital Anxiety and Depression Scale Anxiety score; HADS-D, Hospital Anxiety and Depression Scale Depression score; HR, hazard ratio; HRQoL, health-related quality of life; ILD, interstitial lung disease; IPF, idiopathic pulmonary fibrosis; IQR, interquartile range; KBILD, King’s Brief Interstitial Lung Disease; LCM, Leicester Cough Monitor; LCQ, Leicester Cough Questionnaire; MCID, minimal clinically important difference; MCS, Mental Component score; NA, not applicable; NR, not reported; Physical Component score; QoL, quality of life; RCT, randomised controlled trial; SF-36, Short Form 36; SGRQ, St. George’s Respiratory Questionnaire; SGRQ-I, St. George’s Respiratory Questionnaire version for Idiopathic Pulmonary Fibrosis; VAS, visual analogue scale

Eight of the studies were classed as chronic cough studies, comprising seven trials [27–33] and one observational study [36]. These studies used various definitions for chronic cough, including history of cough (with or without exertional dyspnoea) [28], self-reported chronic cough [30] and cough for > 8 weeks [27, 29, 30, 59]. Some of the trials required further criteria, such as stable cough frequency for > 4 weeks [30], cough affecting daily life or quality of life [29, 33, 59], refractory cough [27, 33], 24-hour cough count of > 10/15 coughs per/hour [27, 31], and/or visual analogue scale (VAS) for cough severity > 40 mm [27, 30, 31].

Across the studies in IPF, a wide range of cough, health-related quality of life (HRQoL) and other impact measures were used (Table 2). The most frequently used cough measures were cough severity VAS (N studies = 11 [27, 30, 31, 33, 34, 37–40, 45, 59]; N patients = 1543; reported as mean score range 30.5–73.7, reported as median score range 32–40.3; possible score range 0–100 [no cough–worst cough]) and objective cough counts (N studies = 5 [27, 28, 30, 31, 40]; N patients = 186; mean count/hr range 8.9–48.0). Apart from one observational study in a majority cough population [40], the only studies that used objective cough counts were interventional trials in patients with chronic cough. Outside of interventional trials, cough was often assessed using cough items or domain scores from other HRQoL or impact measures. The St. George’s Respiratory Questionnaire (SGRQ) was the most frequently used HRQoL measure (N studies = 14 [32, 33, 35, 37, 39, 42, 44–48, 51, 52, 59], N patients = 3988; mean score range 29.09–57.4; possible score range 0–100 [lower–greater impact on HRQoL]), followed by the Leicester Cough Questionnaire (LCQ; N studies = 14 [8, 27, 28, 31–36, 38, 40, 42–44], N patients = 1532; reported as mean score range 11.0–16.16, reported as median score range 14.8–18.2; possible score range 3.0–21.0 [lower scores indicate worse cough specific quality of life]).

#### IPF: comparisons with other populations

**Fig. 3:**
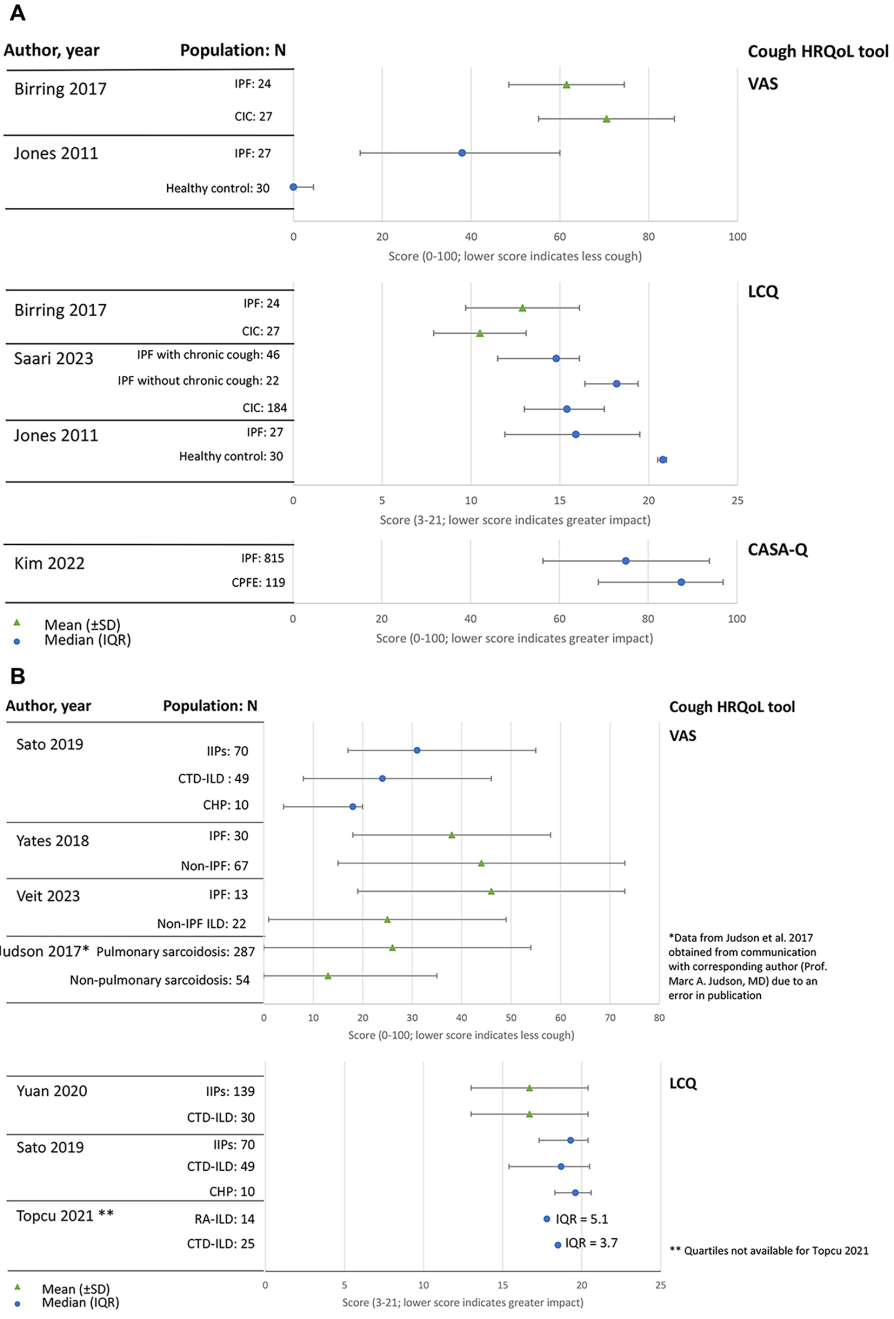
Overview of studies assessing cough severity and/or HRQoL measures in (**a**) IPF and (**b**) ILD in comparison with other populations

Six studies compared cough severity, HRQoL and/or other impact measures in patients with IPF with other populations ( [27, 36, 38, 40, 48, 54], Table 2; Fig. 3). A trial investigating the use of PA101 (sodium cromoglicate) found that patients with IPF and chronic cough had a higher baseline objective cough frequency than patients with chronic idiopathic cough (CIC), but better VAS and LCQ scores [27]. PA101 reduced objective daytime cough counts in patients with IPF but not in patients with CIC, indicating a potentially different mechanism of cough [27]. However, an observational study comparing patients with IPF and chronic cough and patients with CIC found no between-group differences in LCQ scores or other features of cough, including cough responses to paint or fumes, cough-related sleep disturbance or self-reported cough frequency [36]. An observational study in a majority-cough population similarly found that objective cough rates in patients with IPF were comparable with other patients with chronic cough and higher than previously published rates in healthy controls and patients with asthma [40]. Based on worse VAS, LCQ and/or Cough and Sputum Assessment Questionnaire (CASA-Q) scores, other studies found that patients with IPF had more severe cough compared with healthy volunteers, patients with combined pulmonary fibrosis and emphysema, and previously published values in patients with COPD and chronic bronchitis [38, 48, 54].

#### IPF: association of cough with impact/HRQoL and economic measures

Nine studies investigated concurrent/baseline associations between cough, HRQoL and/or other impact measures in patients with IPF, with all of these reporting at least one association ( [27, 33, 37, 38, 40–42, 53, 59], Table 2). These included two interventional trials in patients with IPF and chronic cough where cough severity VAS was correlated with Cough Quality of Life Questionnaire (CQLQ) (N patients = 23, *R* = 0.63), LCQ (N patients = 20, R range = -0.42) or SGRQ scores (N patients = 20, *R* = 0.42) [33, 59]. While one trial found an association between cough severity VAS and objective cough counts (N patients = 24, *R* = 0.683), another found no association between cough severity VAS and objective polygraphy-derived indices of cough [27, 33]. In observational studies in majority cough populations, cough severity VAS was similarly associated with LCQ scores (N patients = 27, Rho = − 0.72) and objective cough counts (N patients = 19, *R* = 0.80) and found to be an independent predictor of SGRQ scores (N patients = 516, β = 0.20) [37, 38, 40]. Correlations were also found between ER-S cough domain scores and EuroQol EQ-5D indices of pain/discomfort and anxiety/depression (N patients = 168, R range = 0.23–0.26) and between LCQ scores and Beck’s Depression Inventory (BDI) scores (N patients = 98, *R* = − 0.57) [42, 53].

Three studies reported longitudinal associations between cough, HRQoL and/or other impact measures: the trial of PA101 in patients with IPF and chronic cough, which found associations between change in cough severity VAS and change in daytime cough frequency (N patients = 23, *R* = 0.415) [27]; a *post hoc* trial analysis that found correlations between change in CASA-Q and SGRQ domain scores (N patients = 1061, R range = − 0.29 to − 0.45) [51]; and an observational study in the ‘broader includes’ category which found no association between change in LCQ scores and physical activity decline over 12 months (N patients = 54) [44].

#### IPF: economic burden of cough

No studies on the economic burden of cough in IPF were identified.

#### IPF: experiences of cough

Four qualitative studies in IPF used focus groups or interviews to gain patient and/or caregiver perspectives on symptoms and their burden [55–58]. Across these four studies (N patients = 83), cough emerged as one of the most troubling symptoms, with wide-ranging impact. Patients described relentless coughing throughout the day, leading to feelings of ‘exhaustion’ [56–58]. Although some patients felt that oxygen therapy helped alleviate their cough, coughing was described as ‘incredibly bothersome’ during the morning and evening, often occurred during exertion, and caused sleep disruption and incontinence [53, 54, 56–58]. Caregivers described feelings of distress, being perpetually vigilant and witnessing ‘draining coughing fits’ with a sense of helplessness [56, 58].

### Studies in patients with various ILDs and ILDs other than IPF

#### ILD: study characteristics and measures

Among the 18 studies in mixed ILD populations, there were two interventional trials [60, 61], seven observational studies [7, 62–67], four validation studies [68–71], four qualitative studies [72–75]. and one mixed-methods study [76]. Of the six studies in CTD-ILDs, there were three interventional trials in SSc-ILD [77–79], one observational study in a mixed CTD-ILD population [80], one validation study in SSc-ILD [81], and one mixed-methods study in a mixed CTD-ILD population [82]. Of the four studies for sarcoidosis, there was one interventional trial [83] and three observational studies [84–86]. Tables 3, 4 and 5 respectively provide an overview of the quantitative and mixed-methods studies in mixed ILDs, CTD-ILDs and sarcoidosis.

**Table 3:** Quantitative and mixed-methods studies in mixed ILD

Author year	Cough category	Study design	Arm	Disease severity (FVC % pred.)	Cough severity measures	HRQoL/impact measures	Group comparisons	Burden of cough
**Interventional trials**								
Bassi 2021	Broader includes	Open label RCT	Fibrosing ILD: 50	69.6 (2.15)	VAS: 52.3 (32.8)	MRQr: 11.6 (6.0)	VAS cough worsened both in intervention and usual care group (p-value group effect: 0.88)	Depression (CES-D) and dyspnoea (MRQr) were improved by the intervention, but cough was not
						CES-D: 13.8 (8.1)		
Sato 2021	Chronic cough	Pre-post intervention study	CTD-ILD, IIP: 11	77.8 (63.9–88.1)	VAS: 63 (35–79)	LCQ (acute): 13.4 (11.0–14.9)	–	Correlation not presented
**Observational studies**								
Cheng 2017	Chronic cough	Cohort study	IPF: 77	73.2 (18.1)	VAS: no baseline	SGRQ: no baseline	Cough was most prevalent in IPF and most productive in CHP	Cough severity was an independent predictor of total SGRQ after adjustments in SSc-ILD and IPF but not CHP
			CHP: 32	67.0 (19.8)				
			SSc-ILD: 67	74.6 (22.0)				
Lan 2021	Chronic cough	Cross-sectional study	ILD with cough: 118	74.6 (18.7)	VAS: 41.8 (25.9)	LCQ: 14.9 (4.3)	Prevalence of cough was highest in IPF, NSIP and sarcoidosis patients (> 70%)	Cough ranked as worse ILD symptom in over a third of patients
						Physical: 5.1 (1.5)		
			ILD with no cough: 46	87.0 (15.9)		Physiological: 5.3 (1.7)		
						Social: 4.5 (1.5)		
Minuk 2023	Broader includes	Cohort study	ILD: 102	46 (12)		ESAS cough score: 7 (IQR 4–9)	Cough was worse in patients with ILD at baseline and they had lower drowsiness scores compared with patients with COPD	–
			COPD: 24	NR		ESAS cough score: 4 (IQR 1–7)		
Sato 2019	Minority cough	Cross-sectional study	IIPs (incl IPF: 70	85.2 (74.2–97.3)	VAS: 31 (17–55)	LCQ: 19.3 (IQR:17.5–20.4)	Patients with the IIPs had the greatest intensity of cough but not frequency of cough	Patients in whom cough frequency was predominant had a greater impairment of health status relative to other patients. Significant correlation between total LCQ scores and intensity and frequency of cough were −0.675 and −0.762, respectively
			CTD-ILD: 49	93.6 (80.2–106.1)	VAS: 24 (8–46)	LCQ: 18.7 (IQR:15.4–20.5)		
			CHP: 10	73.6 (68.8–93.3)	VAS: 18 (6–20)	LCQ: 19.6 (IQR:18.3–20.6)		
Veit 2023	Majority cough	Prospective cohort	Non-IPF ILD: 22	63.4 (23.5)	VAS: 2.5 (2.4)*	SGRQ: 48.9 (20)	Patients with IPF not only had a higher burden of cough at the beginning of the study, but also experienced a greater increase in cough over time than those with non-IPF ILD. Patients with IPF had significantly more limitations in terms of KBILD values compared to those with non-IPF ILD; p = 0.022). SGRQ did not show significant differences between IPF and non-IPF ILD; p = 0.193)	For KBILD, but not SGRQ, there was a significant inverse correlation with VAS cough
						KBILD: 53.1 (12.1)		
			IPF: 13	68.5 (18.7)	VAS: 4.6 (2.7)*	SGRQ: 51.1 (9.8)		
						KBILD: 48.2 (2.6)		
Yuan 2020	Broader includes	Cross sectional, longitudinal and prospective study	IIP: 139	86.9 (22.2)	–	LCQ: 16.7 (3.7)	Average cough scores were comparable between IIP and CTD-ILD, HRQoL was lower in CTD-ILD	Cough impact correlates with quality of life at baseline and over time
						Physical: 5.4 (1.3)		
						Physiological: 5.5 (1.3)		
						Social: 5.8 (1.3)		
						SGRQ: 32.9 (19.1)		
						HADS-A: 5.0 (3.0–7.0)		
						HADS-D: 5.0 (1.0–7.0)		
						**SF-36**		
						Physical: 37.2 (12.0)		
						Mental: 48.3 (11.6)		
			CTD-ILD: 30	74.4 (19.1)	–	LCQ: 16.3 (3.7)		
						Physical: 5.3 (1.3)		
						Physiological: 5.4 (1.3)		
						Social: 5.7 (1.3)		
						SGRQ: 43.3 (20.6)		
						HADS-A: 6.0 (3.0–9.0)		
						HADS-D: 5.5 (2.7–9.2)		
						**SF-36**		
						Physical: 31.1 (14.2)		
						Mental: 45.6 (11.1)		
**Validation studies**								
Nagata 2012	Broader includes	Validation study	ILD (excl. IPF): 55	72.7 (17.3)	–	LCQ: 97.5 (39–133)	–	Cough strongly contributes to quality of life
						SGRQ: 43.2 (0.0–83.9)		
						CAT: 13 (1–33)		
						HADS-A: 4 (0–15)		
						HADS-D: 4 (0–13)		
Pan 2019	Broader includes	Validation study	IPF: 20	NR	–	SGRQ:78.65 (10.84)	Cough domains were significantly worse in IPF than non-IPF ILD,p < 0.001)	Many aspects of HRQoL were impaired in IPF according to the cATAQ-IPF score
						cATAQ-IPF total: 287.90 (22.56)		
						Cough: 24.70 (4.66)		
			Non-IPF ILD: 72	NR	–	SGRQ: 57.47 (21.81)		
						cATAQ-IPF total: 250.74 (47.39)		
						Cough: 17.58 (7.80)		
Yates 2018	Broader includes	Validation study	IPF: 30	NR	VAS: 38 (20)	NR	No significant difference in cough severity at baseline or interval change between IPF and non IPF subgroups	VAS cough did not correlate with KBILD
			Non-IPF ILD: 67	NR	VAS: 44 (29)	NR		
			Total initial cohort: 64	82.5 (18.8)	VAS: 43 (26)	KBILD: 62.6 (21.4)		
			Total validation cohort: 31	88.9 (20.1)	VAS: 41 (30)	KBILD: 62.5 (22.7)		
Kirsten 2022	Broader includes	Validation study	IPF and NSIP: 200	NR	–	SGRQ: 38.8	–	Cough scale included in the quality-of-life tool
						QPF-scale total: 97.11		
						QPF-scale cough: 4.23		
**Mixed-methods studies**								
Paixão 2023	Broader includes	Mixed methods	ILD and IPF: 10	77.1 (4.4)	–	CASA-Q cough symptoms: 83.3 (75–100)	Cough symptoms improved after 12 weeks of intervention	Correlation with quality of life not presented
						CASA-Q cough impact: 100 (78.1–100)		
						SGRQ total: 48.6 (19.4)		
						CAT: 14.9 (8.4)		
						HADS-A: 5.3 (5)		
						HADS-D: 7.1 (4.5)		
**Economic study (observational design)**								
Algamdi 2019	Minority cough	Cross-sectional study	Fibrotic ILD employed: 148	74.8 (20)	VAS: no baseline	Estimated annual costs of productivity loss: CAN$11,610 per patient	The costs of productivity loss were comparable between employed male and female patients and between IPF and non-IPF patients ($11,737 vs $11,535).	Cough was an independent predictor of workplace productivity loss.
						Hours lost, mean (SE): 7.8 (0.9)		

Mean (SD) or median (range or IQR)

*Reported on alternative 0–10 cm scale

** Reported on alternative 100-point scale

CAN, Canadian dollar; CAT, COPD Assessment Test; cATAQ-IPF, Chinese version of the A Tool To Assess Quality of Life in Idiopathic Pulmonary Fibrosis; CES-D, Center for Epidemiologic Studies Depression Scale; CHP, chronic hypersensitivity pneumonitis; CTD-ILD, connective tissue disease-associated interstitial lung disease, FVC, forced vital capacity; HADS-A, Hospital Anxiety and Depression Scale Anxiety score; HADS-D, Hospital Anxiety and Depression Scale Depression score; HRQoL, health-related quality of life; IIP, idiopathic interstitial pneumonia; ILD, interstitial lung disease; IPF, idiopathic pulmonary fibrosis; IQR, interquartile range; KBILD, King’s Brief Interstitial Lung Disease; LCM Leicester Cough Monitor; LCQ, Leicester Cough Questionnaire; MRQr, Maugeri Respiratory Questionnaire; NR, not reported; NSIP, non-specific interstitial pneumonia, PF-ILD, progressive fibrosing interstitial lung disease; QPF, Quality of life in patients with idiopathic pulmonary fibrosis tool; RA, rheumatoid arthritis; RCT, randomised controlled trial; SE, standard error; SGRQ, St. George’s Respiratory Questionnaire; SSc, systemic sclerosis; VAS, visual analogue scale

**Table 4:** Quantitative and mixed-methods studies in CTD-ILD

Author year	Cough category	Study design	Arm	Disease severity (FVC % pred.)	Cough severity measures	HRQoL/impact measures	Group comparisons	Burden of cough
**Interventional trials**								
Theodore 2012/SLS	Majority cough	RCT	SSc-ILD with cough: 114	65.85 (11.16)	–	**SF 36** Mental: 47.88 (10.82) Physical: 29.65 (8.76)	Those with cough were analysed by severity and frequency of cough – mild (62%) moderate (32%) and severe (5%) and infrequent (62%), intermittent (33%) and persistent (6%)	Prescence of cough was significantly correlated with lower physical QoL, but severity of cough did not correlate significantly with any baseline variable
						HAQ-DI: 0.98 (0.68)		
			SSc-ILD without cough: 42	69 (10.5)	–	**SF 36** Mental: 48.62 (11.7) Physical: 38.47 (10.4)		
						HAQ-DI: 0.74 (0.63)		
Volkmann 2022	Majority cough	*Post hoc* analysis (RCT)	SSc-ILD with cough: 229	71.5 (16.1)	–	**Nintedanib group** SGRQ: 43.9 (18.8)	HRQoL scores worse in patients with cough	Cough correlates with fibrosis extent at baseline
			SSc-ILD with cough: 232			**Placebo group** SGRQ: 42.3 (20.3)		
			SSc-ILD without cough: 58	76.7 (18.3)	–	**Nintedanib group** SGRQ: 28.0 (20.7)		
			SSc-ILD without cough: 56			**Placebo group** SGRQ: 27.1 (19.4)		
Volkmann 2020/SLS II	Majority cough	*Post hoc* analysis (RCT)	SSc-ILD treated with cyclophosphamide: 73	66.5 (9.9)	–	LCQ: 16.7 (4)	Both drugs improved PRO scores	Baseline LCQ scores all correlated with the extent of quantitative radiographic fibrosis and ILD as well as with measures of cutaneous sclerosis. Change in LCQ scores did not correlate significantly with any objective measure of SSc-ILD disease severity
						SGRQ: 36.8 (17.5)		
						HAQ-DI: 0.7 (0.7)		
						**SF-36** Physical: 35.6 (9.8) Mental: 49.8 (10)		
			SSc-ILD treated with mycophenolate: 69	66.5 (8.3)	–	LCQ: 16.8 (4)		
						SGRQ: 37.3 (17.4)		
						HAQ-DI: 0.7 (0.6)		
						**SF-36** Physical: 36 (10) Mental: 49.1 (7.9)		
Tashkin 2016/SLS II	Majority cough	*Post hoc* analysis (RCT)	SSc-ILD: 142	66.5 (9.1)	–	LCQ:16.7 (4.0)	–	Cough correlates with fibrosis extent
						HAQ-DI: 0.7 (0.7)		
						**SF-36** Physical: 35.8 (9.9) Mental: 49.4 (9.0)		
Tashkin 2017/SLS II	Majority cough	*Post hoc* analysis (RCT)	SSc-ILD with frequent cough: 87	65.6 (8.8)	–	LCQ: 15.4 (3.7)	Study participants who reported FC at baseline (61.3%) reported significantly more dyspnoea, exhibited more extensive ILD on high-resolution CT, had a lower diffusing capacity for carbon monoxide, and reported more GERD symptoms than did those without FC	Cough-related quality of life significantly correlated with HRQoL both at baseline and over treatment time
						**SF 36** Mental: 48.8 (8.6) Physical: 35.0 (9.8)		
						HAQ-DI: 0.66 (0.57)		
			SSc-ILD without frequent cough: 54	67.8 (9.4)	–	**SF 36** Mental: 50.7 (9.6) Physical: 37.2 (9.9)		
						HAQ-DI: 0.80 (0.81)		
**Observational studies**								
Topcu 2021	Broader includes	Cross-sectional study	RA-ILD: 14	92 (28)	–	LCQ: 17.8 (5.1)	Compared to patients with CTD, patients with RA-ILD have worse HRQoL, as measured by the SGRQ and SF-36 physical functioning score; median scores of LCQ were similar	No correlation reported but concluded PROs may not differentiate ILD cough from non-ILD cough in RA/CTD
						SGRQ: 79.4 (8.7)		
						SF-36: 60.9 (13.7)		
			CTD-ILD (incl SSc-ILD): 25	91 (47)	–	LCQ: 18.5 (3.7)		
						SGRQ: 27.1 (26.5)		
						SF-36: 63.9 (21.9)		
			Total (all ILD patients): 39	91.5 (38.5)	–	LCQ: 18.4 (4.2)		
						SGRQ: 40.4 (57.6)		
						SF-36: 63.2 (19.4)		
**Validation study**								
Fisher 2019	Minority cough	Validation study	SSc-ILD: 73	73.9 (15.5)	–	LCQ: 17.5 (3.1)	–	The average LCQ score indicated mild cough and the scores did not correlate with the corresponding PROMIS domains even in patients who reported cough (41% of the cohort)
						SGRQ: 32.6 (19.0)		
						**SF-36** Physical: 35.9 (12.7) Mental: 46.6 (11.3)		
						PROMIS* Physical function: 41.4 (8.1) Social role: 45.9 (8.2) Anxiety: 52.5 (9.6) Depression: 51.2 (11.0) Fatigue: 56.4 (10.4) Pain Interference: 55.9 (11.0) Sleep Disturbance: 52.9 (11.0) Pain: 3.5 (2.7)		
**Mixed methods study**								
Mittoo 2015	Broader includes	Mixed-method design	CTD-ILD: 45	Available for 2 of 6 FGs: Mixed: 55 (45–67) SSc: 46 (23–75)	Post-focus group questionnaire	WHOQOL-100: NR	FG interviews and subsequent quantitative self-administered questionnaire	Cough a hallmark symptom of ILD affecting social and physical QoL

* All reported on a 0–100-point scale aside from pain, which is reported on a 0–10 scale

CT, computed tomography; CTD-ILD, connective tissue disease-associated interstitial lung disease; FC, frequent cough; FG, focus group; FVC, forced vital capacity; GERD, gastroesophageal reflux disease; HAQ-DI, Health Assessment Questionnaire Disability Index; HRQoL, health-related quality of life; IQR, interquartile range; LCQ, Leicester Cough Questionnaire; NR, not reported; PRO, patient-reported outcome; QoL, quality of life; RA-ILD, rheumatoid arthritis-associated interstitial lung disease; RCT, randomised controlled trial; SF-36, Short Form 36; SGRQ, St. George’s Respiratory Questionnaire; SLS, Scleroderma Lung Study; SSc-ILD, systemic sclerosis interstitial lung disease; WHOQOL, World Health Organization Quality of Life tool

**Table 5:** Quantitative and mixed-methods studies in sarcoidosis

Author year	Cough category	Study design	Arm	Disease severity (FVC % pred.)	Disease severity (FVC % pred.)	HRQoL/impact measures	Group comparisons	Burden of cough
**Interventional trials**								
Fraser 2020	Chronic cough	Open label single-arm trial	21	91.5 (63–128)	HACC 24-hr cough count: 228 (43–1950) Coughs per hour: 10 (2–81)	LCQ: 14.63 (4.07)	Baseline cough count was significantly higher in patients with baseline cough severity VAS > 40 mm compared with VAS < 40 mm	Changes in cough counts correlated with changes in LCQ and KSQ GH but not with KSQ lung domain scores
					VAS: 38.8 (25.7) Urge to cough VAS: 38.7 (26.2)	KSQ: 57.3 (9.1) KSQ GH: 52.93 (18.3) KSQ Lung: 52.0 (10.4)		
**Observational**								
Sinha 2016	Majority cough	Cross-sectional	Patients with cough: 17		VAS: 53 (20–66)	LCQ: 14.8 (3.7) Physical: 4.8 (1.3) Psychological: 5.0 (1.3) Social: 5.0 (1.5)	Cough frequency was significantly higher than healthy subjects, but less than that reported in patients with idiopathic chronic cough	Cough (both 24-hr counts and VAS) was significantly associated with health status, affecting all LCQ health domains
					LCM: 24 h cough count: 244 (2) Coughs per hour: 10 (2)	CHQ: 10 (5–14)		
					Cough reflex sensitivity (C_5_ μmol·L^− 1^): 6.8 (3.2)			
			All patients: 32	83.7 (16.8)	LCM 24-hr cough count: 67 (5)	CHQ: 6 (3–12)		
					Cough reflex sensitivity (C_5_ μmol·L^− 1^): 13.3 (4.5)			
			Healthy subjects: 40	100.9 (29)	LCM 24 h cough count: 18 (3)	NR		
					Cough reflex sensitivity (C_5_ μmol·L^− 1^): 61.5 (6.5)			
Judson 2017	Minority cough	Cohort study	All patients: 355	No baseline	VAS: 2.5 (1.5)*	LCQ: 17.5 (3.0) Physical: 5.9 (1.3) Psychological: 5.9 (1.3) Social: 5.3 (0.9)	Cough was significantly worse in patients with pulmonary involvement compared to those without	Cough severity measured by VAS correlated significantly with LCQ domains except social. Those with VAS scores > 4 had higher total LCQ scores than those with VAS scores < 4
			Pulmonary sarcoidosis: 287		VAS: 2.6 (2.8)**	LCQ: 16.9 (3.5) Physical: 5.7 (1.5) Psychological: 5.9 (1.5) Social: 5.2 (1)		
			Non-pulmonary sarcoidosis: 54		VAS: 1.3 (2.2)	LCQ: 18.8 (3) Physical: 6.3 (1.3) Psychological: 6.5 (1.2) Social: 5.9 (1)		
Gvozdenovic 2020	Minority cough	Cohort study	275	108.52 (17.40)	–	LCQ: 16.94 (3.68) Physical: 5.48 (1.18) Psychological: 5.64 (1.29) Social: 5.82 (1.33)	-	Dyspnoea was the strongest predictor of cough-specific and generic QoL and the physical domain of the LCQ was a significant predictor of QoL
						15D: 0.85 (0.11)		

*Reported on alternative 0–10 cm scale

**Data amended following author correspondence [87]

15D; fifteen-dimensional measure of health-related quality of life; CHQ, Cough Hypersensitivity Questionnaire; FVC, forced vital capacity; GH, General Health score; HACC, The Hull Automatic Cough Counter; HRQOL, health-related quality of life; KSQ, King’s Sarcoidosis Questionnaire; LCM, Leicester Cough Monitor; LCQ, Leicester Cough Questionnaire; NR, not reported; QoL, quality of life; VAS, visual analogue scale

Of the studies in mixed ILDs, four were classed as chronic cough studies, compared with none in CTD-ILDs and one in sarcoidosis. Across these studies, definitions for chronic cough included self-reported chronic cough [62, 83] cough for > 8 weeks [7, 72], and cough intensity and frequency VAS > 10 mm [61].

Similar to the studies in IPF, studies in ILD used a range of measures to assess cough (Tables 3, 4 and 5). While cough severity VAS was the most frequently used cough measure in mixed ILDs (N studies = 9 [7, 60–63, 65, 67, 70, 72], N patients = 473; reported as mean score range 25–52.3, reported as median score range 18–63) and sarcoidosis (N studies = 3 [83–85], N patients = 393; mean score range 25–53), VAS was not used to assess cough in CTD-ILDs; instead, most CTD-ILD studies relied on cough items or domain scores from other measures, including the SGRQ and the LCQ. Objective cough counts were absent in studies in mixed ILDs and CTD-ILDs and were only employed in two studies in sarcoidosis (N patients = 93 [83, 84], mean count/hr range 6–244). The most frequently used HRQoL measure was the SGRQ (mixed ILD: N studies = 7 [62–64, 68, 69, 71, 76], N patients = 693, mean score range 32.9–78.65; CTD-ILD: N studies = 4 [78–81], N patients = 829, reported as mean score range 27.1–43.9, reported as median score range 27.1–79.4; sarcoidosis: 0 studies) followed by the LCQ (mixed ILD: N studies = 5 [7, 61, 64, 65, 68], N patients = 528, reported as mean score range 14.9–16.7, reported as median score range 18.7–19.6; CTD-ILD: N studies = 3 [78, 80, 81], N patients = 254, reported as mean score range 16.7–17.5, reported as median score 18.5; sarcoidosis: N studies = 4 [83–86], N patients = 668, mean score range 14–17.5).

#### ILD: comparisons with other populations

Only one study comparing cough in various types of ILD with other populations was identified; it found cough severity to be higher in patients with ILD than in those with COPD, as measured by Edmonton symptom assessment scores [66]. Seven studies in mixed ILD populations compared cough and/or impact/HRQoL measures among various ILD types ( [7, 62–65, 69, 70], Tables 3, 4 and 5). Four of these studies found the prevalence and/or severity of cough to be higher in patients with IPF or idiopathic interstitial pneumonias (IIPs) [7, 62, 63, 65], two reported worse HRQoL in IPF (particularly in cough-related domains) [63, 69], and one observed greater increase in cough severity over time in IPF [63]. Conversely, one study observed no differences in cough severity between IPF and non-IPF groups [70] and in another HRQoL was lower in CTD-ILD than IIPs (including IPF) [64]. Additionally, two studies comparing patients with IIPs – including IPF – with patients with CTD-ILDs, found no between-group differences in LCQ scores [64, 65]. However, one of these studies reported higher VAS scores for cough intensity in patients with IIPs [65].

In CTDs specifically, one observational study compared cough measures in patients with RA and other CTDs with and without associated ILD [80]. The study found SGRQ scores to be worse in patients with RA-ILD compared with other CTD-ILDs, although there were no differences in LCQ scores. Further, while LCQ scores did not differ between patients with and without associated ILD, SGRQ outcomes were worse in those without associated ILD. In sarcoidosis, one observational study found objective cough counts and cough reflex sensitivity to be higher in patients than healthy controls [84].

#### ILD: association of cough with impact/HRQoL measures

In mixed-ILD populations, six studies investigated concurrent/baseline associations between cough, HRQoL and/or other impact measures [62–65, 68, 70], with five of these reporting at least one significant association ([62–65, 68], Tables 3, 4 and 5). These included an observational study in patients with chronic cough, which found cough severity VAS to be an independent predictor of SGRQ total and/or domain scores in patients with IPF (N patients = 77, *R* range = 0.33–0.55) and SSc-ILD (N patients = 67, *R* range = 0.34–0.51) but not in chronic HP (N patients = 32, *R* range = -0.10–0.03) [62]. Conversely, while an observational study in a majority cough population with fibrotic ILDs observed a significant association between cough severity VAS and King’s Brief Interstitial Lung Disease (KBILD) scores (N patients = 35, *R* = − 0.54), no such association was observed with SGRQ scores [63]. In mixed ILD studies in the ‘broader includes’ category, one observational study and one validation study found LCQ scores to be correlated with total SGRQ scores (N patients = 139, β=–3.55; N patients = 55, *R* = − 0.70, respectively) [64, 68], with the observational study also finding a correlation with Short Form 36 (SF-36) physical component scores (N patients = 139, β = 0.55–1.34) [64].

In CTD-ILD, a trial in a majority cough population with SSc-ILD similarly observed a correlation between total LCQ and SF-36 physical component scores (N patients = 87, *R* = 0.258) [88]. Three trials in SSc-ILD also reported correlations between the presence of cough (frequency) and cough impacts (as measured by LCQ scores) and measures of disease severity (extent of fibrosis, cutaneous sclerosis and/or dyspnoea) [77, 79, 88]. However, a study validating the Patient-Reported Outcomes Measurement Information System 29 tool (PROMIS-29) in SSc-ILD found no correlations between PROMIS domains and LCQ social or psychological domain scores, although there was a correlation with LCQ physical domain scores (N patients = 73, *R* = 0.36) [81]. In sarcoidosis, three studies investigated and observed correlations between total LCQ scores and cough severity VAS scores (N patients = 355, *R* = − 0.83), objective cough counts (N patients = 32, *R* = − 0.61) and/or 15-dimension HRQoL scores (N patients = 275, β = 0.24) [84–86].

Only two mixed ILD studies investigated longitudinal associations between cough, HRQoL and/or other impact measures. These included an observational study where correlations were observed between changes in LCQ scores and SGRQ scores at 6 and 12 months (N patients = 147, *R* = − 0.56 to − 0.58) [64] and a validation study where changes in cough VAS scores at 3–6 months were found to correlate with KBILD total scores (N patients = 64, *R* = − 0.36), but not KBILD cough domain scores or measures of disease severity [70]. Further, the validation study found that cough VAS was unable to detect changes in cough symptoms over time, as assessed by KBILD cough domain scores [70]. In CTD-ILD, the only trial investigating longitudinal associations between cough and impact/HRQoL measures found correlations between 24-month changes in LCQ scores and SF-36 physical scores (N patients = 142, *R* = 0.54) and mental component scores (*R* = 0.45) as well as Health Assessment Questionnaire Disability Index scores (*R* = − 0.03) [88]. In sarcoidosis, a trial investigating longitudinal associations observed correlations between 3-month changes in objective cough counts and LCQ scores (*N* = 19, *R* = − 0.64) and King’s Sarcoidosis Questionnaire general health scores (*R* = − 0.59) [83], while an observational study reported a significant correlation in individual patients between changes in cough VAS scores and the LCQ between clinic visits (N = 891, R = not reported, *p* < 0.0001) [85]. Overall, this heterogeneity of results demonstrates that while cough may impact a patient’s HRQoL, available assessment tools are sensitive to different changes and do not always correlate. This means a broad range of assessments may be necessary to capture the multifaceted dimensions of cough until a standardised and disease-specific cough assessment tool can be developed and validated.

#### ILD: economic burden of cough

No studies specifically investigating the economic burden of cough were identified in ILD populations. However, one observational study in patients with fibrotic ILDs, including IPF, IIP and unclassifiable ILD, found an association between cough severity and workplace productivity loss [67]. Specifically, the study showed that the odds of productivity loss (N = 148) increased by 3% for every 1 mm increase in cough severity VAS, with an estimated annual cost of CAN$11,610 per employee.

#### ILD: experiences of cough

Four qualitative studies in mixed ILD populations (N patients = 63) explored symptoms and their impact on patients through interviews or focus groups [72–75]. Across these studies, patients identified cough as a significant symptom, describing it as ‘debilitating’, ‘hard to control’ and ‘embarrassing’ [72]. They reported that coughing not only led to fatigue but also hampered their daily activities and caused social discomfort and frustration/irritability [72–75]. In one of the studies, cough was also described as being especially disruptive to caregivers [73]. These experiences were mirrored in a mixed-methods study in patients with CTD-ILD, where cough was noted to negatively affect physical function, social participation, daily activities and sleep quality [82]. In sarcoidosis, no studies on patient experiences were identified.

## Discussion

Our study is the first to systematically synthesise literature on the humanistic and economic impact related to cough in IPF and other ILDs. The findings confirm that cough is a pervasive and persistent symptom in many patients with IPF and other ILDs.

Quantitative studies in patients with IPF consistently demonstrated the detrimental effect of cough on HRQoL, while qualitative studies in this population highlighted the significant cough-related burden experienced by patients and their caregivers, including disruption of daily activities, sleep deprivation, fatigue, incontinence, social embarrassment and psychological distress.

Indications of similar impact were reported in patients with CTD-ILDs, sarcoidosis, and other ILDs, but data were more limited. While no dedicated studies on the economic burden or healthcare resource use associated with cough were found, one study in ILD indicated that cough severity significantly predicted workplace productivity loss [67]. Studies in other populations with chronic cough suggest substantial economic burden related to increased healthcare utilisation, challenges in the workplace, and cough-related comorbidities [20], underscoring the need for more research in this area in IPF and ILD.

Across disease groups and study designs (with the exception of CTD-ILD), cough severity VAS was the most frequently used measure of cough, despite its lack of validation in patients with IPF and ILD. Objective cough monitoring devices were rarely used outside of trials in patients with IPF and chronic cough. While score ranges were wide, studies enriched for patients with IPF and chronic cough reported the highest mean VAS scores (56.0–73.7 mm). Conversely, lower mean/median scores were generally observed in studies in patients with IPF where cough chronicity was not specifically reported (reported as mean 30.5 mm; reported as median 32.0–40.3 mm) and mixed ILD (reported as mean 25.0–52.3 mm; reported as median 18.0–31.0 mm) populations.

Beyond cough characteristics, differences in patient populations, disease severity and treatments may have contributed to the variability observed in cough severity and duration.

Notably, cough severity VAS scores were variable even within the different chronic cough cohorts, which may relate to between-study differences in definitions of chronic cough as well as variability in study designs, populations and the tools used to assess cough. While the 8-week minimum duration was the most frequently used criterion, the use of additional thresholds based on subjective and/or objective criteria varied considerably, mirroring challenges in other populations with chronic cough and highlighting the need for consensus-driven criteria to ensure consistency. Future studies should aim to establish uniform inclusion criteria and standardised assessment tools for chronic cough to enhance the comparability of results.

Irrespective of the presence of chronic cough, in most studies that directly compared patients with IPF with other ILDs, the prevalence and severity of cough tended to be greater in patients with IPF. Outside of IPF, there were few studies specifically focussed on cough burden and impact. In most cases, cough-related data were collected as secondary measures in larger studies, and there was substantial heterogeneity in the tools used. Various cough-related HRQoL tools, such as the LCQ, CQLQ and CASA-Q were used alongside broader ILD-specific HRQoL tools such as the KBILD, Living with Pulmonary Fibrosis (L-PF) questionnaire and the A Tool to Assess Quality of Life in Idiopathic Pulmonary Fibrosis (ATAQ-IPF) questionnaire. Studies also used more general HRQoL tools, such as the SGRQ, Functional Assessment of Chronic Illness Therapy (FACIT) and EQ-5D as well as measures focussed on psychological distress, including the BDI. Even when the same tool was used, there was variability in the versions and scales used as well as in the reported statistics, exacerbating discrepancies in the synthesised data. While the LCQ was the most frequently used impact measure for cough both in IPF and ILD, it was not specifically designed for these populations and may not capture the full impact of cough in these contexts.

The divergence in approaches may be related to the absence of a widely accepted and validated cough-specific measure for IPF and ILD. Development of a standardised and disease-specific cough assessment tool, alongside validation of existing tools in these populations, could facilitate more consistent and reliable between-study comparisons. In addition, establishing such tools could pave the way for future advancements in personalised medicine, with treatments guided by the severity of cough in IPF and ILD.

A notable strength of our study is its comprehensive search strategy, which allowed for in-depth examination of the full spectrum of impact related to cough not only in IPF but also other ILDs. The high level of heterogeneity in the included studies and relative low number of studies in ILDs are significant limitations, which posed challenges for bias assessment and direct comparisons. Differences in study designs, patient populations, disease severity and assessment tools also limit the generalisability of the results. Other limitations include the exclusion of letters, graphical abstracts, case series and articles published before 2010, which may have resulted in certain aspects of cough burden and impact being missed. Despite these challenges, our study offers valuable insights into the current state of research on the burden of cough in IPF and other ILDs, emphasising the importance of standardisation to advance knowledge in this area.

The lack of specific treatments and management for cough in these patients remains a significant unmet clinical and patient need. Evidence for impact of different therapeutic approaches on cough is limited, with many of the trials negative or requiring confirmation in larger studies [7, 89]. We hope by highlighting the impact and breadth and magnitude of the burden of cough in patients with IPF and ILD we can encourage further research.

## Conclusions

In conclusion, studies consistently confirm a negative effect of cough on HRQoL in IPF, with indications of a similar impact in other ILDs, though less well studied. However, differences in definitions and assessment methods across studies hinder meaningful comparisons and there is a notable lack of research on the economic burden of cough in both IPF and other ILDs. Establishing standard measures for cough assessment in IPF and other ILDs is vital to enhance understanding of cough and inform future research, treatment and care models.

## Electronic supplementary material

Below is the link to the electronic supplementary material.

Supplementary Material 1

Supplementary Material 2

## Data Availability

All of the data described in this review are available in the cited articles.

## Abbreviations

BDI: Beck’s Depression Inventory
CASA-Q: Cough and Sputum Assessment Questionnaire
CDSR: Cochrane Database of Systematic Reviews
CENTRAL: Cochrane Central Register of Controlled Trials
CIC: Chronic idiopathic cough
CQLQ: Cough Quality of Life Questionnaire
CTD-ILD: Connective tissue disease-associated ILD
HP: Hypersensitivity pneumonitis (HP)
HRQoL: Health-related quality of life
IIP: Idiopathic interstitial pneumonia
ILD: Interstitial lung disease
IPF: Idiopathic pulmonary fibrosis
KBILD: King’s Brief Interstitial Lung Disease
LCQ: Leicester Cough Questionnaire
NHS EED: National Health Service Economic Evaluation Database
PF-ILD: Progressive-fibrosing ILD
PPF: Progressive pulmonary fibrosis
RA-ILD: Rheumatoid arthritis-associated ILD
SF-36: Short Form 36
SGRQ: St. George’s Respiratory Questionnaire
SSc-ILD: Systemic sclerosis associated-ILD
VAS: Visual analogue scale
