# Correction: The burden of cough in idiopathic pulmonary fibrosis and other interstitial lung diseases: a systematic evidence synthesis

**DOI:** 10.1186/s12931-025-03227-4

**Published:** 2025-05-15

**Authors:** Rhiannon Green, Michael Baldwin, Nick Pooley, Kate Misso, Maureen PMH Rutten-van Mölken, Nina Patel, Marlies S. Wijsenbeek

**Affiliations:** 1 Market Access, Maverex Limited, Manchester, UK; 2 Value and Patient Access, Boehringer Ingelheim International GmbH, Ingelheim am Rhein, Germany; 3 Erasmus School of Health Policy and Management, Erasmus University Rotterdam, Rotterdam, The Netherlands; 4 Inflammation Medicine, Boehringer Ingelheim Pharmaceuticals Inc, Ridgefield, CT USA; 5 Respiratory Medicine, Pulmonary Medicine, Erasmus Medical Center, University Medical Center Rotterdam, ‘s-Gravendijkwal 230, Rotterdam, 3015 CE The Netherlands

**Correction to: Respiratory Research (2024) 25:325**

10.1186/s12931-024-02897-w

The original publication of this article [1] contained errors in the content of Tables 2 and 3. The following errors were reported. In Table 2, the row header “Mixed-methods” was incorrect and should have been “Mixed-methods studies”.

In Table 2, “FVC % pred. 80.6; FVC % pred. 19.9” was incorrect and should have been “FVC % pred. 80.6 (19.9)”.

In Table 3, the row header “Mixed methods studies” was incorrect and should have been “Mixed-methods studies”.

The original article has been corrected.
