# Supplementary material for: The burden of cough in idiopathic pulmonary fibrosis and other interstitial lung diseases: a systematic evidence synthesis
Source: Respir Res. 2024 Aug 27;25:325. doi: 10.1186/s12931-024-02897-w (PMC11351049; doi:10.1186/s12931-024-02897-w)
Supplement: Supplementary file 1 — Supplementary Material 1 [file 12931_2024_2897_MOESM1_ESM.docx]

**The burden of cough in idiopathic pulmonary fibrosis and other interstitial lung diseases: a systematic evidence synthesis**

Rhiannon Green,^1^ Michael Baldwin,^2^ Nick Pooley,^1^ Kate Misso,^1^ Maureen PMH Rutten-van Mölken,^3^ Nina Patel,^4^ Marlies S Wijsenbeek^5^

^1^Market Access, Maverex Limited, Manchester, United Kingdom
^2^Value and Patient Access, Boehringer Ingelheim International GmbH, Ingelheim am Rhein, Germany
^3^Erasmus School of Health Policy and Management, Erasmus University Rotterdam, Rotterdam, The Netherlands
^4^Inflammation Medicine, Boehringer Ingelheim Pharmaceuticals Inc, Ridgefield, CT, USA
^5^Respiratory Medicine, Erasmus Medical Center, Rotterdam, The Netherlands

**Corresponding author**: Marlies S Wijsenbeek

**Address:** Erasmus MC: University Medical Center Rotterdam, Pulmonary Medicine, Gravendijkwal 230, Rotterdam 3015 CE, Netherlands

**Email:** [m.wijsenbeek-lourens@erasmusmc.nl](mailto:m.wijsenbeek-lourens@erasmusmc.nl)

[**1. Supplementary Methods** 2](#_Toc150352479)

[1.1 Strategy for initial search: August 2022 2](#_Toc150352480)

[1.2 Strategy for updated search: October 2023 19](#_Toc150352481)

# **Supplementary Methods**

## **Strategy for initial search: August 2022**

The following resources were initially searched for this systematic review:

| Database | Host | Date range | Date searched | Results |
| --- | --- | --- | --- | --- |
| Embase | Ovid | 2010-2022/08/31 | 1.9.22 | 1603 |
| Medline & In-Process Citations | Ovid | 2010-2022/08/31 | 1.9.22 | 333 |
| Medline Daily Update & Epubs Ahead-of-Print | Ovid | 2010-2022/08/31 | 1.9.22 | 11 |
| PubMed | NLM | 2010-2022/09/01 | 1.9.22 | 877 |
| Europe PMC, includes BioRxiv & medRxiv | www | 2010-2022/09/01 | 1.9.22 | 129 |
| Cochrane Database of Systematic Reviews (CDSR) | Wiley | 2010-2022/09/01 | 1.9.22 | 264 |
| Cochrane Central Register of Controlled Trials (CENTRAL) | Wiley | 2010-2022/09/01 | 1.9.22 | 3300 |
| NHS Economic Evaluation Database (NHS EED) | CRD | 2010-2015/03/31 | 1.9.22 | 27 |
| **Total retrieved** |  |  |  | **6544** |
| **Total screened** |  |  |  | **4905** |
| **Duplicates** |  |  |  | **1639** |

**Embase (Ovid): 2010-2022/08/31**

**Searched 1.9.22**

1 Productivity/ 46025

2 Absenteeism/ 19014

3 Caregiver Burden/ 9621

4 Caregiver/ 101992

5 Work Disability/ 5576

6 ((human$ or social$ or societ$ or work$ or employe$ or business$ or communit$ or famil$ or carer$ or caregiver$) adj3 (burden$ or consequenc$ or impact$ or problem$ or productivity or sickness or impairment$)).ti,ab,ot,hw. 207065

7 ((long standing or longstanding or long term or longterm or permanent or employee$) adj2 (absence$ or absent$ or ill$ or sick$ or disab$)).ti,ab,ot,hw. 17363

8 (llsi or ((emergenc$ or domestic$ or famil$ or carer$ or caregiver$) adj3 leave$)).ti,ab,ot. 1326

9 ((cost or costs or burden) adj2 (illness$ or disease$ or sickness$)).ti,ab,ot,kw,kf. 62460

10 ((allowance or status or long-term or pension$ or benefit$) adj2 (disab$ or incapacit$)).ti,ab,ot,hw. 34113

11 ((unable or inability or incapacit$ or incapab$) adj3 work).ti,ab,ot,hw. 3078

12 ((resource$ or fund$) adj2 (use$ or utili?ation)).ti,ab,ot,hw. 60581

13 ((health or healthcare) adj2 (resource$ or fund$)).ti,ab,ot,hw. 54585

14 ("length of stay" or "duration of stay" or "extended stay" or "prolonged stay" or "stay days").ti,ab,ot,kw,kf. 142429

15 ((ambulatory or ambulance or hospital or A&E or emergency) adj2 (attention$ or trip or trips or visit$ or stay$ or admission$ or admitted or transport$)).ti,ab,ot,hw. 506430

16 ((GP or general practitioner$ or doctor$ or inpatient$ or in-patient$ or clinician$ or specialist$ or physician$ or clinic or clinics or outpatient$ or out-patient$ or surger$) adj2 (appointment$ or attention$ or trip or trips or visit$)).ti,ab,ot,kw,kf. 81100

17 ((hospital$ or inpatient$ or in-patient$) adj2 (stay$ or admission$ or episode$)).ti,ab,ot,kw,kf. 289654

18 ((length$ or hospital$ or inpatient$ or in-patient$ or ICU or HDU or "intensive care" or "high dependancy") adj2 stay$).ti,ab,ot,kw,kf. 318446

19 ((outpatient$ or outpatient$ or inpatient$ or in-patient$ or ambulatory or emergenc$) adj2 care).ti,ab,ot,kw,kf. 96961

20 (cost$ adj2 (burden$ or estimat$ or variabl$ or hospital$ or control$)).ti,ab,ot,kw,kf. 77296

21 (district nurs$ or health visitor$ or health visiting or community nurs$ or community care or community visit$ or home visit$).ti,ab,ot,kf,kw. 29055

22 (in-patient stay$ or inpatient stay$).ti,ab,ot,hw. 7078

23 ((ambulatory or ambulance or hospital or A&E or emergency) adj2 (attention$ or trip or trips or visit$ or stay$ or admission$ or admitted or transport$)).ti,ab,ot,kw,kf. 329259

24 or/1-17 1197288

25 health-economics/ 34623

26 exp economic evaluation/ 338122

27 exp health care cost/ 323232

28 exp pharmacoeconomics/ 221576

29 (econom$ or cost or costs or costly or costing or price or prices or pricing or pharmacoeconomic$).ti,ab. 1281086

30 (expenditure$ not energy).ti,ab. 47196

31 (value adj2 money).ti,ab. 2802

32 budget$.ti,ab. 44331

33 or/25-32 1674743

34 ((metabolic adj cost) or (energy adj cost) or (energy adj expenditure)).ti,ab. 40100

35 33 not 34 1667085

36 exp quality of life/ 594833

37 (sf36 or sf 36 or sf-36 or short form 36 or shortform 36 or sf thirtysix or sf thirty six or shortform thirtysix or shortform thirty six or short form thirty six or short form thirtysix or short form thirty six).ti,ab,ot. 47058

38 (sf6 or sf 6 or sf-6 or short form 6 or shortform 6 or sf six or sfsix or shortform six or short form six).ti,ab,ot. 2779

39 (sf12 or sf 12 or sf-12 or short form 12 or shortform 12 or sf twelve or sftwelve or shortform twelve or short form twelve).ti,ab,ot. 11306

40 (sf6D or sf 6D or sf-6D or short form 6D or shortform 6D or sf six D or sfsixD or shortform six D or short form six D).ti,ab,ot. 1736

41 (sf20 or sf 20 or sf-20 or short form 20 or shortform 20 or sf twenty or sftwenty or shortform twenty or short form twenty).ti,ab,ot. 497

42 (sf8 or sf 8 or sf-8 or short form 8 or shortform 8 or sf eight or sfeight or shortform eight or short form eight).ti,ab,ot. 1126

43 "health related quality of life".ti,ab,ot. 77498

44 (Quality adjusted life or Quality-adjusted-life).ti,ab,ot. 24192

45 "assessment of quality of life".ti,ab,ot. 3336

46 (eq-5d or eq5d or eq-5 or eq5 or euro qual or euroqual or euro qual5d or euroqual5d or euro qol or euroqol or euro qol5d or euroqol5d or euro quol or euroquol or euro quol5d or euroquol5d or eur qol or eurqol or eur qol5d or eur qol5d or eur?qul or eur?qul5d or euro$ quality of life or european qol).ti,ab,kf. 27841

47 (euro$ adj3 (5 d or 5d or 5 dimension$ or 5dimension$ or 5 domain$ or 5domain$)).ti,ab,kf. 8124

48 (hql or hrql or hqol or h qol or hrqol or hr qol or hye or hyes).ti,ab,ot. 41908

49 ((cough or coughing) adj3 frequen$).ti,ab,ot,kf,kw. 2081

50 health$ year$ equivalent$.ti,ab,ot. 41

51 (hui or hui1 or hui2 or hui3 or hui4 or hui-4 or hui-1 or hui-2 or hui-3).ti,ab,ot. 3687

52 (quality time or qwb or quality of well being or "quality of wellbeing" or "index of wellbeing" or "index of well being").ti,ab,ot,hw. 1450

53 (Disability adjusted life or Disability-adjusted life or health adjusted life or health-adjusted life or "years of healthy life" or healthy years equivalent or "years of potential life lost" or "years of health life lost").ti,ab,ot. 6436

54 (QALY$ or DALY$ or HALY$ or YHL or HYES or YPLL or YHLL or qald$ or qale$ or qtime$ or AQoL$).ti,ab,ot. 31197

55 (qald$ or qale$ or qtime$).ti,ab,kf. 396

56 (timetradeoff or time tradeoff or time trade-off or time trade off or TTO or Standard gamble$ or "willingness to pay").ti,ab,ot. 15209

57 15d.ti,ab,ot. 2805

58 (HSUV$ or health state$ value$ or health state$ preference$ or HSPV$).ti,ab,ot. 720

59 (utilit$ adj3 ("quality of life" or valu$ or scor$ or measur$ or health or life or estimat$ or elicit$ or disease$ or cost$ or disease$ or mean or gain or gains or index$)).ti,ab,ot. 34026

60 (utilities or disutili$).ti,ab,ot. 14547

61 (illness state$1 or health state$1).ti,ab,kf. 13546

62 (multiattribute$ or multi attribute$).ti,ab,kf. 1407

63 Cost-Benefit Analysis/ and (cost-effectiveness ratio$ and (perspective$ or life expectanc$)).ti,ab,kf. 1122

64 ((quality of life or qol) adj (score$1 or measure$1)).ti,ab,kf. 33922

65 questionnaire/ or open ended questionnaire/ or structured questionnaire/ 851060

66 health status indicator/ or chronic disease indicator/ or disease activity score/ or "severity of illness index"/ 31471

67 health survey/ 216691

68 interview/ or semi structured interview/ or structured interview/ or exp telephone interview/ or unstructured interview/ 324566

69 (survey or surveys or questionnaire$ or interview or interviews or "focus group$").ti,ab,ot,kf,kw. 1977637

70 (PRO or PROs or patient reported outcome$).ti,ab,ot,kf,kw. 412518

71 (CRQ or PICQol or "CAP-sym" or "MRF26" or "MRF-26" or "AQ30" or "AQ-30" or "AQ20" or "AQ-20" or BPQ or CRQ or PFSDQ$ or SGRQ or LCQ or K-BILD or BILD).ti,ab,ot,kf,kw. 7281

72 or/36-71 3091987

73 or/24,35,72 5202216

74 animal/ or animal experiment/ 4427269

75 (rat or rats or mouse or mice or murine or rodent or rodents or hamster or hamsters or pig or pigs or porcine or rabbit or rabbits or animal or animals or dogs or dog or cats or cow or bovine or sheep or ovine or monkey or monkeys).ti,ab,ot,hw. 7324902

76 or/74-75 7324902

77 exp human/ or human experiment/ 24022192

78 76 not (76 and 77) 5532392

79 73 not 78 4956109

80 79 not (letter or editorial).pt. 4789471

81 coughing/ or barking cough/ or chronic cough/ or dry cough/ or experimental coughing/ or hacking cough/ or irritative coughing/ or paroxysmal cough/ or productive cough/ or psychogenic cough/ 142370

82 cough$.ti,ab,ot,kf,kw,hw. 172194

83 or/81-82 172194

84 exp interstitial lung disease/ 94575

85 ((Interstitial or diffuse parenchymal) adj3 lung adj2 (disorder$ or disease$)).ti,ab,ot,kf,kw. 27929

86 (ILD or ILDs or DPLD or IPD or IIP or IPF or DIP or AIP or NSIP or RBILD or RB-ILD or COP or BOOP or CWP).ti,ab,ot,kf,kw. 67167

87 pulmonary fibrosis.ti,ab,ot,kf,kw. 36135

88 ((Interstitial or diffuse parenchymal) adj3 pulmonary adj2 (disease$ or disorder$)).ti,ab,ot,kf,kw. 1580

89 (Interstitial adj3 (Pneumonia$ or Pneumonitides or Pneumonitis or pneumopath$ or pneumocystic)).ti,ab,ot,kf,kw. 18088

90 (chronic fibrous pneumonia$ or fibroid phthisis or phthisis fibroidea or pneumonia chronica fibrosa or pneumonia fibrosa chronica or pneumonia interstitialis or pneumonitis acuta).ti,ab,ot,kf,kw. 1

91 ((carrington$ or Loeffler$ or Loffler$ or caplan$ or Hamman-Rich$ or Antisynthetase or Anti-synthetase) adj2 (disease$ or disorder$ or syndrome$)).ti,ab,ot,kf,kw. 1877

92 (eosinophil$ adj3 (pleurisy or pleuritis or lung or lungs or loeffler$ or Loffler$ or pneumonia$ or infiltrate or pulmonary)).ti,ab,ot,kf,kw. 8517

93 ((wegener$ or polyangiitis or necrotising or respiratory or necrotizing or pneumogenic) adj2 granulomatosis).ti,ab,ot,kf,kw. 12779

94 (wegener$ adj2 (syndrome$ or disease$ or disorder$ or morbus or granuloma$ or churg or klinger)).ti,ab,ot,kf,kw. 7947

95 (lung coniosis or pneumoconiosis or pneumoconiotic or pneumokoniosis or pneumonoconiosis or pneumonokoniosis or silicoarthritis).ti,ab,ot,kf,kw. 4559

96 ((Cryptogenic organizing or Cryptogenic organising or bronchiolitis obliterans) adj3 pneumonia$).ti,ab,ot,kf,kw. 2288

97 ((thatched roof or maple bark or detergent worker$ or welder$) adj2 (disease$ or disorder$ or syndrome$)).ti,ab,ot,kf,kw. 61

98 ((coal miner$ or coalworker$ or worker$ or black or aluminium or welder$ or iron miner$ or iron-miner$ or labrador or bird fancier$ or indium or bird breeder$ or pigeon keeper$ or cheese washer$ or compost or farmer$ or hot tub or humidifier$ or Japanese summer house or maple bark or miller$ or peat moss$ or snuff or trombone Player$ or Brass Player$ or Wine-grower$ or woodworker$) adj2 lung$).ti,ab,ot,kf,kw. 2038

99 (Asbestosis or silicosis or anthracosis or Aluminosis or siderosis or Berylliosis or Byssinosis or Chalicosis or Silicosiderosis or Stannosis or Talcosis or Baritosis or bagassosis or Lycoperdonosis or Sequoiosis or Suberosis).ti,ab,ot,kf,kw. 11173

100 ((Bauxite or beryllium) adj2 (disease$ or disorder$ or poisoning or syndrome$ or fibrosis)).ti,ab,ot,kf,kw. 585

101 (pulmonary adj2 (haemosiderosis or haemo-siderosis)).ti,ab,ot,kf,kw. 164

102 ((Hypersensitivity or hyper-sensitivity) adj2 pneumonitis).ti,ab,ot,kf,kw. 4741

103 extrinsic allergic alveolitis.ti,ab,ot,kf,kw. 1059

104 ((Cephalosporium or Isocyanate or Trimellitic anhydride or TMA or Familial or Domestic or Japanese summer or Metalwork$ or Metal-work$ or Mollusc or shell or Sauna$ or worker$ or Streptomyces or Tap water) adj2 HP).ti,ab,ot,kf,kw. 62

105 Fibrosing alveolitis.ti,ab,ot,kf,kw. 1074

106 ((alveolar or lung$ or interstitial or pulmonary) adj3 fibrosis).ti,ab,ot,kf,kw. 65637

107 or/84-106 201819

108 80 and 83 and 107 1942

**109 limit 108 to yr="2010 -Current" 1603**

Humanistic burden terms based on:

Clayton S, Bambra C, Gosling R, Povall S, Misso K, Whitehead M. Assembling the evidence jigsaw: insights from a systematic review of UK studies of individual-focused return to work initiatives for disabled and long-term ill people [Internet]. BMC Public Health 2011 [cited 23.3.11];11:170.

Cost-effectiveness filter based on:

Centre for Reviews and Dissemination. Search strategies: NHS EED EMBASE using OvidSP (economics filter) [Internet]. York: Centre for Reviews and Dissemination; 2014 [accessed 2.6.14]. Available from:

http://www.crd.york.ac.uk/crdweb/searchstrategies.asp#nhseedembase

HRQoL free-text terms based on:

Figure 4: Common free-text terms for electronic database searching for HSUVs in Papaioannou D, Brazier JE, Paisley S. NICE DSU Technical Support Document 9: the identification, review and synthesis of health state utility values from the literature (Internet), 2011 (accessed: 18.8.11) Available from: http://www.nicedsu.org.uk

Arber M, Garcia S, Veale T, Edwards M, Shaw A, Glanville JM. Performance of Ovid Medline search filters to identify health state utility studies. Int J Technol Assess Health Care 2017;33(4):472-80.

**Medline & In-Process Citations (Ovid): 1946-2022/08/31**

**Searched 1.9.22**

1 Efficiency/ 15052

2 Absenteeism/ 9667

3 Caregiver Burden/ 444

4 Caregivers/ or Stress, Psychological/ or Financial Stress/ or exp Occupational Stress/ 189012

5 ((human$ or social$ or societ$ or work$ or employe$ or business$ or communit$ or famil$ or carer$ or caregiver$) adj3 (burden$ or consequenc$ or impact$ or problem$ or productivity or sickness or impairment$)).ti,ab,ot,hw. 149044

6 ((long standing or longstanding or long term or longterm or permanent or employee$) adj2 (absence$ or absent$ or ill$ or sick$ or disab$)).ti,ab,ot,hw. 12269

7 (llsi or ((emergenc$ or domestic$ or famil$ or carer$ or caregiver$) adj3 leave$)).ti,ab,ot. 1051

8 ((cost or costs or burden) adj2 (illness$ or disease$ or sickness$)).ti,ab,ot,kw,kf. 39444

9 ((allowance or status or long-term or pension$ or benefit$) adj2 (disab$ or incapacit$)).ti,ab,ot,hw. 15193

10 ((unable or inability or incapacit$ or incapab$) adj3 work).ti,ab,ot,hw. 2066

11 ((resource$ or fund$) adj2 (use$ or utili?ation)).ti,ab,ot,hw. 38458

12 ((health or healthcare) adj2 (resource$ or fund$)).ti,ab,ot,hw. 48687

13 ("length of stay" or "duration of stay" or "extended stay" or "prolonged stay" or "stay days").ti,ab,ot,kw,kf. 74179

14 ((ambulatory or ambulance or hospital or A&E or emergency) adj2 (attention$ or trip or trips or visit$ or stay$ or admission$ or admitted or transport$)).ti,ab,ot,hw. 196151

15 ((GP or general practitioner$ or doctor$ or inpatient$ or in-patient$ or clinician$ or specialist$ or physician$ or clinic or clinics or outpatient$ or out-patient$ or surger$) adj2 (appointment$ or attention$ or trip or trips or visit$)).ti,ab,ot,kw,kf. 44335

16 ((hospital$ or inpatient$ or in-patient$) adj2 (stay$ or admission$ or episode$)).ti,ab,ot,kw,kf. 169849

17 ((length$ or hospital$ or inpatient$ or in-patient$ or ICU or HDU or "intensive care" or "high dependancy") adj2 stay$).ti,ab,ot,kw,kf. 178121

18 ((outpatient$ or outpatient$ or inpatient$ or in-patient$ or ambulatory or emergenc$) adj2 care).ti,ab,ot,kw,kf. 65267

19 (cost$ adj2 (burden$ or estimat$ or variabl$ or hospital$ or control$)).ti,ab,ot,kw,kf. 48112

20 (district nurs$ or health visitor$ or health visiting or community nurs$ or community care or community visit$ or home visit$).ti,ab,ot,kf,kw. 23919

21 (in-patient stay$ or inpatient stay$).ti,ab,ot,hw. 3360

22 ((ambulatory or ambulance or hospital or A&E or emergency) adj2 (attention$ or trip or trips or visit$ or stay$ or admission$ or admitted or transport$)).ti,ab,ot,kw,kf. 196790

23 or/1-22 872226

24 economics/ or economics, dental/ or exp "economics, hospital"/ or economics, medical/ or economics, nursing/ or socioeconomics/ or exp fee/ or cost/ 137950

25 "Costs and Cost Analysis"/ 50808

26 Economics, Pharmaceutical/ 3076

27 (econom$ or cost or costs or costly or costing or price or prices or pricing or pharmacoeconomic$).ti,ab. 977773

28 (expenditure$ not energy).ti,ab. 34131

29 (value adj2 money).ti,ab. 1999

30 budget$.ti,ab. 33156

31 or/24-30 1084821

32 ((metabolic adj cost) or (energy adj cost) or (energy adj expenditure)).ti,ab. 32118

33 31 not 32 1077804

34 "Quality of Life"/ 248328

35 (sf36 or sf 36 or sf-36 or short form 36 or shortform 36 or sf thirtysix or sf thirty six or shortform thirtysix or shortform thirty six or short form thirty six or short form thirtysix or short form thirty six).ti,ab,ot. 28702

36 (sf6 or sf 6 or sf-6 or short form 6 or shortform 6 or sf six or sfsix or shortform six or short form six).ti,ab,ot. 2443

37 (sf12 or sf 12 or sf-12 or short form 12 or shortform 12 or sf twelve or sftwelve or shortform twelve or short form twelve).ti,ab,ot. 6938

38 (sf6D or sf 6D or sf-6D or short form 6D or shortform 6D or sf six D or sfsixD or shortform six D or short form six D).ti,ab,ot. 939

39 (sf20 or sf 20 or sf-20 or short form 20 or shortform 20 or sf twenty or sftwenty or shortform twenty or short form twenty).ti,ab,ot. 434

40 (sf8 or sf 8 or sf-8 or short form 8 or shortform 8 or sf eight or sfeight or shortform eight or short form eight).ti,ab,ot. 692

41 "health related quality of life".ti,ab,ot. 52273

42 (Quality adjusted life or Quality-adjusted-life).ti,ab,ot. 15527

43 (eq-5d or eq5d or eq-5 or eq5 or euro qual or euroqual or euro qual5d or euroqual5d or euro qol or euroqol or euro qol5d or euroqol5d or euro quol or euroquol or euro quol5d or euroquol5d or eur qol or eurqol or eur qol5d or eur qol5d or eur?qul or eur?qul5d or euro$ quality of life or european qol).ti,ab,kf. 15075

44 (euro$ adj3 (5 d or 5d or 5 dimension$ or 5dimension$ or 5 domain$ or 5domain$)).ti,ab,kf. 5260

45 (hql or hrql or hqol or h qol or hrqol or hr qol or hye or hyes).ti,ab,ot. 25097

46 ((cough or coughing) adj3 frequen$).ti,ab,ot,kf,kw. 1255

47 health$ year$ equivalent$.ti,ab,ot. 40

48 (hui or hui1 or hui2 or hui3 or hui4 or hui-4 or hui-1 or hui-2 or hui-3).ti,ab,ot. 1815

49 (quality time or qwb or quality of well being or "quality of wellbeing" or "index of wellbeing" or "index of well being").ti,ab,ot,hw. 1065

50 (Disability adjusted life or Disability-adjusted life or health adjusted life or health-adjusted life or "years of healthy life" or healthy years equivalent or "years of potential life lost" or "years of health life lost").ti,ab,ot. 5254

51 (qald$ or qale$ or qtime$).ti,ab,kf. 212

52 (timetradeoff or time tradeoff or time trade-off or time trade off or TTO or Standard gamble$ or "willingness to pay").ti,ab,ot. 9890

53 15d.ti,ab,ot. 1880

54 (HSUV$ or health state$ value$ or health state$ preference$ or HSPV$).ti,ab,ot. 466

55 (utilit$ adj3 ("quality of life" or valu$ or scor$ or measur$ or health or life or estimat$ or elicit$ or disease$ or cost$ or disease$ or mean or gain or gains or index$)).ti,ab,ot. 20895

56 (utilities or disutili$).ti,ab,ot. 8839

57 (illness state$1 or health state$1).ti,ab,kf. 7671

58 (multiattribute$ or multi attribute$).ti,ab,kf. 1135

59 Cost-Benefit Analysis/ and (cost-effectiveness ratio$ and (perspective$ or life expectanc$)).ti,ab,kf. 4817

60 ((quality of life or qol) adj (score$1 or measure$1)).ti,ab,kf. 19870

61 "surveys and questionnaires"/ or exp health care surveys/ or exp health surveys/ or patient health questionnaire/ 1131111

62 health status indicators/ or chronic disease indicators/ or sickness impact profile/ 31051

63 "Severity of Illness Index"/ 268371

64 health surveys/ or exp health status indicators/ 399613

65 interview/ 30378

66 Interviews as Topic/ 66803

67 (survey or surveys or questionnaire$ or interview or interviews or "focus group$").ti,ab,ot,kf,kw. 1465272

68 (PRO or PROs or patient reported outcome$).ti,ab,ot,kf,kw. 271837

69 (CRQ or PICQol or "CAP-sym" or "MRF26" or "MRF-26" or "AQ30" or "AQ-30" or "AQ20" or "AQ-20" or BPQ or CRQ or PFSDQ$ or SGRQ or LCQ or K-BILD or BILD).ti,ab,ot,kf,kw. 4792

70 or/34-69 2534508

71 or/23,33,70 3914059

72 exp Animals/ not (exp Animals/ and Humans/) 5038644

73 71 not 72 3709072

74 73 not (comment or editorial or letter).pt. 3600469

75 Cough/ 17932

76 cough$.ti,ab,ot,kf,kw,hw. 70944

77 or/75-76 70944

78 exp Lung Diseases, Interstitial/ or exp Agricultural Workers' Diseases/ or Asthma, Occupational/ or Bird Fancier's Lung/ or exp Pneumoconiosis/ 88361

79 ((Interstitial or diffuse parenchymal) adj3 lung adj2 (disorder$ or disease$)).ti,ab,ot,kf,kw. 14650

80 (ILD or ILDs or DPLD or IPD or IIP or IPF or DIP or AIP or NSIP or RBILD or RB-ILD or COP or BOOP or CWP).ti,ab,ot,kf,kw. 42785

81 pulmonary fibrosis.ti,ab,ot,kf,kw. 22715

82 ((Interstitial or diffuse parenchymal) adj3 pulmonary adj2 (disease$ or disorder$)).ti,ab,ot,kf,kw. 947

83 (Interstitial adj3 (Pneumonia$ or Pneumonitides or Pneumonitis or pneumopath$ or pneumocystic)).ti,ab,ot,kf,kw. 12177

84 (chronic fibrous pneumonia$ or fibroid phthisis or phthisis fibroidea or pneumonia chronica fibrosa or pneumonia fibrosa chronica or pneumonia interstitialis or pneumonitis acuta).ti,ab,ot,kf,kw. 2

85 ((carrington$ or Loeffler$ or Loffler$ or caplan$ or Hamman-Rich$ or Antisynthetase or Anti-synthetase) adj2 (disease$ or disorder$ or syndrome$)).ti,ab,ot,kf,kw. 1644

86 (eosinophil$ adj3 (pleurisy or pleuritis or lung or lungs or loeffler$ or Loffler$ or pneumonia$ or infiltrate or pulmonary)).ti,ab,ot,kf,kw. 5900

87 ((wegener$ or polyangiitis or necrotising or respiratory or necrotizing or pneumogenic) adj2 granulomatosis).ti,ab,ot,kf,kw. 8814

88 (wegener$ adj2 (syndrome$ or disease$ or disorder$ or morbus or granuloma$ or churg or klinger)).ti,ab,ot,kf,kw. 6328

89 (lung coniosis or pneumoconiosis or pneumoconiotic or pneumokoniosis or pneumonoconiosis or pneumonokoniosis or silicoarthritis).ti,ab,ot,kf,kw. 5469

90 ((Cryptogenic organizing or Cryptogenic organising or bronchiolitis obliterans) adj3 pneumonia$).ti,ab,ot,kf,kw. 1397

91 ((thatched roof or maple bark or detergent worker$ or welder$) adj2 (disease$ or disorder$ or syndrome$)).ti,ab,ot,kf,kw. 52

92 ((coal miner$ or coalworker$ or worker$ or black or aluminium or welder$ or iron miner$ or iron-miner$ or labrador or bird fancier$ or indium or bird breeder$ or pigeon keeper$ or cheese washer$ or compost or farmer$ or hot tub or humidifier$ or Japanese summer house or maple bark or miller$ or peat moss$ or snuff or trombone Player$ or Brass Player$ or Wine-grower$ or woodworker$) adj2 lung$).ti,ab,ot,kf,kw. 1683

93 (Asbestosis or silicosis or anthracosis or Aluminosis or siderosis or Berylliosis or Byssinosis or Chalicosis or Silicosiderosis or Stannosis or Talcosis or Baritosis or bagassosis or Lycoperdonosis or Sequoiosis or Suberosis).ti,ab,ot,kf,kw. 12154

94 ((Bauxite or beryllium) adj2 (disease$ or disorder$ or poisoning or syndrome$ or fibrosis)).ti,ab,ot,kf,kw. 620

95 (pulmonary adj2 (haemosiderosis or haemo-siderosis)).ti,ab,ot,kf,kw. 189

96 ((Hypersensitivity or hyper-sensitivity) adj2 pneumonitis).ti,ab,ot,kf,kw. 2809

97 extrinsic allergic alveolitis.ti,ab,ot,kf,kw. 609

98 ((Cephalosporium or Isocyanate or Trimellitic anhydride or TMA or Familial or Domestic or Japanese summer or Metalwork$ or Metal-work$ or Mollusc or shell or Sauna$ or worker$ or Streptomyces or Tap water) adj2 HP).ti,ab,ot,kf,kw. 46

99 Fibrosing alveolitis.ti,ab,ot,kf,kw. 884

100 ((alveolar or lung$ or interstitial or pulmonary) adj3 fibrosis).ti,ab,ot,kf,kw. 40977

101 or/78-100 162155

102 74 and 77 and 101 577

**103 limit 102 to yr="2010 -Current" 333**

Humanistic burden terms based on:

Clayton S, Bambra C, Gosling R, Povall S, Misso K, Whitehead M. Assembling the evidence jigsaw: insights from a systematic review of UK studies of individual-focused return to work initiatives for disabled and long-term ill people [Internet]. BMC Public Health 2011 [cited 23.3.11];11:170.

Cost-effectiveness filter based on:

Centre for Reviews and Dissemination. Search strategies: NHS EED EMBASE using OvidSP (economics filter) [Internet]. York: Centre for Reviews and Dissemination; 2014 [accessed 2.6.14]. Available from:

http://www.crd.york.ac.uk/crdweb/searchstrategies.asp#nhseedembase

HRQoL free-text terms based on:

Figure 4: Common free-text terms for electronic database searching for HSUVs in Papaioannou D, Brazier JE, Paisley S. NICE DSU Technical Support Document 9: the identification, review and synthesis of health state utility values from the literature (Internet), 2011 (accessed: 18.8.11) Available from: http://www.nicedsu.org.uk

Arber M, Garcia S, Veale T, Edwards M, Shaw A, Glanville JM. Performance of Ovid Medline search filters to identify health state utility studies. Int J Technol Assess Health Care 2017;33(4):472-80.

**Medline Daily Update & Epubs Ahead-of-Print (Ovid): 2010-2022/08/31**

**Searched 1.9.22**

1 Efficiency/ 23

2 Absenteeism/ 5

3 Caregiver Burden/ 4

4 Caregivers/ or Stress, Psychological/ or Financial Stress/ or exp Occupational Stress/ 277

5 ((human$ or social$ or societ$ or work$ or employe$ or business$ or communit$ or famil$ or carer$ or caregiver$) adj3 (burden$ or consequenc$ or impact$ or problem$ or productivity or sickness or impairment$)).ti,ab,ot,hw. 3668

6 ((long standing or longstanding or long term or longterm or permanent or employee$) adj2 (absence$ or absent$ or ill$ or sick$ or disab$)).ti,ab,ot,hw. 267

7 (llsi or ((emergenc$ or domestic$ or famil$ or carer$ or caregiver$) adj3 leave$)).ti,ab,ot. 29

8 ((cost or costs or burden) adj2 (illness$ or disease$ or sickness$)).ti,ab,ot,kw,kf. 1008

9 ((allowance or status or long-term or pension$ or benefit$) adj2 (disab$ or incapacit$)).ti,ab,ot,hw. 349

10 ((unable or inability or incapacit$ or incapab$) adj3 work).ti,ab,ot,hw. 38

11 ((resource$ or fund$) adj2 (use$ or utili?ation)).ti,ab,ot,hw. 959

12 ((health or healthcare) adj2 (resource$ or fund$)).ti,ab,ot,hw. 919

13 ("length of stay" or "duration of stay" or "extended stay" or "prolonged stay" or "stay days").ti,ab,ot,kw,kf. 2298

14 ((ambulatory or ambulance or hospital or A&E or emergency) adj2 (attention$ or trip or trips or visit$ or stay$ or admission$ or admitted or transport$)).ti,ab,ot,hw. 4474

15 ((GP or general practitioner$ or doctor$ or inpatient$ or in-patient$ or clinician$ or specialist$ or physician$ or clinic or clinics or outpatient$ or out-patient$ or surger$) adj2 (appointment$ or attention$ or trip or trips or visit$)).ti,ab,ot,kw,kf. 866

16 ((hospital$ or inpatient$ or in-patient$) adj2 (stay$ or admission$ or episode$)).ti,ab,ot,kw,kf. 3975

17 ((length$ or hospital$ or inpatient$ or in-patient$ or ICU or HDU or "intensive care" or "high dependancy") adj2 stay$).ti,ab,ot,kw,kf. 4650

18 ((outpatient$ or outpatient$ or inpatient$ or in-patient$ or ambulatory or emergenc$) adj2 care).ti,ab,ot,kw,kf. 1552

19 (cost$ adj2 (burden$ or estimat$ or variabl$ or hospital$ or control$)).ti,ab,ot,kw,kf. 1034

20 (district nurs$ or health visitor$ or health visiting or community nurs$ or community care or community visit$ or home visit$).ti,ab,ot,kf,kw. 578

21 (in-patient stay$ or inpatient stay$).ti,ab,ot,hw. 161

22 ((ambulatory or ambulance or hospital or A&E or emergency) adj2 (attention$ or trip or trips or visit$ or stay$ or admission$ or admitted or transport$)).ti,ab,ot,kw,kf. 4531

23 or/1-22 16339

24 economics/ or economics, dental/ or exp "economics, hospital"/ or economics, medical/ or economics, nursing/ or socioeconomics/ or exp fee/ or cost/ 17

25 "Costs and Cost Analysis"/ 13

26 Economics, Pharmaceutical/ 3

27 (econom$ or cost or costs or costly or costing or price or prices or pricing or pharmacoeconomic$).ti,ab. 20467

28 (expenditure$ not energy).ti,ab. 775

29 (value adj2 money).ti,ab. 59

30 budget$.ti,ab. 574

31 or/24-30 21134

32 ((metabolic adj cost) or (energy adj cost) or (energy adj expenditure)).ti,ab. 365

33 31 not 32 21063

34 "Quality of Life"/ 620

35 (sf36 or sf 36 or sf-36 or short form 36 or shortform 36 or sf thirtysix or sf thirty six or shortform thirtysix or shortform thirty six or short form thirty six or short form thirtysix or short form thirty six).ti,ab,ot. 447

36 (sf6 or sf 6 or sf-6 or short form 6 or shortform 6 or sf six or sfsix or shortform six or short form six).ti,ab,ot. 46

37 (sf12 or sf 12 or sf-12 or short form 12 or shortform 12 or sf twelve or sftwelve or shortform twelve or short form twelve).ti,ab,ot. 178

38 (sf6D or sf 6D or sf-6D or short form 6D or shortform 6D or sf six D or sfsixD or shortform six D or short form six D).ti,ab,ot. 19

39 (sf20 or sf 20 or sf-20 or short form 20 or shortform 20 or sf twenty or sftwenty or shortform twenty or short form twenty).ti,ab,ot. 3

40 (sf8 or sf 8 or sf-8 or short form 8 or shortform 8 or sf eight or sfeight or shortform eight or short form eight).ti,ab,ot. 14

41 "health related quality of life".ti,ab,ot. 1350

42 (Quality adjusted life or Quality-adjusted-life).ti,ab,ot. 451

43 (eq-5d or eq5d or eq-5 or eq5 or euro qual or euroqual or euro qual5d or euroqual5d or euro qol or euroqol or euro qol5d or euroqol5d or euro quol or euroquol or euro quol5d or euroquol5d or eur qol or eurqol or eur qol5d or eur qol5d or eur?qul or eur?qul5d or euro$ quality of life or european qol).ti,ab,kf. 509

44 (euro$ adj3 (5 d or 5d or 5 dimension$ or 5dimension$ or 5 domain$ or 5domain$)).ti,ab,kf. 171

45 (hql or hrql or hqol or h qol or hrqol or hr qol or hye or hyes).ti,ab,ot. 641

46 ((cough or coughing) adj3 frequen$).ti,ab,ot,kf,kw. 27

47 health$ year$ equivalent$.ti,ab,ot. 0

48 (hui or hui1 or hui2 or hui3 or hui4 or hui-4 or hui-1 or hui-2 or hui-3).ti,ab,ot. 31

49 (quality time or qwb or quality of well being or "quality of wellbeing" or "index of wellbeing" or "index of well being").ti,ab,ot,hw. 23

50 (Disability adjusted life or Disability-adjusted life or health adjusted life or health-adjusted life or "years of healthy life" or healthy years equivalent or "years of potential life lost" or "years of health life lost").ti,ab,ot. 126

51 (qald$ or qale$ or qtime$).ti,ab,kf. 4

52 (timetradeoff or time tradeoff or time trade-off or time trade off or TTO or Standard gamble$ or "willingness to pay").ti,ab,ot. 268

53 15d.ti,ab,ot. 15

54 (HSUV$ or health state$ value$ or health state$ preference$ or HSPV$).ti,ab,ot. 11

55 (utilit$ adj3 ("quality of life" or valu$ or scor$ or measur$ or health or life or estimat$ or elicit$ or disease$ or cost$ or disease$ or mean or gain or gains or index$)).ti,ab,ot. 519

56 (utilities or disutili$).ti,ab,ot. 187

57 (illness state$1 or health state$1).ti,ab,kf. 144

58 (multiattribute$ or multi attribute$).ti,ab,kf. 46

59 Cost-Benefit Analysis/ and (cost-effectiveness ratio$ and (perspective$ or life expectanc$)).ti,ab,kf. 14

60 ((quality of life or qol) adj (score$1 or measure$1)).ti,ab,kf. 451

61 "surveys and questionnaires"/ or exp health care surveys/ or exp health surveys/ or patient health questionnaire/ 936

62 health status indicators/ or chronic disease indicators/ or sickness impact profile/ 0

63 "Severity of Illness Index"/ 68

64 health surveys/ or exp health status indicators/ 114

65 interview/ 2

66 Interviews as Topic/ 0

67 (survey or surveys or questionnaire$ or interview or interviews or "focus group$").ti,ab,ot,kf,kw. 32587

68 (PRO or PROs or patient reported outcome$).ti,ab,ot,kf,kw. 5140

69 (CRQ or PICQol or "CAP-sym" or "MRF26" or "MRF-26" or "AQ30" or "AQ-30" or "AQ20" or "AQ-20" or BPQ or CRQ or PFSDQ$ or SGRQ or LCQ or K-BILD or BILD).ti,ab,ot,kf,kw. 85

70 or/34-69 39451

71 or/23,33,70 66651

72 exp Animals/ not (exp Animals/ and Humans/) 2451

73 71 not 72 66404

74 73 not (comment or editorial or letter).pt. 65900

75 Cough/ 14

76 cough$.ti,ab,ot,kf,kw,hw. 904

77 or/75-76 904

78 exp Lung Diseases, Interstitial/ or exp Agricultural Workers' Diseases/ or Asthma, Occupational/ or Bird Fancier's Lung/ or exp Pneumoconiosis/ 67

79 ((Interstitial or diffuse parenchymal) adj3 lung adj2 (disorder$ or disease$)).ti,ab,ot,kf,kw. 380

80 (ILD or ILDs or DPLD or IPD or IIP or IPF or DIP or AIP or NSIP or RBILD or RB-ILD or COP or BOOP or CWP).ti,ab,ot,kf,kw. 795

81 pulmonary fibrosis.ti,ab,ot,kf,kw. 352

82 ((Interstitial or diffuse parenchymal) adj3 pulmonary adj2 (disease$ or disorder$)).ti,ab,ot,kf,kw. 13

83 (Interstitial adj3 (Pneumonia$ or Pneumonitides or Pneumonitis or pneumopath$ or pneumocystic)).ti,ab,ot,kf,kw. 141

84 (chronic fibrous pneumonia$ or fibroid phthisis or phthisis fibroidea or pneumonia chronica fibrosa or pneumonia fibrosa chronica or pneumonia interstitialis or pneumonitis acuta).ti,ab,ot,kf,kw. 0

85 ((carrington$ or Loeffler$ or Loffler$ or caplan$ or Hamman-Rich$ or Antisynthetase or Anti-synthetase) adj2 (disease$ or disorder$ or syndrome$)).ti,ab,ot,kf,kw. 21

86 (eosinophil$ adj3 (pleurisy or pleuritis or lung or lungs or loeffler$ or Loffler$ or pneumonia$ or infiltrate or pulmonary)).ti,ab,ot,kf,kw. 56

87 ((wegener$ or polyangiitis or necrotising or respiratory or necrotizing or pneumogenic) adj2 granulomatosis).ti,ab,ot,kf,kw. 130

88 (wegener$ adj2 (syndrome$ or disease$ or disorder$ or morbus or granuloma$ or churg or klinger)).ti,ab,ot,kf,kw. 22

89 (lung coniosis or pneumoconiosis or pneumoconiotic or pneumokoniosis or pneumonoconiosis or pneumonokoniosis or silicoarthritis).ti,ab,ot,kf,kw. 31

90 ((Cryptogenic organizing or Cryptogenic organising or bronchiolitis obliterans) adj3 pneumonia$).ti,ab,ot,kf,kw. 5

91 ((thatched roof or maple bark or detergent worker$ or welder$) adj2 (disease$ or disorder$ or syndrome$)).ti,ab,ot,kf,kw. 0

92 ((coal miner$ or coalworker$ or worker$ or black or aluminium or welder$ or iron miner$ or iron-miner$ or labrador or bird fancier$ or indium or bird breeder$ or pigeon keeper$ or cheese washer$ or compost or farmer$ or hot tub or humidifier$ or Japanese summer house or maple bark or miller$ or peat moss$ or snuff or trombone Player$ or Brass Player$ or Wine-grower$ or woodworker$) adj2 lung$).ti,ab,ot,kf,kw. 16

93 (Asbestosis or silicosis or anthracosis or Aluminosis or siderosis or Berylliosis or Byssinosis or Chalicosis or Silicosiderosis or Stannosis or Talcosis or Baritosis or bagassosis or Lycoperdonosis or Sequoiosis or Suberosis).ti,ab,ot,kf,kw. 79

94 ((Bauxite or beryllium) adj2 (disease$ or disorder$ or poisoning or syndrome$ or fibrosis)).ti,ab,ot,kf,kw. 6

95 (pulmonary adj2 (haemosiderosis or haemo-siderosis)).ti,ab,ot,kf,kw. 0

96 ((Hypersensitivity or hyper-sensitivity) adj2 pneumonitis).ti,ab,ot,kf,kw. 39

97 extrinsic allergic alveolitis.ti,ab,ot,kf,kw. 6

98 ((Cephalosporium or Isocyanate or Trimellitic anhydride or TMA or Familial or Domestic or Japanese summer or Metalwork$ or Metal-work$ or Mollusc or shell or Sauna$ or worker$ or Streptomyces or Tap water) adj2 HP).ti,ab,ot,kf,kw. 1

99 Fibrosing alveolitis.ti,ab,ot,kf,kw. 1

100 ((alveolar or lung$ or interstitial or pulmonary) adj3 fibrosis).ti,ab,ot,kf,kw. 542

101 or/78-100 1700

102 74 and 77 and 101 12

**103 limit 102 to yr="2010 -Current" 11**

Humanistic burden terms based on:

Clayton S, Bambra C, Gosling R, Povall S, Misso K, Whitehead M. Assembling the evidence jigsaw: insights from a systematic review of UK studies of individual-focused return to work initiatives for disabled and long-term ill people [Internet]. BMC Public Health 2011 [cited 23.3.11];11:170.

Cost-effectiveness filter based on:

Centre for Reviews and Dissemination. Search strategies: NHS EED EMBASE using OvidSP (economics filter) [Internet]. York: Centre for Reviews and Dissemination; 2014 [accessed 2.6.14]. Available from:

http://www.crd.york.ac.uk/crdweb/searchstrategies.asp#nhseedembase

HRQoL free-text terms based on:

Figure 4: Common free-text terms for electronic database searching for HSUVs in Papaioannou D, Brazier JE, Paisley S. NICE DSU Technical Support Document 9: the identification, review and synthesis of health state utility values from the literature (Internet), 2011 (accessed: 18.8.11) Available from: http://www.nicedsu.org.uk

Arber M, Garcia S, Veale T, Edwards M, Shaw A, Glanville JM. Performance of Ovid Medline search filters to identify health state utility studies. Int J Technol Assess Health Care 2017;33(4):472-80.

**Cochrane Database of Systematic Reviews (CDSR) (Wiley): Issue 8/12 August 2022**

**Searched 1.9.22**

**Limited 2010-2022/09/01**

#1 MeSH descriptor: [Lung Diseases, Interstitial] explode all trees 1292

#2 MeSH descriptor: [Agricultural Workers' Diseases] explode all trees 44

#3 MeSH descriptor: [undefined] explode all trees 0

#4 MeSH descriptor: [Bird Fancier's Lung] this term only 3

#5 MeSH descriptor: [undefined] explode all trees 0

#6 ((Interstitial OR "diffuse parenchymal") NEAR/3 lung NEAR/2 (disorder* OR disease*)):ti,ab 1217

#7 (ILD OR ILDs OR DPLD OR IPD OR IIP OR IPF OR DIP OR AIP OR NSIP OR RBILD OR RB-ILD OR COP OR BOOP OR CWP):ti,ab 4103

#8 "pulmonary fibrosis":ti,ab 1621

#9 ((Interstitial OR "diffuse parenchymal") NEAR/3 pulmonary NEAR/2 (disease* OR disorder*)):ti,ab 118

#10 (Interstitial NEAR/3 (Pneumonia* OR Pneumonitides OR Pneumonitis OR pneumopath* OR pneumocystic)):ti,ab 471

#11 ("chronic fibrous pneumonia*" OR "fibroid phthisis" OR "phthisis fibroidea" OR "pneumonia chronica fibrosa" OR "pneumonia fibrosa chronica" OR "pneumonia interstitialis" OR "pneumonitis acuta"):ti,ab 0

#12 ((carrington* OR Loeffler* OR Loffler* OR caplan* OR Hamman-Rich* OR Antisynthetase OR "Anti-synthetase") NEAR/2 (disease* OR disorder* OR syndrome*)):ti,ab 17

#13 (eosinophil* NEAR/3 (pleurisy OR pleuritis OR lung OR lungs OR loeffler* OR Loffler* OR pneumonia* OR infiltrate OR pulmonary)):ti,ab 130

#14 ((wegener* OR polyangiitis OR necrotising OR respiratory OR necrotizing OR pneumogenic) NEAR/2 granulomatosis):ti,ab 328

#15 (wegener* NEAR/2 (syndrome* OR disease* OR disorder* OR morbus OR granuloma* OR churg OR klinger)):ti,ab 134

#16 ("lung coniosis" OR pneumoconiosis OR pneumoconiotic OR pneumokoniosis OR pneumonoconiosis OR pneumonokoniosis OR silicoarthritis):ti,ab 76

#17 (("Cryptogenic organizing" OR "Cryptogenic organizing" OR "bronchiolitis obliterans") NEAR/3 pneumonia*):ti,ab 15

#18 (("thatched roof" OR "maple bark" OR "detergent worker*" OR welder*) NEAR/2 (disease* OR disorder* OR syndrome*)):ti,ab 0

#19 (("coal miner*" OR coalworker* OR worker* OR black OR aluminium OR welder* OR "iron miner*" OR "iron-miner*" OR labrador OR "bird fancer*" OR indium OR "bird breeder*" OR "pigeon keeper*" OR "cheese washer*" OR compost OR farmer* OR "hot tub" OR humidifier* OR "Japanese summer house" OR "maple bark" OR miller* OR "peat moss*" OR snuff OR "trombone Player*" OR "Brass Player*" OR "Wine-grower*" OR woodworker*) NEAR/2 lung*):ti,ab 19

#20 (Asbestosis OR silicosis OR anthracosis OR Aluminosis OR siderosis OR Berylliosis OR Byssinosis OR Chalicosis OR Silicosiderosis OR Stannosis OR Talcosis OR Baritosis OR bagassosis OR Lycoperdonosis OR Sequoiosis OR Suberosis):ti,ab 144

#21 ((Bauxite OR beryllium) NEAR/2 (disease* OR disorder* OR poisoning OR syndrome* OR fibrosis)):ti,ab 10

#22 (pulmonary NEAR/2 (haemosiderosis OR haemo-siderosis)):ti,ab 0

#23 ((Hypersensitivity OR "hyper-sensitivity") NEAR/2 pneumonitis):ti,ab 5301

#24 "extrinsic allergic alveolitis":ti,ab 1

#25 ((Cephalosporium OR Isocyanate OR "Trimellitic anhydride" OR TMA OR Familial OR Domestic OR "Japanese summer "OR Metalwork* OR "Metal-work*" OR Mollusc OR shell OR Sauna* OR worker* OR Streptomyces OR "Tap water") NEAR/2 HP):ti,ab 0

#26 "Fibrosing alveolitis":ti,ab 19

#27 ((alveolar OR lung* OR interstitial OR pulmonary) NEAR/3 fibrosis):ti,ab 2412

#28 #1 OR #2 OR #3 OR #4 OR #5 OR #6 OR #7 OR #8 OR #9 OR #10 OR #11 OR #12 OR #13 OR #14 OR #15 OR #16 OR #17 OR #18 OR #19 OR #20 OR #21 OR #22 OR #23 OR #24 OR #25 OR #26 OR #27 1937422

#29 MeSH descriptor: [Cough] this term only 1463

#30 cough*:ti,ab,kw 16672

#31 #29 OR #30 16672

#32 #28 AND #31 with Cochrane Library publication date Between Jan 2010 and Aug 2022, in Cochrane Reviews, Cochrane Protocols 264

#123 nct*:au 231950

**#124 #122 not #123 3300**

**Protocols = 4**

**Reviews = 260**

**PubMed (NLM) (Internet): 2010-2022/09/01**

**Searched 1.9.22**

**31 #27 NOT #30 877 (limited 2010-2022/09/01)**

30 #29 NOT (#29 AND #28) 1,250,812

29 rat[tiab] OR rats[tiab] OR mouse[tiab] OR mice[tiab] OR murine[tiab] OR rodent[tiab] OR rodents[tiab] OR hamster[tiab] OR hamsters[tiab] OR pig[tiab] OR pigs[tiab] OR porcine[tiab] OR rabbit[tiab] OR rabbits[tiab] OR animal[tiab] OR animals[tiab] OR dogs[tiab] OR dog[tiab] OR cats[tiab] OR cow[tiab] OR bovine[tiab] OR sheep[tiab] OR ovine[tiab] OR monkey[tiab] OR monkeys[tiab] 1,687,791

28 Human[tiab] OR humans[tiab] 1,506,847

27 #6 AND #26 912

26 #7 OR #8 OR #9 OR #10 OR #11 OR #12 OR #14 OR #15 OR #16 OR #17 OR #19 OR #20 OR #21 OR #22 OR #23 OR #24 OR #25 342,747

25 "alveolar fibrosis"[Title/Abstract] OR "lung fibrosis"[Title/Abstract] OR "lungs fibrosis"[Title/Abstract] OR "interstitial fibrosis"[Title/Abstract] OR "pulmonary fibrosis"[Title/Abstract] 21,668

24 "Cephalosporium HP"[Title/Abstract] OR "Isocyanate HP"[Title/Abstract] OR "Trimellitic anhydride HP"[Title/Abstract] OR "TMA HP"[Title/Abstract] OR "Familial HP"[Title/Abstract] OR "Domestic HP"[Title/Abstract] OR "Japanese summer HP"[Title/Abstract] OR "Metalwork HP"[Title/Abstract] OR "Metal-worker HP"[Title/Abstract] OR "Metalworker HP"[Title/Abstract] OR "Metal-workers HP"[Title/Abstract] OR "Metal-work HP"[Title/Abstract] OR "Mollusc HP"[Title/Abstract] OR "shell HP"[Title/Abstract] OR "Sauna HP"[Title/Abstract] OR "Saunas HP"[Title/Abstract] OR "worker HP"[Title/Abstract] OR "workers HP"[Title/Abstract] OR "Streptomyces HP"[Title/Abstract] OR "Tap water HP"[Title/Abstract] OR "Fibrosing alveolitis"[Title/Abstract] 52

23 "Hypersensitivity pneumonitis"[Title/Abstract] OR "hyper-sensitivity pneumonitis"[Title/Abstract] OR "extrinsic allergic alveolitis"[Title/Abstract] 1,330

22 "pulmonary haemosiderosis"[Title/Abstract] OR "pulmonary haemo-siderosis"[Title/Abstract] OR "pulmonary hemosiderosis"[Title/Abstract] OR "pulmonary hemo-siderosis"[Title/Abstract] 177

21 (Bauxite[Title/Abstract] OR beryllium[Title/Abstract]) AND (syndrome[Title/Abstract] OR disease[Title/Abstract] OR disorder[Title/Abstract] OR syndromes[Title/Abstract] OR diseases[Title/Abstract] OR disorders[Title/Abstract] OR poisoning[Title/Abstract] OR fibrosis[Title/Abstract]) 211

20 Asbestosis[Title/Abstract] OR silicosis[Title/Abstract] OR anthracosis[Title/Abstract] OR Aluminosis[Title/Abstract] OR siderosis[Title/Abstract] OR Berylliosis[Title/Abstract] OR Byssinosis[Title/Abstract] OR Chalicosis[Title/Abstract] OR Silicosiderosis[Title/Abstract] OR Stannosis[Title/Abstract] OR Talcosis[Title/Abstract] OR Baritosis[Title/Abstract] OR bagassosis[Title/Abstract] OR Lycoperdonosis[Title/Abstract] OR Sequoiosis[Title/Abstract] OR Suberosis[Title/Abstract] 3,068

19 ("coal miner"[Title/Abstract] OR coalworker[Title/Abstract] OR worker[Title/Abstract] OR "coal miners"[Title/Abstract] OR coalworkers[Title/Abstract] OR workers[Title/Abstract] OR black[Title/Abstract] OR aluminium[Title/Abstract] OR "iron miner"[Title/Abstract] OR "iron-miner"[Title/Abstract] OR "iron miners"[Title/Abstract] OR "iron-miners"[Title/Abstract] OR miner[Title/Abstract] OR miners[Title/Abstract] OR labrador[Title/Abstract] OR "bird fancier"[Title/Abstract] OR "bird fanciers"[Title/Abstract] OR indium[Title/Abstract] OR "bird breeder"[Title/Abstract] OR "pigeon keeper"[Title/Abstract] OR "cheese washer"[Title/Abstract] OR "bird breeders"[Title/Abstract] OR "pigeon keepers"[Title/Abstract] OR "cheese washers"[Title/Abstract] OR compost[Title/Abstract] OR farmer[Title/Abstract] OR farmers[Title/Abstract] OR "hot tub"[Title/Abstract] OR humidifier[Title/Abstract] OR humidifiers[Title/Abstract] OR "Japanese summer house"[Title/Abstract] OR "maple bark"[Title/Abstract] OR miller[Title/Abstract] OR "peat moss"[Title/Abstract] OR millers[Title/Abstract] OR snuff[Title/Abstract] OR "trombone Player"[Title/Abstract] OR "Brass Player"[Title/Abstract] OR "trombone Players"[Title/Abstract] OR "Brass Players"[Title/Abstract] OR "Wine-grower"[Title/Abstract] OR woodworker[Title/Abstract] OR woodworkers[Title/Abstract]) OR (lung[Title/Abstract] AND lungs[Title/Abstract]) 278,497

18 ("thatched roof"[Title/Abstract] OR "maple bark"[Title/Abstract] OR "detergent worker"[Title/Abstract] OR welder[Title/Abstract] OR "detergent workers"[Title/Abstract] OR welders[Title/Abstract]) AND (syndrome[Title/Abstract] OR disease[Title/Abstract] OR disorder[Title/Abstract] OR syndromes[Title/Abstract] OR diseases[Title/Abstract] OR disorders[Title/Abstract]) 198

17 ("Cryptogenic organizing"[Title/Abstract] OR "Cryptogenic organizing"[Title/Abstract] OR "bronchiolitis obliterans"[Title/Abstract]) AND pneumonia[Title/Abstract] 586

16 "lung coniosis"[Title/Abstract] OR pneumoconiosis[Title/Abstract] OR pneumoconiotic[Title/Abstract] OR pneumokoniosis[Title/Abstract] OR pneumonoconiosis[Title/Abstract] OR pneumonokoniosis[Title/Abstract] OR silicoarthritis[Title/Abstract] 1,230

15 wegener[Title/Abstract] AND (syndrome[Title/Abstract] OR disease[Title/Abstract] OR disorder[Title/Abstract] OR syndromes[Title/Abstract] OR diseases[Title/Abstract] OR disorders[Title/Abstract] OR morbus[Title/Abstract] OR granuloma[Title/Abstract] OR churg[Title/Abstract] OR klinger[Title/Abstract]) 1,084

14 (wegener[Title/Abstract] OR polyangiitis[Title/Abstract] OR necrotising[Title/Abstract] OR respiratory[Title/Abstract] OR necrotizing[Title/Abstract] OR pneumogenic[Title/Abstract]) AND granulomatosis[Title/Abstract] 4,196

13 eosinophil[Title/Abstract] AND (pleurisy[Title/Abstract] OR pleuritis[Title/Abstract] OR lung[Title/Abstract] OR lungs[Title/Abstract] OR loeffler[Title/Abstract] OR Loffler[Title/Abstract] OR pneumonia[Title/Abstract] OR infiltrate[Title/Abstract] OR pulmonary[Title/Abstract]) 3,187

12 (carrington[Title/Abstract] OR Loeffler[Title/Abstract] OR Loffler[Title/Abstract] OR caplan[Title/Abstract] OR Hamman-Rich[Title/Abstract] OR Antisynthetase[Title/Abstract] OR "Anti-synthetase"[Title/Abstract]) AND (disease[Title/Abstract] OR disorder[Title/Abstract] OR syndrome[Title/Abstract] OR diseases[Title/Abstract] OR disorders[Title/Abstract] OR syndromes[Title/Abstract]) 928

11 ("chronic fibrous pneumonia"[Title/Abstract] OR "fibroid phthisis"[Title/Abstract] OR "phthisis fibroidea"[Title/Abstract] OR "pneumonia chronica fibrosa"[Title/Abstract] OR "pneumonia fibrosa chronica"[Title/Abstract] OR "pneumonia interstitialis"[Title/Abstract] OR "pneumonitis acuta"[Title/Abstract]) 0

10 "Interstitial Pneumonia"[Title/Abstract] OR "Interstitial Pneumonitides"[Title/Abstract] OR "Interstitial Pneumonitis"[Title/Abstract] OR "Interstitial pneumopathy"[Title/Abstract] OR "Interstitial pneumocystic"[Title/Abstract] OR "Interstitial pneumopathies"[Title/Abstract] OR "Interstitial Pneumonias"[Title/Abstract] 5,319

9 "Interstitial pulmonary"[Title/Abstract] OR "diffuse parenchymal pulmonary"[Title/Abstract] 337

8 ILD[Title/Abstract] OR ILDs[Title/Abstract] OR DPLD[Title/Abstract] OR IPD[Title/Abstract] OR IIP[Title/Abstract] OR IPF[Title/Abstract] OR DIP[Title/Abstract] OR AIP[Title/Abstract] OR NSIP[Title/Abstract] OR RBILD[Title/Abstract] OR RB-ILD[Title/Abstract] OR COP[Title/Abstract] OR BOOP[Title/Abstract] OR CWP[Title/Abstract] 30,275

7 "Interstitial lung"[Text Word] OR "diffuse parenchymal lung"[Text Word] OR "Pneumoconiosis"[Mesh] OR "Bird Fancier's Lung"[Mesh:NoExp] OR "Asthma, Occupational"[Mesh:NoExp] OR "Agricultural Workers' Diseases"[Mesh] OR "Lung Diseases, Interstitial"[Mesh] 34,175

6 #3 AND #4 8,809

5 #3 AND #4 9,803

4 pubstatusaheadofprint OR publisher[sb] OR pubmednotmedline[sb] 4,840,632

3 #1 OR #2 71,722

2 cough[Text Word] OR coughs[Text Word] OR coughing[Text Word] OR coughed[Text Word] 71,722

1 "Cough"[Mesh:NoExp] 17,930

**Europe PMC, including MedRxiv and bioRxiv preprints (Internet): 2010-2022/09/01**

**Searched 1.9.22**

<https://europepmc.org/advancesearch>

**Limited 2010-2022**

| **Search terms (limited to preprints only)**  **Simple search** | **Results** |
| --- | --- |
| (TITLE:"cough" OR TITLE:"coughs" OR TITLE:"coughed" OR TITLE:"coughing")  AND  ("cost" OR "costs" OR "economics" OR "burden" OR "caregiver" OR "caregivers" OR "isolation" OR "quality of life" OR "qol" OR "hrqol" OR "hrql" OR "survey" OR "questionnaire" OR "impact" OR "productivity" OR "employment" OR "social" OR "benefit" OR "pension" OR "costing" OR "costly" OR "price" OR "expenditure" OR "disability" OR "health status" OR "adjusted life" OR "utility" OR "utilities" OR "surveys" OR "questionnaires" OR "interview" OR "interviews")  **All fields** | 101 |
| (TITLE:"cough" OR TITLE:"coughs" OR TITLE:"coughed" OR TITLE:"coughing")  AND  "interstitial" OR "ILD" OR "ilds" OR "DPLD" OR "IPD" OR "IIP" OR "IPF" OR "DIP" OR "AIP" OR "NSIP" OR "RBILD" OR "RB-ILD" OR "COP" OR "BOOP" OR "CWP" OR "fibrosis" OR "diffuse parenchymal pulmonary" OR "chronic fibrous pneumonia" OR "fibroid phthisis" OR "phthisis fibroidea" OR "pneumonia chronica fibrosa" OR "pneumonia fibrosa chronica" OR "pneumonia interstitialis" OR "pneumonitis acuta" OR "eosinophil" OR "lung injury" OR "lung injuries"  **All fields** | 10 |
| ("cough" OR "coughs" OR "coughed" OR "coughing")  **All fields**  AND  ("wegener" OR "lung coniosis" OR "pneumoconiosis" OR "pneumoconiotic" OR "pneumokoniosis" OR "pneumonoconiosis" OR "pneumonokoniosis" OR "silicoarthritis" OR "crytogenic" OR "bronchiolitis obliterans" OR "miners lung" OR "coalminers lung" OR "pulmonary haemosiderosis" OR "pulmonary haemo-siderosis" OR "pulmonary hemosiderosis" OR "pulmonary hemo-siderosis")  **All fields** | 16 |
| (TITLE:"cough" OR TITLE:"coughs" OR TITLE:"coughed" OR TITLE:"coughing")  AND  ("Asbestosis" OR "silicosis" OR "anthracosis" OR "Aluminosis" OR "siderosis" OR "Berylliosis" OR "Byssinosis" OR "Chalicosis" OR "Silicosiderosis" OR "Stannosis" OR "Talcosis" OR "Baritosis" OR "bagassosis" OR "Lycoperdonosis" OR "Sequoiosis" OR "Suberosis" OR "Hypersensitivity pneumonitis" OR "hyper-sensitivity pneumonitis" OR "extrinsic allergic alveolitis" OR "alveolar fibrosis" OR "lung fibrosis" OR "lungs fibrosis" OR "interstitial fibrosis" OR "pulmonary fibrosis" OR "occupational lung" OR "occupational disease" OR "occupational diseases" OR "occupational disorder" OR "occupational disorders")  **All fields** | 2 |
| **Total (with duplicates)** | **129** |

**NHS Economic Evaluation Database (NHS EED) (CRD): 2010-2015/03/31**

**Searched 1.9.22**

**https://www.crd.york.ac.uk/CRDWeb/**

1 MeSH DESCRIPTOR Cough IN NHSEED 5

2 (cough*) IN NHSEED FROM 2010 TO 2015 23

**3 #1 OR #2 27**

## **Strategy for updated search: October 2023**

To capture the more recent publications, searches were updated in October 2023:

| **Database** | **Host** | **Date range** | **Date searched** | **Results** |
| --- | --- | --- | --- | --- |
| Embase | Ovid | 2010-2023/10/02 | 3.10.23 | 2088 |
| Medline & In-Process Citations | Ovid | 2010-2023/10/02 | 3.10.23 | 395 |
| Pubmed | NLM | 2010-2023/10/04 | 4.10.23 | 1041 |
| Europe PMC, includes BioRxiv & medRxiv | www | 2010-2023/10/04 | 4.10.23 | 154 |
| Cochrane Database of Systematic Reviews (CDSR) | Wiley | 2010-2023/10/03 | 3.10.23 | 8 |
| Cochrane Central Register of Controlled Trials (CENTRAL) | Wiley | 2010-2023/10/03 | 3.10.23 | 250 |
| NHS Economic Evaluation Database (NHS EED) | CRD | 2010-2015/03/31 | 1.9.22 | 27 |
| **Total retrieved** |  |  |  | **3949** |
| **Total screened** |  |  |  | **3424** |
| **Duplicates** |  |  |  | **823** |

**Embase (Ovid): 2010-2023/10/02**

**Searched 3.10.23**

1 Productivity/ 50171

2 Absenteeism/ 19960

3 Caregiver Burden/ 10757

4 Caregiver/ 114265

5 Work Disability/ 5775

6 ((human$ or social$ or societ$ or work$ or employe$ or business$ or communit$ or famil$ or carer$ or caregiver$) adj3 (burden$ or consequenc$ or impact$ or problem$ or productivity or sickness or impairment$)).ti,ab,ot,hw. 229522

7 ((long standing or longstanding or long term or longterm or permanent or employee$) adj2 (absence$ or absent$ or ill$ or sick$ or disab$)).ti,ab,ot,hw. 18666

8 (llsi or ((emergenc$ or domestic$ or famil$ or carer$ or caregiver$) adj3 leave$)).ti,ab,ot. 1448

9 ((cost or costs or burden) adj2 (illness$ or disease$ or sickness$)).ti,ab,ot,kw,kf. 70653

10 ((allowance or status or long-term or pension$ or benefit$) adj2 (disab$ or incapacit$)).ti,ab,ot,hw. 37869

11 ((unable or inability or incapacit$ or incapab$) adj3 work).ti,ab,ot,hw. 3281

12 ((resource$ or fund$) adj2 (use$ or utili?ation)).ti,ab,ot,hw. 66727

13 ((health or healthcare) adj2 (resource$ or fund$)).ti,ab,ot,hw. 60761

14 ("length of stay" or "duration of stay" or "extended stay" or "prolonged stay" or "stay days").ti,ab,ot,kw,kf. 157209

15 ((ambulatory or ambulance or hospital or A&E or emergency) adj2 (attention$ or trip or trips or visit$ or stay$ or admission$ or admitted or transport$)).ti,ab,ot,hw. 560579

16 ((GP or general practitioner$ or doctor$ or inpatient$ or in-patient$ or clinician$ or specialist$ or physician$ or clinic or clinics or outpatient$ or out-patient$ or surger$) adj2 (appointment$ or attention$ or trip or trips or visit$)).ti,ab,ot,kw,kf. 88631

17 ((hospital$ or inpatient$ or in-patient$) adj2 (stay$ or admission$ or episode$)).ti,ab,ot,kw,kf. 316329

18 ((length$ or hospital$ or inpatient$ or in-patient$ or ICU or HDU or "intensive care" or "high dependancy") adj2 stay$).ti,ab,ot,kw,kf. 349093

19 ((outpatient$ or outpatient$ or inpatient$ or in-patient$ or ambulatory or emergenc$) adj2 care).ti,ab,ot,kw,kf. 105429

20 (cost$ adj2 (burden$ or estimat$ or variabl$ or hospital$ or control$)).ti,ab,ot,kw,kf. 83067

21 (district nurs$ or health visitor$ or health visiting or community nurs$ or community care or community visit$ or home visit$).ti,ab,ot,kf,kw. 30935

22 (in-patient stay$ or inpatient stay$).ti,ab,ot,hw. 7693

23 ((ambulatory or ambulance or hospital or A&E or emergency) adj2 (attention$ or trip or trips or visit$ or stay$ or admission$ or admitted or transport$)).ti,ab,ot,kw,kf. 359162

24 or/1-17 1324101

25 health-economics/ 35914

26 exp economic evaluation/ 356729

27 exp health care cost/ 342662

28 exp pharmacoeconomics/ 232733

29 (econom$ or cost or costs or costly or costing or price or prices or pricing or pharmacoeconomic$).ti,ab. 1399717

30 (expenditure$ not energy).ti,ab. 50693

31 (value adj2 money).ti,ab. 3022

32 budget$.ti,ab. 47473

33 or/25-32 1812784

34 ((metabolic adj cost) or (energy adj cost) or (energy adj expenditure)).ti,ab. 42464

35 33 not 34 1804669

36 exp quality of life/ 656367

37 (sf36 or sf 36 or sf-36 or short form 36 or shortform 36 or sf thirtysix or sf thirty six or shortform thirtysix or shortform thirty six or short form thirty six or short form thirtysix or short form thirty six).ti,ab,ot. 49973

38 (sf6 or sf 6 or sf-6 or short form 6 or shortform 6 or sf six or sfsix or shortform six or short form six).ti,ab,ot. 2974

39 (sf12 or sf 12 or sf-12 or short form 12 or shortform 12 or sf twelve or sftwelve or shortform twelve or short form twelve).ti,ab,ot. 12234

40 (sf6D or sf 6D or sf-6D or short form 6D or shortform 6D or sf six D or sfsixD or shortform six D or short form six D).ti,ab,ot. 1843

41 (sf20 or sf 20 or sf-20 or short form 20 or shortform 20 or sf twenty or sftwenty or shortform twenty or short form twenty).ti,ab,ot. 528

42 (sf8 or sf 8 or sf-8 or short form 8 or shortform 8 or sf eight or sfeight or shortform eight or short form eight).ti,ab,ot. 1201

43 "health related quality of life".ti,ab,ot. 84977

44 (Quality adjusted life or Quality-adjusted-life).ti,ab,ot. 26491

45 "assessment of quality of life".ti,ab,ot. 3570

46 (eq-5d or eq5d or eq-5 or eq5 or euro qual or euroqual or euro qual5d or euroqual5d or euro qol or euroqol or euro qol5d or euroqol5d or euro quol or euroquol or euro quol5d or euroquol5d or eur qol or eurqol or eur qol5d or eur qol5d or eur?qul or eur?qul5d or euro$ quality of life or european qol).ti,ab,kf. 31432

47 (euro$ adj3 (5 d or 5d or 5 dimension$ or 5dimension$ or 5 domain$ or 5domain$)).ti,ab,kf. 9074

48 (hql or hrql or hqol or h qol or hrqol or hr qol or hye or hyes).ti,ab,ot. 46092

49 ((cough or coughing) adj3 frequen$).ti,ab,ot,kf,kw. 2271

50 health$ year$ equivalent$.ti,ab,ot. 41

51 (hui or hui1 or hui2 or hui3 or hui4 or hui-4 or hui-1 or hui-2 or hui-3).ti,ab,ot. 3988

52 (quality time or qwb or quality of well being or "quality of wellbeing" or "index of wellbeing" or "index of well being").ti,ab,ot,hw. 1578

53 (Disability adjusted life or Disability-adjusted life or health adjusted life or health-adjusted life or "years of healthy life" or healthy years equivalent or "years of potential life lost" or "years of health life lost").ti,ab,ot. 7524

54 (QALY$ or DALY$ or HALY$ or YHL or HYES or YPLL or YHLL or qald$ or qale$ or qtime$ or AQoL$).ti,ab,ot. 34620

55 (qald$ or qale$ or qtime$).ti,ab,kf. 424

56 (timetradeoff or time tradeoff or time trade-off or time trade off or TTO or Standard gamble$ or "willingness to pay").ti,ab,ot. 16964

57 15d.ti,ab,ot. 2939

58 (HSUV$ or health state$ value$ or health state$ preference$ or HSPV$).ti,ab,ot. 777

59 (utilit$ adj3 ("quality of life" or valu$ or scor$ or measur$ or health or life or estimat$ or elicit$ or disease$ or cost$ or disease$ or mean or gain or gains or index$)).ti,ab,ot. 37116

60 (utilities or disutili$).ti,ab,ot. 15845

61 (illness state$1 or health state$1).ti,ab,kf. 14742

62 (multiattribute$ or multi attribute$).ti,ab,kf. 1577

63 Cost-Benefit Analysis/ and (cost-effectiveness ratio$ and (perspective$ or life expectanc$)).ti,ab,kf. 1235

64 ((quality of life or qol) adj (score$1 or measure$1)).ti,ab,kf. 36887

65 questionnaire/ or open ended questionnaire/ or structured questionnaire/ 931425

66 health status indicator/ or chronic disease indicator/ or disease activity score/ or "severity of illness index"/ 32542

67 health survey/ 231200

68 interview/ or semi structured interview/ or structured interview/ or exp telephone interview/ or unstructured interview/ 355210

69 (survey or surveys or questionnaire$ or interview or interviews or "focus group$").ti,ab,ot,kf,kw. 2158642

70 (PRO or PROs or patient reported outcome$).ti,ab,ot,kf,kw. 454044

71 (CRQ or PICQol or "CAP-sym" or "MRF26" or "MRF-26" or "AQ30" or "AQ-30" or "AQ20" or "AQ-20" or BPQ or CRQ or PFSDQ$ or SGRQ or LCQ or K-BILD or BILD).ti,ab,ot,kf,kw. 7721

72 or/36-71 3377857

73 or/24,35,72 5678125

74 animal/ or animal experiment/ 4669819

75 (rat or rats or mouse or mice or murine or rodent or rodents or hamster or hamsters or pig or pigs or porcine or rabbit or rabbits or animal or animals or dogs or dog or cats or cow or bovine or sheep or ovine or monkey or monkeys).ti,ab,ot,hw. 7652750

76 or/74-75 7652750

77 exp human/ or human experiment/ 25600557

78 76 not (76 and 77) 5740320

79 73 not 78 5411542

80 79 not (letter or editorial).pt. 5232869

81 coughing/ or barking cough/ or chronic cough/ or dry cough/ or experimental coughing/ or hacking cough/ or irritative coughing/ or paroxysmal cough/ or productive cough/ or psychogenic cough/ 157808

82 cough$.ti,ab,ot,kf,kw,hw. 189448

83 or/81-82 189448

84 exp interstitial lung disease/ 119373

85 ((Interstitial or diffuse parenchymal) adj3 lung adj2 (disorder$ or disease$)).ti,ab,ot,kf,kw. 31393

86 (ILD or ILDs or DPLD or IPD or IIP or IPF or DIP or AIP or NSIP or RBILD or RB-ILD or COP or BOOP or CWP).ti,ab,ot,kf,kw. 73753

87 pulmonary fibrosis.ti,ab,ot,kf,kw. 39501

88 ((Interstitial or diffuse parenchymal) adj3 pulmonary adj2 (disease$ or disorder$)).ti,ab,ot,kf,kw. 1730

89 (Interstitial adj3 (Pneumonia$ or Pneumonitides or Pneumonitis or pneumopath$ or pneumocystic)).ti,ab,ot,kf,kw. 19171

90 (chronic fibrous pneumonia$ or fibroid phthisis or phthisis fibroidea or pneumonia chronica fibrosa or pneumonia fibrosa chronica or pneumonia interstitialis or pneumonitis acuta).ti,ab,ot,kf,kw. 1

91 ((carrington$ or Loeffler$ or Loffler$ or caplan$ or Hamman-Rich$ or Antisynthetase or Anti-synthetase) adj2 (disease$ or disorder$ or syndrome$)).ti,ab,ot,kf,kw. 2160

92 (eosinophil$ adj3 (pleurisy or pleuritis or lung or lungs or loeffler$ or Loffler$ or pneumonia$ or infiltrate or pulmonary)).ti,ab,ot,kf,kw. 8947

93 ((wegener$ or polyangiitis or necrotising or respiratory or necrotizing or pneumogenic) adj2 granulomatosis).ti,ab,ot,kf,kw. 13543

94 (wegener$ adj2 (syndrome$ or disease$ or disorder$ or morbus or granuloma$ or churg or klinger)).ti,ab,ot,kf,kw. 8017

95 (lung coniosis or pneumoconiosis or pneumoconiotic or pneumokoniosis or pneumonoconiosis or pneumonokoniosis or silicoarthritis).ti,ab,ot,kf,kw. 4782

96 ((Cryptogenic organizing or Cryptogenic organising or bronchiolitis obliterans) adj3 pneumonia$).ti,ab,ot,kf,kw. 2391

97 ((thatched roof or maple bark or detergent worker$ or welder$) adj2 (disease$ or disorder$ or syndrome$)).ti,ab,ot,kf,kw. 61

98 ((coal miner$ or coalworker$ or worker$ or black or aluminium or welder$ or iron miner$ or iron-miner$ or labrador or bird fancier$ or indium or bird breeder$ or pigeon keeper$ or cheese washer$ or compost or farmer$ or hot tub or humidifier$ or Japanese summer house or maple bark or miller$ or peat moss$ or snuff or trombone Player$ or Brass Player$ or Wine-grower$ or woodworker$) adj2 lung$).ti,ab,ot,kf,kw. 2109

99 (Asbestosis or silicosis or anthracosis or Aluminosis or siderosis or Berylliosis or Byssinosis or Chalicosis or Silicosiderosis or Stannosis or Talcosis or Baritosis or bagassosis or Lycoperdonosis or Sequoiosis or Suberosis).ti,ab,ot,kf,kw. 11706

100 ((Bauxite or beryllium) adj2 (disease$ or disorder$ or poisoning or syndrome$ or fibrosis)).ti,ab,ot,kf,kw. 596

101 (pulmonary adj2 (haemosiderosis or haemo-siderosis)).ti,ab,ot,kf,kw. 168

102 ((Hypersensitivity or hyper-sensitivity) adj2 pneumonitis).ti,ab,ot,kf,kw. 5007

103 extrinsic allergic alveolitis.ti,ab,ot,kf,kw. 1066

104 ((Cephalosporium or Isocyanate or Trimellitic anhydride or TMA or Familial or Domestic or Japanese summer or Metalwork$ or Metal-work$ or Mollusc or shell or Sauna$ or worker$ or Streptomyces or Tap water) adj2 HP).ti,ab,ot,kf,kw. 66

105 Fibrosing alveolitis.ti,ab,ot,kf,kw. 1082

106 ((alveolar or lung$ or interstitial or pulmonary) adj3 fibrosis).ti,ab,ot,kf,kw. 71334

107 or/84-106 233009

108 80 and 83 and 107 2463

**109 limit 108 to yr="2010 -Current" 2088**

Humanistic burden terms based on:

Clayton S, Bambra C, Gosling R, Povall S, Misso K, Whitehead M. Assembling the evidence jigsaw: insights from a systematic review of UK studies of individual-focused return to work initiatives for disabled and long-term ill people [Internet]. BMC Public Health 2011 [cited 23.3.11];11:170.

Cost-effectiveness filter based on:

Centre for Reviews and Dissemination. Search strategies: NHS EED EMBASE using OvidSP (economics filter) [Internet]. York: Centre for Reviews and Dissemination; 2014 [accessed 2.6.14]. Available from:

http://www.crd.york.ac.uk/crdweb/searchstrategies.asp#nhseedembase

HRQoL free-text terms based on:

Figure 4: Common free-text terms for electronic database searching for HSUVs in Papaioannou D, Brazier JE, Paisley S. NICE DSU Technical Support Document 9: the identification, review and synthesis of health state utility values from the literature (Internet), 2011 (accessed: 18.8.11) Available from: http://www.nicedsu.org.uk

Arber M, Garcia S, Veale T, Edwards M, Shaw A, Glanville JM. Performance of Ovid Medline search filters to identify health state utility studies. Int J Technol Assess Health Care 2017;33(4):472-80.

**Medline ALL (Ovid): 1946-2023/10/02**

**Searched 3.10.23**

1 Efficiency/ 15647

2 Absenteeism/ 9788

3 Caregiver Burden/ 639

4 Caregivers/ or Stress, Psychological/ or Financial Stress/ or exp Occupational Stress/ 197542

5 ((human$ or social$ or societ$ or work$ or employe$ or business$ or communit$ or famil$ or carer$ or caregiver$) adj3 (burden$ or consequenc$ or impact$ or problem$ or productivity or sickness or impairment$)).ti,ab,ot,hw. 168622

6 ((long standing or longstanding or long term or longterm or permanent or employee$) adj2 (absence$ or absent$ or ill$ or sick$ or disab$)).ti,ab,ot,hw. 13421

7 (llsi or ((emergenc$ or domestic$ or famil$ or carer$ or caregiver$) adj3 leave$)).ti,ab,ot. 1180

8 ((cost or costs or burden) adj2 (illness$ or disease$ or sickness$)).ti,ab,ot,kw,kf. 46041

9 ((allowance or status or long-term or pension$ or benefit$) adj2 (disab$ or incapacit$)).ti,ab,ot,hw. 16863

10 ((unable or inability or incapacit$ or incapab$) adj3 work).ti,ab,ot,hw. 2218

11 ((resource$ or fund$) adj2 (use$ or utili?ation)).ti,ab,ot,hw. 43418

12 ((health or healthcare) adj2 (resource$ or fund$)).ti,ab,ot,hw. 53641

13 ("length of stay" or "duration of stay" or "extended stay" or "prolonged stay" or "stay days").ti,ab,ot,kw,kf. 84447

14 ((ambulatory or ambulance or hospital or A&E or emergency) adj2 (attention$ or trip or trips or visit$ or stay$ or admission$ or admitted or transport$)).ti,ab,ot,hw. 218023

15 ((GP or general practitioner$ or doctor$ or inpatient$ or in-patient$ or clinician$ or specialist$ or physician$ or clinic or clinics or outpatient$ or out-patient$ or surger$) adj2 (appointment$ or attention$ or trip or trips or visit$)).ti,ab,ot,kw,kf. 49116

16 ((hospital$ or inpatient$ or in-patient$) adj2 (stay$ or admission$ or episode$)).ti,ab,ot,kw,kf. 188874

17 ((length$ or hospital$ or inpatient$ or in-patient$ or ICU or HDU or "intensive care" or "high dependancy") adj2 stay$).ti,ab,ot,kw,kf. 200047

18 ((outpatient$ or outpatient$ or inpatient$ or in-patient$ or ambulatory or emergenc$) adj2 care).ti,ab,ot,kw,kf. 72027

19 (cost$ adj2 (burden$ or estimat$ or variabl$ or hospital$ or control$)).ti,ab,ot,kw,kf. 52791

20 (district nurs$ or health visitor$ or health visiting or community nurs$ or community care or community visit$ or home visit$).ti,ab,ot,kf,kw. 25828

21 (in-patient stay$ or inpatient stay$).ti,ab,ot,hw. 3792

22 ((ambulatory or ambulance or hospital or A&E or emergency) adj2 (attention$ or trip or trips or visit$ or stay$ or admission$ or admitted or transport$)).ti,ab,ot,kw,kf. 218829

23 or/1-22 960169

24 economics/ or economics, dental/ or exp "economics, hospital"/ or economics, medical/ or economics, nursing/ or socioeconomics/ or exp fee/ or cost/ 139102

25 "Costs and Cost Analysis"/ 51559

26 Economics, Pharmaceutical/ 3110

27 (econom$ or cost or costs or costly or costing or price or prices or pricing or pharmacoeconomic$).ti,ab. 1097271

28 (expenditure$ not energy).ti,ab. 37445

29 (value adj2 money).ti,ab. 2205

30 budget$.ti,ab. 36033

31 or/24-30 1207612

32 ((metabolic adj cost) or (energy adj cost) or (energy adj expenditure)).ti,ab. 34144

33 31 not 32 1200131

34 "Quality of Life"/ 273468

35 (sf36 or sf 36 or sf-36 or short form 36 or shortform 36 or sf thirtysix or sf thirty six or shortform thirtysix or shortform thirty six or short form thirty six or short form thirtysix or short form thirty six).ti,ab,ot. 30763

36 (sf6 or sf 6 or sf-6 or short form 6 or shortform 6 or sf six or sfsix or shortform six or short form six).ti,ab,ot. 2665

37 (sf12 or sf 12 or sf-12 or short form 12 or shortform 12 or sf twelve or sftwelve or shortform twelve or short form twelve).ti,ab,ot. 7688

38 (sf6D or sf 6D or sf-6D or short form 6D or shortform 6D or sf six D or sfsixD or shortform six D or short form six D).ti,ab,ot. 1009

39 (sf20 or sf 20 or sf-20 or short form 20 or shortform 20 or sf twenty or sftwenty or shortform twenty or short form twenty).ti,ab,ot. 462

40 (sf8 or sf 8 or sf-8 or short form 8 or shortform 8 or sf eight or sfeight or shortform eight or short form eight).ti,ab,ot. 757

41 "health related quality of life".ti,ab,ot. 58736

42 (Quality adjusted life or Quality-adjusted-life).ti,ab,ot. 17479

43 (eq-5d or eq5d or eq-5 or eq5 or euro qual or euroqual or euro qual5d or euroqual5d or euro qol or euroqol or euro qol5d or euroqol5d or euro quol or euroquol or euro quol5d or euroquol5d or eur qol or eurqol or eur qol5d or eur qol5d or eur?qul or eur?qul5d or euro$ quality of life or european qol).ti,ab,kf. 17707

44 (euro$ adj3 (5 d or 5d or 5 dimension$ or 5dimension$ or 5 domain$ or 5domain$)).ti,ab,kf. 6093

45 (hql or hrql or hqol or h qol or hrqol or hr qol or hye or hyes).ti,ab,ot. 28272

46 ((cough or coughing) adj3 frequen$).ti,ab,ot,kf,kw. 1385

47 health$ year$ equivalent$.ti,ab,ot. 40

48 (hui or hui1 or hui2 or hui3 or hui4 or hui-4 or hui-1 or hui-2 or hui-3).ti,ab,ot. 1988

49 (quality time or qwb or quality of well being or "quality of wellbeing" or "index of wellbeing" or "index of well being").ti,ab,ot,hw. 1188

50 (Disability adjusted life or Disability-adjusted life or health adjusted life or health-adjusted life or "years of healthy life" or healthy years equivalent or "years of potential life lost" or "years of health life lost").ti,ab,ot. 6320

51 (qald$ or qale$ or qtime$).ti,ab,kf. 235

52 (timetradeoff or time tradeoff or time trade-off or time trade off or TTO or Standard gamble$ or "willingness to pay").ti,ab,ot. 11376

53 15d.ti,ab,ot. 1972

54 (HSUV$ or health state$ value$ or health state$ preference$ or HSPV$).ti,ab,ot. 508

55 (utilit$ adj3 ("quality of life" or valu$ or scor$ or measur$ or health or life or estimat$ or elicit$ or disease$ or cost$ or disease$ or mean or gain or gains or index$)).ti,ab,ot. 23373

56 (utilities or disutili$).ti,ab,ot. 9882

57 (illness state$1 or health state$1).ti,ab,kf. 8499

58 (multiattribute$ or multi attribute$).ti,ab,kf. 1346

59 Cost-Benefit Analysis/ and (cost-effectiveness ratio$ and (perspective$ or life expectanc$)).ti,ab,kf. 5293

60 ((quality of life or qol) adj (score$1 or measure$1)).ti,ab,kf. 22075

61 "surveys and questionnaires"/ or exp health care surveys/ or exp health surveys/ or patient health questionnaire/ 1164552

62 health status indicators/ or chronic disease indicators/ or sickness impact profile/ 31107

63 "Severity of Illness Index"/ 271096

64 health surveys/ or exp health status indicators/ 404930

65 interview/ 30778

66 Interviews as Topic/ 66836

67 (survey or surveys or questionnaire$ or interview or interviews or "focus group$").ti,ab,ot,kf,kw. 1634610

68 (PRO or PROs or patient reported outcome$).ti,ab,ot,kf,kw. 304337

69 (CRQ or PICQol or "CAP-sym" or "MRF26" or "MRF-26" or "AQ30" or "AQ-30" or "AQ20" or "AQ-20" or BPQ or CRQ or PFSDQ$ or SGRQ or LCQ or K-BILD or BILD).ti,ab,ot,kf,kw. 5135

70 or/34-69 2764494

71 or/23,33,70 4296552

72 exp Animals/ not (exp Animals/ and Humans/) 5159763

73 71 not 72 4078556

74 73 not (comment or editorial or letter).pt. 3964910

75 Cough/ 18739

76 cough$.ti,ab,ot,kf,kw,hw. 76490

77 or/75-76 76490

78 exp Lung Diseases, Interstitial/ or exp Agricultural Workers' Diseases/ or Asthma, Occupational/ or Bird Fancier's Lung/ or exp Pneumoconiosis/ 91618

79 ((Interstitial or diffuse parenchymal) adj3 lung adj2 (disorder$ or disease$)).ti,ab,ot,kf,kw. 17068

80 (ILD or ILDs or DPLD or IPD or IIP or IPF or DIP or AIP or NSIP or RBILD or RB-ILD or COP or BOOP or CWP).ti,ab,ot,kf,kw. 47895

81 pulmonary fibrosis.ti,ab,ot,kf,kw. 25380

82 ((Interstitial or diffuse parenchymal) adj3 pulmonary adj2 (disease$ or disorder$)).ti,ab,ot,kf,kw. 1042

83 (Interstitial adj3 (Pneumonia$ or Pneumonitides or Pneumonitis or pneumopath$ or pneumocystic)).ti,ab,ot,kf,kw. 12926

84 (chronic fibrous pneumonia$ or fibroid phthisis or phthisis fibroidea or pneumonia chronica fibrosa or pneumonia fibrosa chronica or pneumonia interstitialis or pneumonitis acuta).ti,ab,ot,kf,kw. 2

85 ((carrington$ or Loeffler$ or Loffler$ or caplan$ or Hamman-Rich$ or Antisynthetase or Anti-synthetase) adj2 (disease$ or disorder$ or syndrome$)).ti,ab,ot,kf,kw. 1813

86 (eosinophil$ adj3 (pleurisy or pleuritis or lung or lungs or loeffler$ or Loffler$ or pneumonia$ or infiltrate or pulmonary)).ti,ab,ot,kf,kw. 6176

87 ((wegener$ or polyangiitis or necrotising or respiratory or necrotizing or pneumogenic) adj2 granulomatosis).ti,ab,ot,kf,kw. 9426

88 (wegener$ adj2 (syndrome$ or disease$ or disorder$ or morbus or granuloma$ or churg or klinger)).ti,ab,ot,kf,kw. 6386

89 (lung coniosis or pneumoconiosis or pneumoconiotic or pneumokoniosis or pneumonoconiosis or pneumonokoniosis or silicoarthritis).ti,ab,ot,kf,kw. 5654

90 ((Cryptogenic organizing or Cryptogenic organising or bronchiolitis obliterans) adj3 pneumonia$).ti,ab,ot,kf,kw. 1441

91 ((thatched roof or maple bark or detergent worker$ or welder$) adj2 (disease$ or disorder$ or syndrome$)).ti,ab,ot,kf,kw. 52

92 ((coal miner$ or coalworker$ or worker$ or black or aluminium or welder$ or iron miner$ or iron-miner$ or labrador or bird fancier$ or indium or bird breeder$ or pigeon keeper$ or cheese washer$ or compost or farmer$ or hot tub or humidifier$ or Japanese summer house or maple bark or miller$ or peat moss$ or snuff or trombone Player$ or Brass Player$ or Wine-grower$ or woodworker$) adj2 lung$).ti,ab,ot,kf,kw. 1741

93 (Asbestosis or silicosis or anthracosis or Aluminosis or siderosis or Berylliosis or Byssinosis or Chalicosis or Silicosiderosis or Stannosis or Talcosis or Baritosis or bagassosis or Lycoperdonosis or Sequoiosis or Suberosis).ti,ab,ot,kf,kw. 12615

94 ((Bauxite or beryllium) adj2 (disease$ or disorder$ or poisoning or syndrome$ or fibrosis)).ti,ab,ot,kf,kw. 635

95 (pulmonary adj2 (haemosiderosis or haemo-siderosis)).ti,ab,ot,kf,kw. 194

96 ((Hypersensitivity or hyper-sensitivity) adj2 pneumonitis).ti,ab,ot,kf,kw. 2991

97 extrinsic allergic alveolitis.ti,ab,ot,kf,kw. 621

98 ((Cephalosporium or Isocyanate or Trimellitic anhydride or TMA or Familial or Domestic or Japanese summer or Metalwork$ or Metal-work$ or Mollusc or shell or Sauna$ or worker$ or Streptomyces or Tap water) adj2 HP).ti,ab,ot,kf,kw. 49

99 Fibrosing alveolitis.ti,ab,ot,kf,kw. 887

100 ((alveolar or lung$ or interstitial or pulmonary) adj3 fibrosis).ti,ab,ot,kf,kw. 44948

101 or/78-100 173078

102 74 and 77 and 101 640

**103 limit 102 to yr="2010 -Current" 395**

Humanistic burden terms based on:

Clayton S, Bambra C, Gosling R, Povall S, Misso K, Whitehead M. Assembling the evidence jigsaw: insights from a systematic review of UK studies of individual-focused return to work initiatives for disabled and long-term ill people [Internet]. BMC Public Health 2011 [cited 23.3.11];11:170.

Cost-effectiveness filter based on:

Centre for Reviews and Dissemination. Search strategies: NHS EED EMBASE using OvidSP (economics filter) [Internet]. York: Centre for Reviews and Dissemination; 2014 [accessed 2.6.14]. Available from:

http://www.crd.york.ac.uk/crdweb/searchstrategies.asp#nhseedembase

HRQoL free-text terms based on:

Figure 4: Common free-text terms for electronic database searching for HSUVs in Papaioannou D, Brazier JE, Paisley S. NICE DSU Technical Support Document 9: the identification, review and synthesis of health state utility values from the literature (Internet), 2011 (accessed: 18.8.11) Available from: http://www.nicedsu.org.uk

Arber M, Garcia S, Veale T, Edwards M, Shaw A, Glanville JM. Performance of Ovid Medline search filters to identify health state utility studies. Int J Technol Assess Health Care 2017;33(4):472-80.

**Cochrane Database of Systematic Reviews (CDSR) (Wiley): Issue 10/12 October 2023**

**Searched 3.10.23**

**Limited 2010-2023/10/03**

#1 MeSH descriptor: [Lung Diseases, Interstitial] explode all trees 1583

#2 MeSH descriptor: [Agricultural Workers' Diseases] explode all trees 52

#3 MeSH descriptor: [Asthma, Occupational] this term only 3

#4 MeSH descriptor: [Bird Fancier's Lung] this term only 3

#5 MeSH descriptor: [Pneumoconiosis] explode all trees 130

#6 ((Interstitial OR "diffuse parenchymal") NEAR/3 lung NEAR/2 (disorder* OR disease*)):ti,ab 1409

#7 (ILD OR ILDs OR DPLD OR IPD OR IIP OR IPF OR DIP OR AIP OR NSIP OR RBILD OR RB-ILD OR COP OR BOOP OR CWP):ti,ab 4602

#8 "pulmonary fibrosis":ti,ab 1801

#9 ((Interstitial OR "diffuse parenchymal") NEAR/3 pulmonary NEAR/2 (disease* OR disorder*)):ti,ab 142

#10 (Interstitial NEAR/3 (Pneumonia* OR Pneumonitides OR Pneumonitis OR pneumopath* OR pneumocystic)):ti,ab 499

#11 ("chronic fibrous pneumonia" OR "fibroid phthisis" OR "phthisis fibroidea" OR "pneumonia chronica fibrosa" OR "pneumonia fibrosa chronica" OR "pneumonia interstitialis" OR "pneumonitis acuta"):ti,ab 0

#12 ((carrington* OR Loeffler* OR Loffler* OR caplan* OR Hamman-Rich* OR Antisynthetase OR "Anti-synthetase") NEAR/2 (disease* OR disorder* OR syndrome*)):ti,ab 22

#13 (eosinophil* NEAR/3 (pleurisy OR pleuritis OR lung OR lungs OR loeffler* OR Loffler* OR pneumonia* OR infiltrate OR pulmonary)):ti,ab 134

#14 ((wegener* OR polyangiitis OR necrotising OR respiratory OR necrotizing OR pneumogenic) NEAR/2 granulomatosis):ti,ab 340

#15 (wegener* NEAR/2 (syndrome* OR disease* OR disorder* OR morbus OR granuloma* OR churg OR klinger)):ti,ab 135

#16 ("lung coniosis" OR pneumoconiosis OR pneumoconiotic OR pneumokoniosis OR pneumonoconiosis OR pneumonokoniosis OR silicoarthritis):ti,ab 86

#17 (("Cryptogenic organizing" OR "Cryptogenic organizing" OR "bronchiolitis obliterans") NEAR/3 pneumonia*):ti,ab 16

#18 (("thatched roof" OR "maple bark" OR "detergent worker" OR "detergent workers" OR welder*) NEAR/2 (disease* OR disorder* OR syndrome*)):ti,ab 0

#19 ((compost OR farmer* OR "hot tub" OR humidifier* OR "Japanese summer house" OR "maple bark" OR miller* OR "peat moss" OR snuff OR "trombone Player" OR "Brass Player" OR "Wine-grower") NEAR/2 lung*):ti,ab 2290

#20 (("coal miner" OR coalworker* OR worker* OR black OR aluminium OR welder* OR "iron miner" OR "iron-miner" OR labrador OR "bird fancer" OR indium OR "bird breeder" OR "pigeon keeper" OR "cheese washer") NEAR/2 lung*):ti,ab 7

#21 ((woodworker* OR "coal miners" OR "iron miners" OR "iron-miners" OR "bird fancers" OR "bird breeders" OR "pigeon keepers" OR "cheese washers" OR "peat mosses" OR "trombone Players" OR "Brass Players" OR "Wine-growers") NEAR/2 lung*):ti,ab 21

#22 (Asbestosis OR silicosis OR anthracosis OR Aluminosis OR siderosis OR Berylliosis OR Byssinosis OR Chalicosis OR Silicosiderosis OR Stannosis OR Talcosis OR Baritosis OR bagassosis OR Lycoperdonosis OR Sequoiosis OR Suberosis):ti,ab 153

#23 ((Bauxite OR beryllium) NEAR/2 (disease* OR disorder* OR poisoning OR syndrome* OR fibrosis)):ti,ab 12

#24 (pulmonary NEAR/2 (haemosiderosis OR haemo-siderosis)):ti,ab 0

#25 ((Hypersensitivity OR "hyper-sensitivity") NEAR/2 pneumonitis):ti,ab 5667

#26 "extrinsic allergic alveolitis":ti,ab 1

#27 ((Cephalosporium OR Isocyanate OR "Trimellitic anhydride" OR TMA OR Familial OR Domestic OR "Japanese summer" OR Metalwork* OR "Metal-work" OR "Metal-worker" OR "Metal-workers" OR Mollusc OR shell OR Sauna* OR worker* OR Streptomyces OR "Tap water") NEAR/2 HP):ti,ab 0

#28 "Fibrosing alveolitis":ti,ab 19

#29 ((alveolar OR lung* OR interstitial OR pulmonary) NEAR/3 fibrosis):ti,ab 2659

#30 #1 OR #2 OR #3 OR #4 OR #5 OR #6 OR #7 OR #8 OR #9 OR #10 OR #11 OR #12 OR #13 OR #14 OR #15 OR #16 OR #17 OR #18 OR #19 OR #22 OR #23 OR #24 OR #25 OR #26 OR #27 OR #28 OR #29 15719

#31 MeSH descriptor: [Cough] this term only 1653

#32 cough*:ti,ab,kw 18311

#33 #31 OR #32 18311

**#34 #30 AND #33 with Cochrane Library publication date Between Jan 2010 and Oct 2023, in Cochrane Reviews, Cochrane Protocols 8**

**Protocols = 0**

**Reviews = 8**

**Cochrane Central Register of Controlled Trials (CENTRAL) (Wiley): Issue 10, October 2023**

**Searched 3.10.23**

**Limited 2010-2023/10/03**

#1 MeSH descriptor: [Absenteeism] this term only 672

#2 MeSH descriptor: [Efficiency] this term only 407

#3 MeSH descriptor: [Caregiver Burden] this term only 174

#4 MeSH descriptor: [Caregivers] this term only 3253

#5 MeSH descriptor: [Stress, Psychological] this term only 7139

#6 MeSH descriptor: [Financial Stress] this term only 21

#7 MeSH descriptor: [Occupational Stress] explode all trees 682

#8 ((human* OR social* OR societ* OR work* OR employe* OR business* OR communit* OR famil* OR carer* OR caregiver*) NEAR/3 (burden* OR consequenc* OR impact* OR problem* OR productivity OR sickness OR impairment*)):ti,ab,kw 17574

#9 ((long standing OR longstanding OR long term OR longterm OR permanent OR employee*) NEAR/2 (absence* OR absent* OR ill* OR sick* OR disab*)):ti,ab,kw 1805

#10 (llsi OR ((emergenc* OR domestic* OR famil* OR carer* OR caregiver*) NEAR/3 leave*)):ti,ab 47

#11 ((cost OR costs OR burden) NEAR/2 (illness* OR disease* OR sickness*)):ti,ab 3581

#12 ((allowance OR status OR long-term OR pension* OR benefit*) NEAR/2 (disab* OR incapacit*)):ti,ab,kw 4042

#13 ((unable OR inability OR incapacit* OR incapab*) NEAR/3 work):ti,ab,kw 294

#14 ((resource* OR fund*) NEAR/2 (use* OR utili?ation)):ti,ab,kw 6605

#15 ((health OR healthcare) NEAR/2 (resource* OR fund*)):ti,ab,kw 6290

#16 ("length of stay" OR "duration of stay" OR "extended stay" OR "prolonged stay" OR "stay days"):ti,ab 14265

#17 ((ambulatory OR ambulance OR hospital OR A&E OR emergency) NEAR/2 (attention* OR trip OR trips OR visit* OR stay* OR admission* OR admitted OR transport*)):ti,ab,kw 46354

#18 ((GP OR general practitioner* OR doctor* OR inpatient* OR in-patient* OR clinician* OR specialist* OR physician* OR clinic OR clinics OR outpatient* OR out-patient* OR surger*) NEAR/2 (appointment* OR attention* OR trip OR trips OR visit*)):ti,ab 13144

#19 ((hospital* OR inpatient* OR in-patient*) NEAR/2 (stay* OR admission* OR episode*)):ti,ab 38836

#20 ((length* OR hospital* OR inpatient* OR in-patient* OR ICU OR HDU OR "intensive care" OR "high dependancy") NEAR/2 stay*):ti,ab 42110

#21 ((outpatient* OR outpatient* OR inpatient* OR in-patient* OR ambulatory OR emergenc*) NEAR/2 care):ti,ab 8527

#22 (cost* NEAR/2 (burden* OR estimat* OR variabl* OR hospital* OR control*)):ti,ab 8068

#23 (district nurs* OR health visitor* OR health visiting OR community nurs* OR community care OR community visit* OR home visit*):ti,ab 34156

#24 (in-patient stay* OR inpatient stay*):ti,ab,kw 4049

#25 ((ambulatory OR ambulance OR hospital OR A&E OR emergency) NEAR/2 (attention* OR trip OR trips OR visit* OR stay* OR admission* OR admitted OR transport*)):ti,ab 42349

#26 #1 OR #2 OR #3 OR #4 OR #5 OR #6 OR #7 OR #8 OR #9 OR #10 OR #11 OR #12 OR #13 OR #14 OR #15 OR #16 OR #17 OR #18 OR #19 OR #20 OR #21 OR #22 OR #23 OR #24 OR #25 149463

#27 MeSH descriptor: [Economics] this term only 84

#28 MeSH descriptor: [Economics, Dental] this term only 2

#29 MeSH descriptor: [Economics, Hospital] explode all trees 823

#30 MeSH descriptor: [Economics, Medical] this term only 32

#31 MeSH descriptor: [Economics, Nursing] this term only 13

#32 MeSH descriptor: [Socioeconomic Factors] explode all trees 13872

#33 MeSH descriptor: [Fees and Charges] explode all trees 321

#34 MeSH descriptor: [Costs and Cost Analysis] explode all trees 14632

#35 MeSH descriptor: [Economics, Pharmaceutical] this term only 121

#36 (econom* OR cost OR costs OR costly OR costing OR price OR prices OR pricing OR pharmacoeconomic*):ti,ab 94719

#37 (expenditure* not energy):ti,ab 2278

#38 (value NEAR/2 money):ti,ab 299

#39 budget*:ti,ab 1458

#40 #27 or #28 or #29 or #30 or #31 or #32 or #33 or #34 or #35 or #36 or #37 or #38 or #39 109541

#41 ((metabolic NEAR cost) OR (energy NEAR cost) OR (energy NEAR expenditure)):ti,ab 5433

#42 #40 not #41 108728

#43 MeSH descriptor: [Quality of Life] this term only 44040

#44 ("sf36" OR "sf 36" OR "sf-36" OR "short form 36" OR "shortform 36" OR "sf thirtysix" OR "sf thirty six" OR "shortform thirtysix" OR "shortform thirty six" OR "short form thirty six" OR "short form thirtysix" OR "short form thirty six"):ti,ab 15916

#45 ("sf6" OR "sf 6" OR "sf-6" OR "short form 6" OR "shortform 6" OR "sf six" OR sfsix OR "shortform six" OR "short form six"):ti,ab 283

#46 ("sf12" OR "sf 12" OR "sf-12" OR "short form 12" OR "shortform 12" OR "sf twelve" OR sftwelve OR "shortform twelve" OR "short form twelve"):ti,ab 3501

#47 ("sf6D" OR "sf 6D" OR "sf-6D" OR "short form 6D" OR "shortform 6D" OR "sf six D" OR "sfsixD" OR "shortform six D" OR "short form six D"):ti,ab 362

#48 ("sf20" OR "sf 20" OR "sf-20" OR "short form 20" OR "shortform 20" OR "sf twenty" OR "sftwenty" OR "shortform twenty" OR "short form twenty"):ti,ab 107

#49 ("sf8" OR "sf 8" OR "sf-8" OR "short form 8" OR "shortform 8" OR "sf eight" OR "sfeight" OR "shortform eight" OR "short form eight"):ti,ab 330

#50 "health related quality of life":ti,ab 22320

#51 (Quality adjusted life OR Quality-adjusted-life):ti,ab 10092

#52 "assessment of quality of life":ti,ab 864

#53 ("eq-5d" OR "eq5d" OR "eq-5" OR "eq5" OR "euro qual" OR "euroqual" OR "euro qual5d" OR "euroqual5d" OR "euro qol" OR "euroqol" OR "euro qol5d" OR "euroqol5d" OR "euro quol" OR "euroquol" OR "euro quol5d" OR "euroquol5d" OR "eur qol" OR "eurqol" OR "eur qol5d" OR "eur qol5d" OR "euroqul" OR "euroqul5d" OR "euro quality of life" OR "european quality of life" OR "european qol"):ti,ab 13392

#54 (euro* NEAR/3 ("5 d" OR "5d" OR "5 dimension" OR "5dimension" OR "5 domain" OR "5domain" OR "5 dimensions" OR "5dimensions" OR "5 domains" OR "5domains")):ti,ab 3830

#55 (hql OR hrql OR hqol OR h qol OR hrqol OR "hr qol" OR hye OR hyes):ti,ab 9913

#56 ((cough OR coughing) NEAR/3 frequen*):ti,ab 661

#57 health* year* equivalent*:ti,ab 10612

#58 ("hui" OR "hui1" OR "hui2" OR "hui3" OR "hui4" OR "hui-4" OR "hui-1" OR "hui-2" OR "hui-3"):ti,ab 332

#59 ("quality time" OR qwb OR "quality of well being" OR "quality of wellbeing" OR "index of wellbeing" OR "index of well being"):ti,ab,kw 228

#60 ("Disability adjusted life" OR "Disability-adjusted life" OR "health adjusted life" OR "health-adjusted life" OR "years of healthy life" OR "healthy years equivalent" OR "years of potential life lost" OR "years of health life lost"):ti,ab 322

#61 (QALY* OR DALY* OR HALY* OR YHL OR HYES OR YPLL OR YHLL OR qald* OR qale* OR qtime* OR AQoL*):ti,ab 5589

#62 (qald* OR qale* OR qtime*):ti,ab 43

#63 (timetradeoff OR "time tradeoff" OR "time trade-off" OR "time trade off" OR TTO OR "Standard gamble" OR "Standard gambles" OR "willingness to pay"):ti,ab 2292

#64 "15d":ti,ab 310

#65 (HSUV* OR "health state value" OR "health state values" OR "health states value" OR "health states values" OR preference* OR HSPV*):ti,ab 18542

#66 (utilit* NEAR/3 ("quality of life" OR valu* OR scor* OR measur* OR health OR life OR estimat* OR elicit* OR disease* OR cost* OR disease* OR mean OR gain OR gains OR index*)):ti,ab 5498

#67 (utilities OR disutili*):ti,ab 1480

#68 ("illness state" OR "health state" OR "illness states" OR "health states"):ti,ab 1553

#69 (multiattribute* OR "multi attribute" OR "multi attributes"):ti,ab 97

#70 MeSH descriptor: [Cost-Benefit Analysis] this term only 9866

#71 (("cost-effectiveness ratio" OR "cost-effectiveness ratios") and (perspective* OR life expectanc*)):ti,ab 2080

#72 #70 AND #71 1009

#73 (("quality of life" OR qol) NEAR/3 (score* OR measure*)):ti,ab 24774

#74 #43 OR #44 OR #45 OR #46 OR #47 OR #48 OR #49 OR #50 OR #51 OR #52 OR #53 OR #54 OR #55 OR #57 OR #58 OR #59 OR #60 OR #61 OR #62 OR #63 OR #64 OR #65 OR #66 OR #67 OR #68 OR #69 OR #72 OR #73 125628

#75 MeSH descriptor: [Surveys and Questionnaires] this term only 32217

#76 MeSH descriptor: [Health Care Surveys] explode all trees 1912

#77 MeSH descriptor: [undefined] explode all trees 0

#78 MeSH descriptor: [Patient Health Questionnaire] this term only 139

#79 MeSH descriptor: [Health Status Indicators] this term only 1133

#80 MeSH descriptor: [Chronic Disease Indicators] this term only 0

#81 MeSH descriptor: [Sickness Impact Profile] this term only 608

#82 MeSH descriptor: [Severity of Illness Index] this term only 22754

#83 MeSH descriptor: [Health Surveys] this term only 1115

#84 MeSH descriptor: [Interview] this term only 822

#85 MeSH descriptor: [Interviews as Topic] this term only 2263

#86 (survey OR surveys OR questionnaire* OR interview OR interviews OR "focus group" OR "focus groups"):ti,ab 201593

#87 (PRO OR PROs OR "patient reported outcome" OR "patient reported outcomes"):ti,ab 32036

#88 (CRQ OR PICQol OR "CAP-sym" OR "MRF26" OR "MRF-26" OR "AQ30" OR "AQ-30" OR "AQ20" OR "AQ-20" OR BPQ OR CRQ OR PFSDQ* OR SGRQ OR LCQ OR "K-BILD" OR BILD):ti,ab 2532

#89 #75 OR #76 OR #77 OR #78 OR #79 OR #80 OR #81 OR #82 OR #83 OR #84 OR #85 OR #86 OR #87 OR #88 257938

#90 #26 OR #42 OR #74 OR #89 472732

#91 MeSH descriptor: [Cough] explode all trees 1653

#92 cough*:ti,ab,kw 18311

#93 #91 OR #92 18311

#94 MeSH descriptor: [Lung Diseases, Interstitial] explode all trees 1583

#95 MeSH descriptor: [Agricultural Workers' Diseases] explode all trees 52

#96 MeSH descriptor: [Asthma, Occupational] this term only 3

#97 MeSH descriptor: [Bird Fancier's Lung] this term only 3

#98 MeSH descriptor: [Pneumoconiosis] explode all trees 130

#99 ((Interstitial OR "diffuse parenchymal") NEAR/3 lung NEAR/2 (disorder* OR disease*)):ti,ab 1409

#100 (ILD OR ILDs OR DPLD OR IPD OR IIP OR IPF OR DIP OR AIP OR NSIP OR RBILD OR RB-ILD OR COP OR BOOP OR CWP):ti,ab 4602

#101 "pulmonary fibrosis":ti,ab 1801

#102 ((Interstitial OR "diffuse parenchymal") NEAR/3 pulmonary NEAR/2 (disease* OR disorder*)):ti,ab 142

#103 (Interstitial NEAR/3 (Pneumonia* OR Pneumonitides OR Pneumonitis OR pneumopath* OR pneumocystic)):ti,ab 499

#104 ("chronic fibrous pneumonia" OR "fibroid phthisis" OR "phthisis fibroidea" OR "pneumonia chronica fibrosa" OR "pneumonia fibrosa chronica" OR "pneumonia interstitialis" OR "pneumonitis acuta"):ti,ab 0

#105 ((carrington* OR Loeffler* OR Loffler* OR caplan* OR Hamman-Rich* OR Antisynthetase OR "Anti-synthetase") NEAR/2 (disease* OR disorder* OR syndrome*)):ti,ab 22

#106 (eosinophil* NEAR/3 (pleurisy OR pleuritis OR lung OR lungs OR loeffler* OR Loffler* OR pneumonia* OR infiltrate OR pulmonary)):ti,ab 134

#107 ((wegener* OR polyangiitis OR necrotising OR respiratory OR necrotizing OR pneumogenic) NEAR/2 granulomatosis):ti,ab 340

#108 (wegener* NEAR/2 (syndrome* OR disease* OR disorder* OR morbus OR granuloma* OR churg OR klinger)):ti,ab 135

#109 ("lung coniosis" OR pneumoconiosis OR pneumoconiotic OR pneumokoniosis OR pneumonoconiosis OR pneumonokoniosis OR silicoarthritis):ti,ab 86

#110 (("Cryptogenic organizing" OR "Cryptogenic organizing" OR "bronchiolitis obliterans") NEAR/3 pneumonia*):ti,ab 16

#111 (("thatched roof" OR "maple bark" OR "detergent worker" OR "detergent workers" OR welder*) NEAR/2 (disease* OR disorder* OR syndrome*)):ti,ab 0

#112 ((compost OR farmer* OR "hot tub" OR humidifier* OR "Japanese summer house" OR "maple bark" OR miller* OR "peat moss" OR snuff OR "trombone Player" OR "Brass Player" OR "Wine-grower") NEAR/2 lung*):ti,ab 2290

#113 (("coal miner" OR coalworker* OR worker* OR black OR aluminium OR welder* OR "iron miner" OR "iron-miner" OR labrador OR "bird fancer" OR indium OR "bird breeder" OR "pigeon keeper" OR "cheese washer") NEAR/2 lung*):ti,ab 7

#114 ((woodworker* OR "coal miners" OR "iron miners" OR "iron-miners" OR "bird fancers" OR "bird breeders" OR "pigeon keepers" OR "cheese washers" OR "peat mosses" OR "trombone Players" OR "Brass Players" OR "Wine-growers") NEAR/2 lung*):ti,ab 21

#115 (Asbestosis OR silicosis OR anthracosis OR Aluminosis OR siderosis OR Berylliosis OR Byssinosis OR Chalicosis OR Silicosiderosis OR Stannosis OR Talcosis OR Baritosis OR bagassosis OR Lycoperdonosis OR Sequoiosis OR Suberosis):ti,ab 153

#116 ((Bauxite OR beryllium) NEAR/2 (disease* OR disorder* OR poisoning OR syndrome* OR fibrosis)):ti,ab 12

#117 (pulmonary NEAR/2 (haemosiderosis OR haemo-siderosis)):ti,ab 0

#118 ((Hypersensitivity OR "hyper-sensitivity") NEAR/2 pneumonitis):ti,ab 5667

#119 "extrinsic allergic alveolitis":ti,ab 1

#120 ((Cephalosporium OR Isocyanate OR "Trimellitic anhydride" OR TMA OR Familial OR Domestic OR "Japanese summer" OR Metalwork* OR "Metal-work" OR "Metal-worker" OR "Metal-workers" OR Mollusc OR shell OR Sauna* OR worker* OR Streptomyces OR "Tap water") NEAR/2 HP):ti,ab 0

#121 "Fibrosing alveolitis":ti,ab 19

#122 ((alveolar OR lung* OR interstitial OR pulmonary) NEAR/3 fibrosis):ti,ab 2659

#123 #94 OR #95 OR #96 OR #97 OR #98 OR #99 OR #100 OR #101 OR #102 OR #103 OR #104 OR #105 OR #106 OR #107 OR #108 OR #109 OR #110 OR #111 OR #112 OR #115 OR #116 OR #117 OR #118 OR #119 OR #120 OR #121 OR #122 15719

**#124 #90 and #93 and #123 with Publication Year from 2010 to 2023, in Trials 250**

Humanistic burden terms based on:

Clayton S, Bambra C, Gosling R, Povall S, Misso K, Whitehead M. Assembling the evidence jigsaw: insights from a systematic review of UK studies of individual-focused return to work initiatives for disabled and long-term ill people [Internet]. BMC Public Health 2011 [cited 23.3.11];11:170.

Cost-effectiveness filter based on:

Centre for Reviews and Dissemination. Search strategies: NHS EED EMBASE using OvidSP (economics filter) [Internet]. York: Centre for Reviews and Dissemination; 2014 [accessed 2.6.14]. Available from:

http://www.crd.york.ac.uk/crdweb/searchstrategies.asp#nhseedembase

HRQoL free-text terms based on:

Figure 4: Common free-text terms for electronic database searching for HSUVs in Papaioannou D, Brazier JE, Paisley S. NICE DSU Technical Support Document 9: the identification, review and synthesis of health state utility values from the literature (Internet), 2011 (accessed: 18.8.11) Available from: http://www.nicedsu.org.uk

Arber M, Garcia S, Veale T, Edwards M, Shaw A, Glanville JM. Performance of Ovid Medline search filters to identify health state utility studies. Int J Technol Assess Health Care 2017;33(4):472-80.

**PubMed (NLM) (Internet): 2010-2023/10/04**

**Searched 4.10.23**

**32 #27 NOT #30 1,041 (limited 2010-2022/09/01)**

31 #27 NOT #30 1,096

30 #29 NOT (#29 AND #28) 3,736,037

29 rat[tiab] OR rats[tiab] OR mouse[tiab] OR mice[tiab] OR murine[tiab] OR rodent[tiab] OR rodents[tiab] OR hamster[tiab] OR hamsters[tiab] OR pig[tiab] OR pigs[tiab] OR porcine[tiab] OR rabbit[tiab] OR rabbits[tiab] OR animal[tiab] OR animals[tiab] OR dogs[tiab] OR dog[tiab] OR cats[tiab] OR cow[tiab] OR bovine[tiab] OR sheep[tiab] OR ovine[tiab] OR monkey[tiab] OR monkeys[tiab] 4,679,560

28 Human[tiab] OR humans[tiab] 3,221,614

27 #5 AND #26 1,144

26 #6 OR #7 OR #8 OR #9 OR #10 OR #11 OR #12 OR #14 OR #15 OR #16 OR #17 OR #19 OR #20 OR #21 OR #22 OR #23 OR #24 OR #25 698,931

25 "alveolar fibrosis"[Title/Abstract] OR "lung fibrosis"[Title/Abstract] OR "lungs fibrosis"[Title/Abstract] OR "interstitial fibrosis"[Title/Abstract] OR "pulmonary fibrosis"[Title/Abstract] 38,795

24 "Cephalosporium HP"[Title/Abstract] OR "Isocyanate HP"[Title/Abstract] OR "Trimellitic anhydride HP"[Title/Abstract] OR "TMA HP"[Title/Abstract] OR "Familial HP"[Title/Abstract] OR "Domestic HP"[Title/Abstract] OR "Japanese summer HP"[Title/Abstract] OR "Metalwork HP"[Title/Abstract] OR "Metal-worker HP"[Title/Abstract] OR "Metalworker HP"[Title/Abstract] OR "Metal-workers HP"[Title/Abstract] OR "Metal-work HP"[Title/Abstract] OR "Mollusc HP"[Title/Abstract] OR "shell HP"[Title/Abstract] OR "Sauna HP"[Title/Abstract] OR "Saunas HP"[Title/Abstract] OR "worker HP"[Title/Abstract] OR "workers HP"[Title/Abstract] OR "Streptomyces HP"[Title/Abstract] OR "Tap water HP"[Title/Abstract] OR "Fibrosing alveolitis"[Title/Abstract] 905

23 "Hypersensitivity pneumonitis"[Title/Abstract] OR "hyper-sensitivity pneumonitis"[Title/Abstract] OR "extrinsic allergic alveolitis"[Title/Abstract] 3,492

22 "pulmonary haemosiderosis"[Title/Abstract] OR "pulmonary haemo-siderosis"[Title/Abstract] OR "pulmonary hemosiderosis"[Title/Abstract] OR "pulmonary hemo-siderosis"[Title/Abstract] 959

21 (Bauxite[Title/Abstract] OR beryllium[Title/Abstract]) AND (syndrome[Title/Abstract] OR disease[Title/Abstract] OR disorder[Title/Abstract] OR syndromes[Title/Abstract] OR diseases[Title/Abstract] OR disorders[Title/Abstract] OR poisoning[Title/Abstract] OR fibrosis[Title/Abstract]) 884

20 Asbestosis[Title/Abstract] OR silicosis[Title/Abstract] OR anthracosis[Title/Abstract] OR Aluminosis[Title/Abstract] OR siderosis[Title/Abstract] OR Berylliosis[Title/Abstract] OR Byssinosis[Title/Abstract] OR Chalicosis[Title/Abstract] OR Silicosiderosis[Title/Abstract] OR Stannosis[Title/Abstract] OR Talcosis[Title/Abstract] OR Baritosis[Title/Abstract] OR bagassosis[Title/Abstract] OR Lycoperdonosis[Title/Abstract] OR Sequoiosis[Title/Abstract] OR Suberosis[Title/Abstract] 12,656

19 ("coal miner"[Title/Abstract] OR coalworker[Title/Abstract] OR worker[Title/Abstract] OR "coal miners"[Title/Abstract] OR coalworkers[Title/Abstract] OR workers[Title/Abstract] OR black[Title/Abstract] OR aluminium[Title/Abstract] OR "iron miner"[Title/Abstract] OR "iron-miner"[Title/Abstract] OR "iron miners"[Title/Abstract] OR "iron-miners"[Title/Abstract] OR miner[Title/Abstract] OR miners[Title/Abstract] OR labrador[Title/Abstract] OR "bird fancier"[Title/Abstract] OR "bird fanciers"[Title/Abstract] OR indium[Title/Abstract] OR "bird breeder"[Title/Abstract] OR "pigeon keeper"[Title/Abstract] OR "cheese washer"[Title/Abstract] OR "bird breeders"[Title/Abstract] OR "pigeon keepers"[Title/Abstract] OR "cheese washers"[Title/Abstract] OR compost[Title/Abstract] OR farmer[Title/Abstract] OR farmers[Title/Abstract] OR "hot tub"[Title/Abstract] OR humidifier[Title/Abstract] OR humidifiers[Title/Abstract] OR "Japanese summer house"[Title/Abstract] OR "maple bark"[Title/Abstract] OR miller[Title/Abstract] OR "peat moss"[Title/Abstract] OR millers[Title/Abstract] OR snuff[Title/Abstract] OR "trombone Player"[Title/Abstract] OR "Brass Player"[Title/Abstract] OR "trombone Players"[Title/Abstract] OR "Brass Players"[Title/Abstract] OR "Wine-grower"[Title/Abstract] OR woodworker[Title/Abstract] OR woodworkers[Title/Abstract]) OR (lung[Title/Abstract] AND lungs[Title/Abstract]) 553,525

18 ("thatched roof"[Title/Abstract] OR "maple bark"[Title/Abstract] OR "detergent worker"[Title/Abstract] OR welder[Title/Abstract] OR "detergent workers"[Title/Abstract] OR welders[Title/Abstract]) AND (syndrome[Title/Abstract] OR disease[Title/Abstract] OR disorder[Title/Abstract] OR syndromes[Title/Abstract] OR diseases[Title/Abstract] OR disorders[Title/Abstract]) 489

17 ("Cryptogenic organizing"[Title/Abstract] OR "Cryptogenic organizing"[Title/Abstract] OR "bronchiolitis obliterans"[Title/Abstract]) AND pneumonia[Title/Abstract] 1,637

16 "lung coniosis"[Title/Abstract] OR pneumoconiosis[Title/Abstract] OR pneumoconiotic[Title/Abstract] OR pneumokoniosis[Title/Abstract] OR pneumonoconiosis[Title/Abstract] OR pneumonokoniosis[Title/Abstract] OR silicoarthritis[Title/Abstract] 5,690

15 wegener[Title/Abstract] AND (syndrome[Title/Abstract] OR disease[Title/Abstract] OR disorder[Title/Abstract] OR syndromes[Title/Abstract] OR diseases[Title/Abstract] OR disorders[Title/Abstract] OR morbus[Title/Abstract] OR granuloma[Title/Abstract] OR churg[Title/Abstract] OR klinger[Title/Abstract]) 4,118

14 (wegener[Title/Abstract] OR polyangiitis[Title/Abstract] OR necrotising[Title/Abstract] OR respiratory[Title/Abstract] OR necrotizing[Title/Abstract] OR pneumogenic[Title/Abstract]) AND granulomatosis[Title/Abstract] 9,674

13 eosinophil[Title/Abstract] AND (pleurisy[Title/Abstract] OR pleuritis[Title/Abstract] OR lung[Title/Abstract] OR lungs[Title/Abstract] OR loeffler[Title/Abstract] OR Loffler[Title/Abstract] OR pneumonia[Title/Abstract] OR infiltrate[Title/Abstract] OR pulmonary[Title/Abstract]) 6,575

12 (carrington[Title/Abstract] OR Loeffler[Title/Abstract] OR Loffler[Title/Abstract] OR caplan[Title/Abstract] OR Hamman-Rich[Title/Abstract] OR Antisynthetase[Title/Abstract] OR "Anti-synthetase"[Title/Abstract]) AND (disease[Title/Abstract] OR disorder[Title/Abstract] OR syndrome[Title/Abstract] OR diseases[Title/Abstract] OR disorders[Title/Abstract] OR syndromes[Title/Abstract]) 2,158

11 ("chronic fibrous pneumonia"[Title/Abstract] OR "fibroid phthisis"[Title/Abstract] OR "phthisis fibroidea"[Title/Abstract] OR "pneumonia chronica fibrosa"[Title/Abstract] OR "pneumonia fibrosa chronica"[Title/Abstract] OR "pneumonia interstitialis"[Title/Abstract] OR "pneumonitis acuta"[Title/Abstract]) 0

10 ("chronic fibrous pneumonia"[Title/Abstract] OR "fibroid phthisis"[Title/Abstract] OR "phthisis fibroidea"[Title/Abstract] OR "pneumonia chronica fibrosa"[Title/Abstract] OR "pneumonia fibrosa chronica"[Title/Abstract] OR "pneumonia interstitialis"[Title/Abstract] OR "pneumonitis acuta"[Title/Abstract]) - Schema: all 0

9 "Interstitial Pneumonia"[Title/Abstract] OR "Interstitial Pneumonitides"[Title/Abstract] OR "Interstitial Pneumonitis"[Title/Abstract] OR "Interstitial pneumopathy"[Title/Abstract] OR "Interstitial pneumocystic"[Title/Abstract] OR "Interstitial pneumopathies"[Title/Abstract] OR "Interstitial Pneumonias"[Title/Abstract] 12,138

8 "Interstitial pulmonary"[Title/Abstract] OR "diffuse parenchymal pulmonary"[Title/Abstract] 1,644

7 ILD[Title/Abstract] OR ILDs[Title/Abstract] OR DPLD[Title/Abstract] OR IPD[Title/Abstract] OR IIP[Title/Abstract] OR IPF[Title/Abstract] OR DIP[Title/Abstract] OR AIP[Title/Abstract] OR NSIP[Title/Abstract] OR RBILD[Title/Abstract] OR RB-ILD[Title/Abstract] OR COP[Title/Abstract] OR BOOP[Title/Abstract] OR CWP[Title/Abstract] 48,176

6 "Interstitial lung"[Text Word] OR "diffuse parenchymal lung"[Text Word] OR "Pneumoconiosis"[Mesh] OR "Bird Fancier's Lung"[Mesh:NoExp] OR "Asthma, Occupational"[Mesh:NoExp] OR "Agricultural Workers' Diseases"[Mesh] OR "Lung Diseases, Interstitial"[Mesh] 98,555

5 #3 AND #4 11,680

4 pubstatusaheadofprint OR publisher[sb] OR pubmednotmedline[sb] 5,488,259

3 #1 OR #2 76,402

2 cough[Text Word] OR coughs[Text Word] OR coughing[Text Word] OR coughed[Text Word] 76,402

1 "Cough"[Mesh:NoExp] 18,729

**Europe PMC, including MedRxiv and bioRxiv preprints (Internet): 2010-2023/10/04**

**Searched 4.10.23**

[**https://europepmc.org/advancesearch**](https://europepmc.org/advancesearch)

**Limited 2010-2023**

| **Search terms (limited to preprints only)**  **Simple search** | **Results** |
| --- | --- |
| (TITLE:"cough" OR TITLE:"coughs" OR TITLE:"coughed" OR TITLE:"coughing")  AND  ("cost" OR "costs" OR "economics" OR "burden" OR "caregiver" OR "caregivers" OR "isolation" OR "quality of life" OR "qol" OR "hrqol" OR "hrql" OR "survey" OR "questionnaire" OR "impact" OR "productivity" OR "employment" OR "social" OR "benefit" OR "pension" OR "costing" OR "costly" OR "price" OR "expenditure" OR "disability" OR "health status" OR "adjusted life" OR "utility" OR "utilities" OR "surveys" OR "questionnaires" OR "interview" OR "interviews")  **All fields** | 126 |
| (TITLE:"cough" OR TITLE:"coughs" OR TITLE:"coughed" OR TITLE:"coughing")  AND  ("interstitial" OR "ILD" OR "ilds" OR "DPLD" OR "IPD" OR "IIP" OR "IPF" OR "DIP" OR "AIP" OR "NSIP" OR "RBILD" OR "RB-ILD" OR "COP" OR "BOOP" OR "CWP" OR "fibrosis" OR "diffuse parenchymal pulmonary" OR "chronic fibrous pneumonia" OR "fibroid phthisis" OR "phthisis fibroidea" OR "pneumonia chronica fibrosa" OR "pneumonia fibrosa chronica" OR "pneumonia interstitialis" OR "pneumonitis acuta" OR "eosinophil" OR "lung injury" OR "lung injuries")  **All fields** | 20 |
| (TITLE:"cough" OR TITLE:"coughs" OR TITLE:"coughed" OR TITLE:"coughing")  **All fields**  AND  ("wegener" OR "lung coniosis" OR "pneumoconiosis" OR "pneumoconiotic" OR "pneumokoniosis" OR "pneumonoconiosis" OR "pneumonokoniosis" OR "silicoarthritis" OR "crytogenic" OR "bronchiolitis obliterans" OR "miners lung" OR "coalminers lung" OR "pulmonary haemosiderosis" OR "pulmonary haemo-siderosis" OR "pulmonary hemosiderosis" OR "pulmonary hemo-siderosis")  **All fields** | 1 |
| (TITLE:"cough" OR TITLE:"coughs" OR TITLE:"coughed" OR TITLE:"coughing")  AND  ("Asbestosis" OR "silicosis" OR "anthracosis" OR "Aluminosis" OR "siderosis" OR "Berylliosis" OR "Byssinosis" OR "Chalicosis" OR "Silicosiderosis" OR "Stannosis" OR "Talcosis" OR "Baritosis" OR "bagassosis" OR "Lycoperdonosis" OR "Sequoiosis" OR "Suberosis" OR "Hypersensitivity pneumonitis" OR "hyper-sensitivity pneumonitis" OR "extrinsic allergic alveolitis" OR "alveolar fibrosis" OR "lung fibrosis" OR "lungs fibrosis" OR "interstitial fibrosis" OR "pulmonary fibrosis" OR "occupational lung" OR "occupational disease" OR "occupational diseases" OR "occupational disorder" OR "occupational disorders")  **All fields** | 7 |
| **Total (with duplicates)** | **154** |

**NHS Economic Evaluation Database (NHS EED) (CRD): 2010-2015/03/31**

**Searched 1.9.22**

**https://www.crd.york.ac.uk/CRDWeb/**

1 MeSH DESCRIPTOR Cough IN NHSEED 5

2 (cough*) IN NHSEED FROM 2010 TO 2015 23

**3 #1 OR #2 27**
