# Supplementary material for: The burden of cough in idiopathic pulmonary fibrosis and other interstitial lung diseases: a systematic evidence synthesis
Source: Respir Res. 2024 Aug 27;25:325. doi: 10.1186/s12931-024-02897-w (PMC11351049; doi:10.1186/s12931-024-02897-w)
Supplement: Supplementary file 2 — Supplementary Material 2 [file 12931_2024_2897_MOESM2_ESM.docx]

**The burden of cough in idiopathic pulmonary fibrosis and other interstitial lung diseases: a systematic evidence synthesis**

Rhiannon Green,^1^ Michael Baldwin,^2^ Nick Pooley,^1^ Kate Misso,^1^ Maureen PMH Rutten-van Mölken,^3^ Nina Patel,^4^ Marlies S Wijsenbeek^5^

^1^Market Access, Maverex Limited, Manchester, United Kingdom
^2^Value and Patient Access, Boehringer Ingelheim International GmbH, Ingelheim am Rhein, Germany
^3^Erasmus School of Health Policy and Management, Erasmus University Rotterdam, Rotterdam, The Netherlands
^4^Inflammation Medicine, Boehringer Ingelheim Pharmaceuticals Inc, Ridgefield, CT, USA
^5^Respiratory Medicine, Erasmus Medical Center, Rotterdam, The Netherlands

**Corresponding author**: Marlies S Wijsenbeek

**Address:** Erasmus MC: University Medical Center Rotterdam, Pulmonary Medicine, Gravendijkwal 230, Rotterdam 3015 CE, Netherlands

**Email:** [m.wijsenbeek-lourens@erasmusmc.nl](mailto:m.wijsenbeek-lourens@erasmusmc.nl)

**Supplementary Results**

[1. Bias assessment 2](#_Toc157076059)

[2. Studies in IPF 4](#_Toc157076060)

[Table 2.1 IPF Study Designs 4](#_Toc157076061)

[Table 2.2 IPF Patient Characteristics 6](#_Toc157076062)

[Table 2.3 IPF Outcomes 11](#_Toc157076063)

[3. Studies in Mixed ILDs 19](#_Toc157076064)

[Table 3.1 Mixed ILD Study Designs 19](#_Toc157076065)

[Table 3.2 Mixed ILD Patient Characteristics 21](#_Toc157076066)

[Table 3.3 Mixed ILD Patient Outcomes 25](#_Toc157076067)

[4. Studies in CTD-ILDs 30](#_Toc157076068)

[Table 4.1 CTD-ILD Study Designs 30](#_Toc157076069)

[Table 4.2 CTD-ILD Patient Characteristics 31](#_Toc157076070)

[Table 4.3 CTD-ILD Patient Outcomes 33](#_Toc157076071)

[5. Studies in Sarcoidosis 36](#_Toc157076072)

[Table 5.1 Sarcoidosis Study Designs 36](#_Toc157076073)

[Table 5.2 Sarcoidosis Patient Characteristics 37](#_Toc157076074)

[Table 5.3 Sarcoidosis Patient Outcomes 38](#_Toc157076075)

Bias assessment

Given the broad range of study types in this review, risk of bias assessment was done based on study design rather than the initially planned adapted Newcastle Ottawa Scale, as it would not have adequately captured the risk of bias in the different studies. The PROs investigated in this review were not always the main outcome assessed in these studies and cough was not always the main exposure. The risk of bias assessment tool used and outcome are summarised for the 57 unique studies assessed in Table 1 (studies with multiple publications summarised, four not assessed). 29 of the studies were deemed to be of low risk of bias whereas 28 were deemed to have some issues that could lead to bias.

For the 11 randomised controlled trials and seven validation studies the risk of bias was low, two of the crossover RCTs had some issues that may have biased their results, or there was an issue with their reporting. For the observational trials, the most common risk of bias issue was the sample size justification which may affect the generalizability of the study results and the lack of reporting on missing data and non-responders. Key 2010 for example is widely referenced in the literature, but only included 19 patients in the study. For the qualitative studies, the issues identified in the quality assessment included a lack of discussion of certain ethical issues and consideration of the relationship of the researcher and the study participants. While there were some concerns mentioned above, they did not relate to the analysis. Finally, there were four studies not assessed for risk of bias due to their different study designs (non-randomised interventional studies).

**Table 1.1** Risk of Bias summaries by assessment type

| Study | Study design | Assessment Tool | Risk-of-bias Judgement |
| --- | --- | --- | --- |
| Dutta 2019 | RCT | Cochrane ROB 2 - RCT | Low |
| Tashkin 2016, Volkmann 2020, Tashkin 2017 | RCT | Cochrane ROB 2 - RCT | Low |
| Volkmann 2022 | RCT | Cochrane ROB 2 - RCT | Low |
| Wilson 2021 | RCT | Cochrane ROB 2 - RCT | Low |
| Theodore 2012 | RCT | Cochrane ROB 2 - RCT | Low |
| Martinez 2022 | RCT | Cochrane ROB 2 - RCT | Low |
| Bassi 2021 | RCT | Cochrane ROB 2 - RCT | Low |
| Birring 2017 | Crossover RCT | Cochrane ROB 2 - Crossover | Low |
| Guler 2021 | Crossover RCT | Cochrane ROB 2 - Crossover | Some concerns |
| Martinez 2021 | Crossover RCT | Cochrane ROB 2 - Crossover | Some concerns |
| Horton 2012, Lechtzin 2013 | Crossover RCT | Cochrane ROB 2 - Crossover | Low |
| Algamdi 2019 | Observational | AXIS | Low |
| Gries 2013 | Observational | AXIS | Low |
| Lan 2021 | Observational | AXIS | Some concerns |
| Sato 2019 | Observational | AXIS | Some concerns |
| Sato 2021 | Observational | AXIS | Some concerns |
| Topcu 2021 | Observational | AXIS | Some concerns |
| Wuyts 2018, Wuyts 2022 | Observational | AXIS | Some concerns |
| Yount 2016 | Observational | AXIS | Some concerns |
| Yuan 2020 | Observational | AXIS | Some concerns |
| Cheng 2017 | Observational | AXIS | Low |
| Case 2020 | Observational | AXIS | Low |
| de Andrade 2021 | Observational | AXIS | Some concerns |
| Glaspole 2017 | Observational | AXIS | Some concerns |
| Gvozdenovic 2020 | Observational | AXIS | Low |
| Hollmen 2023 | Observational | AXIS | Some concerns |
| Judson 2017 | Observational | AXIS | Some concerns |
| Jones 2011 | Observational | AXIS | Some concerns |
| Key 2010 | Observational | AXIS | Some concerns |
| Kim 2022 | Observational | AXIS | Some concerns |
| Veit 2023 | Observational | AXIS | Some concerns |
| Paixao 2023 | Observational | AXIS | Some concerns |
| Park 2022 | Observational | AXIS | Some concerns |
| Prasad 2021 | Observational | AXIS | Some concerns |
| Saari 2023 | Observational | AXIS | Low |
| Saunders 2023 | Observational | AXIS | Some concerns |
| Scholand 2014 | Observational | AXIS | Some concerns |
| Sinha 2016 | Observational | AXIS | Some concerns |
| Tzouvelekis 2020 | Observational | AXIS | Low |
| Veit 2023 | Observational | AXIS | Some concerns |
| Bajwah 2013 | Qualitative | CASP | Low |
| Mann 2023 | Qualitative | CASP | Low |
| Duck 2012 | Qualitative | CASP | Low |
| Belkin 2014 | Qualitative | CASP | Low |
| Lindell 2017 | Qualitative | CASP | Low |
| Overgaard 2016 | Qualitative | CASP | Low |
| Mittoo 2015 | Qualitative | CASP | Some concerns |
| Swigris 2021 | Qualitative | CASP | Some concerns |
| Bacci 2018 | Qualitative | CASP | Some concerns |
| Boland 2016 | Qualitative | CASP | Some concerns |
| Fisher 2019 | Validation study | COSMIN | Low |
| Nagata 2012 | Validation study | COSMIN | Low |
| Kirsten 2022 | Validation study | COSMIN | Low |
| Pan 2019 | Validation study | COSMIN | Low |
| Swigris 2010 | Validation study | COSMIN | Low |
| Yates 2018 | Validation study | COSMIN | Low |
| Swigris 2018 | Validation study | COSMIN | Low |

Studies in IPF

## Table 2.1 IPF Study Designs

| Author year | Cough group | Location | Study type | Trial registration | Study duration | Setting |
| --- | --- | --- | --- | --- | --- | --- |
| Interventional trials | | | | | | |
| Dutta 2019 | Chronic cough | United Kingdom | Pilot trial | NCT02085018 | 18 months | Single centre |
| Lechtzin 2013/Horton 2012 | Chronic cough | United States | Crossover RCT | NCT00600028 | 6 months | Single centre |
| Martinez 2022 | Chronic cough | 11 countries (inc United Kingdom and United States) | RCT | NCT03864328 | 12 weeks | Multicentre |
| Guler 2021 | Chronic cough | Switzerland | Crossover RCT | NCT02173145 | 3 months | Multicentre |
| Martinez 2021 | Chronic cough | United States | Crossover RCT | NCT02502097 | 52 days | Multicentre |
| Birring 2017 | Chronic cough | United Kingdom and the Netherlands | Crossover RCT | NCT02412020 | 14 days | Multicentre |
| Lee 2023 | Chronic cough | Korea | Single arm trial | - | 1 year | Single centre |
| Wilson 2021 | Broader includes | United Kingdom | RCT | ISRCTN17464641 | 1 year | Multicentre |
| Jastrzębski 2023 | Broader includes | Poland | Single arm trial | - | 12 months | Multicentre |
| Observational studies | | | | | | |
| Saari 2023 | Chronic cough | Finland | Case-control study |  | 6 years | Multicentre |
| Yount 2016 | Majority Cough | United States | Cross sectional study | - | 7-10 days | NA |
| Glaspole 2017 | Majority Cough | Australia | Cohort study | Australian IPF Registry | 12 months | Multicentre |
| Wuyts 2018/ Wuyts 2022 | Majority Cough | Belgium and Luxembourg | Cohort study | PROOF Registry | 24 months | Multicentre |
| Key 2010 | Majority Cough | United Kingdom | Cohort study | - | NR | Single centre |
| Tzouvelekis 2020 | Minority cough | Greece | Cohort study | - | 1 year | Single centre |
| Scholand 2014 | Minority cough | United States | Genetic study | - | 1 year | Single centre |
| Saunders 2023 | Minority cough | United Kingdom | Prospective cohort | PROFILE Study | 3 years | Multicentre |
| Jones 2011 | Majority Cough | United Kingdom | Cross sectional study | - | NR | Single centre |
| Prasad 2021 | Broader includes | Australia | Cohort study | - | 12 month | Single centre |
| Park 2022 | Broader includes | Korea | Cohort study | - | 2 years | Single centre |
| de Andrade 2021 | Broader includes | United States | Cohort study | - | 3 years | Multicentre |
| Case 2020 | Broader includes | Puerto Rico, United States | Cohort study | IPF-PRO Registry/ NCT01915511 | 3 years | Multicentre |
| Kim 2022 | Broader includes | Puerto Rico, United States | Cohort study | IPF-PRO Registry/ NCT01915511 | 3 years | Multicentre |
| Hollmen 2023 | Broader includes | Belgium, Finland, France, the Netherlands, Ireland and the United Kingdom | Cross-sectional, online survey | - | 3 months | Multicentre |
| Validation studies |  | | | | | |
| Swigris 2010 | Broader includes | Unites States | Validation study |  | NR | Multicentre |
| Swigris 2018 | Broader includes | 24 countries | Validation study using pooled RCT data | NCT01335464 | 12 months | Multicentre |
| Mixed methods studies | | | | | | |
| Bacci 2018 | Majority Cough | United States | Cross-sectional, qualitative study | - | NR | Multicentre |
| Gries 2013 | Majority cough | United States | Mixed methods | - | NR | Multicentre |
| Igai 2022 | Broader includes | Japan | Mixed methods | UMIN000031861 | 4-8 weeks | Multicentre |
| Qualitative Studies | | | | | | |
| Lindell 2017 | Broader includes | Unites States | Qualitative | - | NR | Single centre |
| Belkin 2014 | Broader includes | United States | Qualitative | - | NR | Single centre |
| Duck 2012 | Broader includes | United Kingdom | Qualitative | - | Data collected in 2007, analysed in 2012 | Single centre |
| Overgaard 2016 | Broader includes | Denmark | Qualitative | - | NR | Multicentre |

NR, not reported; RCT, randomised controlled trial.

## Table 2.2 IPF Patient Characteristics

| Author year | N | Age, mean (SD)* | Female  (%) | Race (%) | Comorbidities | Treatment | Disease duration, mean (SD)* |
| --- | --- | --- | --- | --- | --- | --- | --- |
| Interventional trials | | | | | | | |
| Birring 2017 | IPF: 24 | 67 (range 56–79) | 38% | White - 92% | NR | PA101 | Duration of cough: 5.6 (4.2) years |
|  | CIC:27 | 62 (range 23–73) | 78% | White - 93% |  |  | Duration of cough: 9.9 (9.8) years |
| Dutta 2019 | 23 | 71.3 (6.7) | 17.4% | NR | NR | Omeprazole | NR |
|  | 22 | 71 (7.3) | 27.3% |  |  | Matched placebo |  |
| Lechtzin 2013/ Horton 2012 | 23 | 67.6 (7.8) | 22% | White: 91.2%,  Black 4.4%,  Hispanic 4.4% | GERD and chronic rhinitis | Thalidomide | 20.5 (3–59) months |
| Martinez 2022 | 29 | 71.3 (6.2) | 41.4% | White: 90%, | NR | RVT-1601 10mg | 4.0 (3.2) years |
|  | 25 | 69.4 (6.6) | 32%, | White: 92% |  | RVT-1601 40mg | 3.3 (3.3) years |
|  | 27 | 70.4 (6.6) | 51.9% | White: 100% |  | RVT-1601 80mg | 2.9 (2.4) years |
|  | 27 | 71.2 (7.3) | 25.9% | White: 100% |  | Placebo | 3.6 (3.7) years |
| Guler 2021 | 20 | 64 (7) | 80% | NR | Chronic rhinitis, Sinusitis, GERD, Cardiac disease, Pulmonary hypertension and Diabetes | Azithromycin | NR |
| Martinez 2021 | 51 | 69.6 (7.17) | 22% | White: 98%  Asian 2% | NR | Gefapixant | 7 (6.13) years |
| Lee 2023 | 30 | 73 (66.75–76) | 13.3% | NR | All had chronic bronchitis* | Pirfenidone treatment, main Synatura | NR |
| Wilson 2021 | 341 | 71.9 (7.8) | 18% | NR | COPD, Bronchiectasis, IHD, GORD, Diabetes, Osteoporosis, Pulmonary hypertension, Anxiety/depression | Co-trimoxazole | NR |
| Jastrzębski 2023 | 87 | 65 (8.63) | 27% | NR | Respiratory disorders other than IPF: 9 (17.3%)  COPD: 1 (1.9%)  Respiratory failure: 8 (15.4%)  GERD: 10 (19.2%)  Metabolism disorders: 19 (36.5%)  Diabetes mellitus: 10 (19.2%)  Hypercholesterolemia: 9 (17.3%)  Vascular disorders: 36 (69.2%)  Hypertension: 24 (46.2%)  Cardiac disorders: 19 (36.5%)  Osteoarthritis: 18 (34.6%)  Infections developed during the course of the study: 2 (3.9%)  Chronic sinusitis: 2 (3.9%)  Nervous system disorders: 3 (5.8%)  Psychiatric disorders: 2 (3.9%)  Depression: 10 (19.2%) | Pirfenidone treatment | NR |
| Observational studies | | | | | | | |
| Saari 2023 | IPF with chronic cough: 46 | 67.5 (64.7–70) | 56.5% | NR | GERD: 3 (6.5 %)  OSA: 2 (4.3 %)  Asthma: 6 (13.0 %)  COPD: 0 |  |  |
|  | IPF without chronic cough: 22 | 67 (61–75) | 13.6% | NR | NR |  |  |
|  | Chronic cough: 184 | 67.5 (64–70) | 56.5% | NR | NR |  |  |
| Glaspole 2017 | 516 | 71.3 (8.6) | 33% | White: 95.5% | Asthma, COPD, Heart disease, Pulmonary hypertension, Lung cancer | NR | NR |
| Yount 2016 | 220 | 61.0 (5.6) | 30% | White: 83%,  Black 7%,  Hispanic 12%,  Asian 7%,  American Indian/Alaska native 4%,  Other 4% | Dyspnoea | NR | NR |
| Wuyts 2018/ Wuyts 2022 | 277 | 69.6 (8.6) | 23% | White: 92.1% | Most common were GERD, hypertension and hypercholesterolaemia | Standard of care | 1284.1 days since diagnosis (3.5 years) |
| Key 2010 | 19 | 70.8 (8.6) | 26% | NR | NR | Steroid Use: 68% | 3 (1-6) years |
| Scholand 2014 | 68 | 74.41 (8.01) | 38% | NR | GERD, Sleep Apnoea, UACS | NR | NR |
| Saunders 2023 | 632 | 70.2 (8.3) | 22.9% | NR | Gastroesophageal reflux: 307 (48.6%) | PPI therapy: 176 (27.8%)  Angiotensin-converting enzyme inhibitor therapy: 107 (16.9%)  Antifibrotic therapy: 56 (8.8%) | NR |
| Tzouvelekis 2020 | 101 | 70.8 (8.1) | 21% | NR | Depression | Anti-fibrotic treatment | NR |
| Jones 2011 | IPF: 27 | 71.7 (7) | 37% | NR | NR | Corticosteroid use, 0 | NR |
|  | Healthy control: 30 | 65.6 (5.3) | 30% | NR | NR | Corticosteroid use, 30% | NR |
| Case 2020 | 662 | 70 (65–75) | 29% | White: 94.1% | NR | Nintedanib or pirfenidone 54% | NR |
| de Andrade 2021 | Implementation score ≤0.6: 360 | 70 (64, 76) | 28% | White: 94.9% | GERD: 52% | Proton pump inhibitor 45%, H2 blocker 8%, Pirfenidone 17% Nintedanib 19% | NR |
|  | Implementation score >0.6: 367 | 71 (66, 75) | 25% | White: 94.1% | GERD: 58% | Proton pump inhibitor 64%, H2 blocker 11%, Pirfenidone 43% Nintedanib 33% | NR |
| Kim 2022 | Total N: 934  (IPF: 815,  CPFE: 119) | 70 (65, 75) | 27% | White: 93.7% | GERD, CHD, AF, PH, CHF, OSA | Oral steroid 11.5%, Bronchodilator 27.6%, Pulmonary vasodilator 2.4%, Pirfenidone 31.3%, Nintedanib 22.6% | NR |
| Park 2022 | Without airway disease: 64 | 68.3 (0.94) | 25% | NR | COPD, Asthma, eosinophilic bronchitis, induced sputum eosinophilia, FENO, high, increasing IgE, Blood eosinophilia, MAST | NR | NR |
|  | With airway disease: 6 | 72 (3) | 66.7% | NR | NR | NR | NR |
| Prasad 2021 | 54 | 68 (7.5) | 32% | NR | Pulmonary hypertension, Systemic hypertension, Cardiovascular disease, Diabetes mellitus, GERD, OSA, Depression | Nintedanib 37%, Pirfenidone 22.2% | NR |
| Hollmen 2023 | 111 | 41–50: 0.9%  51–60: 11.7%  61–70: 44.1%  71–80: 35.1%  >81: 8.1% | 37.8% | White: 91.4%  Hispanic/Mediterranean: 2.5%  Mixed/Multiple Ethnic Groups: 3.7%  Other: 2.5% | NR | NR | Less than 5 years: 71.3%  Between 5 and 12 years: 22.4%  More than 12 years: 4.6% |
| Validation studies | | | | | | | |
| Swigris 2010 | 95 | 69.3 (7.6) | 18% | White: 94%,  Black 1%,  Other 5% | Emphysema (by HRCT), PH by echocardiogram, Stable CAD | Prednisone 24%, Azathioprine 14%, N-acetyl cysteine 24% | 2.9 (2.8) years |
| Swigris 2018 | 1061 | 66.8 (8.0) | 21% | White: 57.3% | Dyspnoea | BIBF 1120 | 1.6 (1.3) years |
| Mixed methods studies | | | | | | | |
| Bacci 2018 | Phase 1: 30 | 67.7 (8.4) Range: 50–88 | 40% | White: 93.3%,  Black: 3.3%,  Mexican American: 6.7% | NR | NR | 3.5 (2.0) years |
|  | Phase 2: 168 | 67.8 (6.62) | 23.8% | White: 69.6%  Asian: 14.3%  American Indian or Alaska Native: 3.6%  Other: 12.5% | NR | NR | NR |
| Gries 2013 | 18 | 68.9 (11.9) | 22% | White: 88.9%,  Black 5.6%,  Other 5.6% | Dyspnoea | NA | 2.4 (1.6) years |
| Igai 2022 | 12 | 77.3 (4.6) | 8% | NR | NR | Care programme intervention | 6.7 (4) years |
| Qualitative studies | | | | | | | |
| Lindell 2017 | 13 | 71.4 (7.2) | 0% | White: 100% | NR | NR | 1 to 11 years |
| Belkin 2014 | 28 | 67 (7) | 93% | NR | NR | NR | 4 (3) years |
| Duck 2012 | 17 | 67 | 59% | NR | NR | NR | NR |
| Overgaard 2016 | 25 | 71.1 (50–91) | NR | NR | NR | 15 were treated with pirfenidone | NR |

Mean (SD) unless presented as median (IQR)

*Chronic bronchitis was defined as cough and sputum for more than 3 months in 2 consecutive years

ACE, **angiotensin-converting enzyme**; AF, atrial fibrillation; CAD, coronary artery disease; CHD, chronic heart disease; CHF, congestive heart failure; CIC, idiopathic chronic cough; COPD, chronic obstructive pulmonary disease; GERD/GORD, gastroesophageal reflux disease; IHD, ischaemic heart disease; ILD, interstitial lung disease; IPF, idiopathic pulmonary fibrosis; NR, not reported; OSA, obstructive sleep apnoea; PH, pulmonary hypertension; UACS, upper airway cough syndrome.

## Table 2.3 IPF Outcomes

| Author year | Arm | Disease severity (FVC % pred.) | Cough severity measures | HRQoL/impact measures | Group comparisons | Burden of cough |
| --- | --- | --- | --- | --- | --- | --- |
| Interventional trials | |  |  |  |  |  |
| Birring 2017 | IPF | 73 (15) | LCM 24-hr cough frequency: NR  VAS: 61.5 (13.0) | LCQ: 12.9 (3.2) | LCQ scores were lower in CIC patients, and the response of IPF to treatment suggests the mechanism of cough may be disease specific | Significant correlation between daytime cough frequency, VAS cough severity (r=0.683) and LCQ (r=–0.682). |
|  |  |  |  | KBILD: 56.2 (10.5) |  |  |
|  | CIC | - | LCM 24-hr cough frequency: NR  VAS: 70.5 (15.3) | LCQ: 10.5 (15.3)  KBILD: NA |  |  |
| Dutta 2019 | Omeprazole | 73.1 (17.1) | 24 hr cough monitor: 9.6/hr (4.2–18.3) | LCQ: 15.3 (3.3)  Physical: 5.1 (1.1) Physiological: 5.0 (1.4) Social: 5.2 (1.1) | There was a greater reduction in 24-hr cough frequency in the omeprazole group compared with placebo | Change in objective cough not reflected in subjective measures |
|  |  |  |  |  |  |  |
|  | Placebo | 77.9 (17.6) | 24 hr cough monitor:  8.9/hr (6.8–12.8) | LCQ: 15.1 (3.2)  Physical: 5.2 (1) Physiological: 4.8 (1.2) Social: 5.1 (1.3) |  |  |
|  |  |  |  |  |  |  |
| Lechtzin 2013/ Horton 2012 | - | 70.4 (13.7) | VAS: 64.8 (21.4) | CQLQ: 60.5 (12) | - | Cough VAS correlated with SGRQ and physical and functional CQLQ, cough aetiology discussed |
|  |  |  |  | SGRQ: 57.4 (18.8) |  |  |
| Martinez 2021 | Gefapixant | All patients  Ratio: 83.6 (10.60) | LCM mean cough count: 46.2 (43.06) | CQLQ: 56.5 (3.26) | - | Reductions in cough severity measures were not reflected in cough related quality of life |
|  |  |  | VAS: 56.0 (24.03) | Cough Severity Diary: 4.5 (1.75) |  |  |
|  | Placebo |  | LCM mean cough count: 48 (55.17) | CQLQ: 56.8 (11.25) |  |  |
|  |  |  | VAS: 53.9 (22.8) | Cough Severity Diary: 4.1 (2.04) |  |  |
| Martinez 2022 | RVT-1601 10 mg | 66.4 (16.8) | 24-hr cough count: 38.6./hr (23.3) | LCQ: 11.4 (3.90) |  | There were no significant differences in LCQ or cough counts between treatment groups |
|  |  |  | VAS: 71.9 (13.3) |  |  |  |
|  | RVT-1601 40 mg | 68.0 (14.2) | 24-hr cough count: 38.7/hr (22.0) | LCQ: 11.1 (3.54) |  |  |
|  |  |  | VAS: 73.7 (15.67) |  |  |  |
|  | RVT-1601 80 mg | 69.2 (17.5) | 24-hr cough count: 37.1/hr (19.5) | LCQ: 11.0 (3.53) |  |  |
|  |  |  | VAS: 69.8 (12.34) |  |  |  |
|  | Placebo | 71.7 (18.7) | 24-hr cough count: 36.6/hr (20.3) | LCQ: 11.6 (3.40) |  |  |
|  |  |  | VAS: 69.2 (16.29) |  |  |  |
| Lee 2023 | - | 77.50 (67.75–92.50) | – | LCQ: 16.77 (I15.63–19.13)  Physical: 5.44 (4.88–6)  Psychological: 5.57 (4.96–6.61)  Social: 6 (5.5–6.75) | 33.3% of patients achieved MCID after treatment (median difference of total LCQ score: 2.38, range: 1.79–3.32) | There was no significant improvement in cough related QoL (median difference 0.12, range: -0.73 to 0.97, P = .772) or respiratory related QoL measurements. |
|  |  |  |  | SGRQ: 30.59 (19.41–37.82)  Symptoms: 46.44 (35.08–55.96)  Activity: 45.1 (28.52–61.34)  Impact: 13.55 (6.08–23.78) |  |  |
| Guler 2021 | - | 66 (17) | VAS: 5.6 (2.3)* | SGRQ: 57.2 (18.6) | - | LCQ correlated negatively with VAS severity (r=–0.42) and VAS correlated moderately with SGRQ (r=0.42) |
|  |  |  | Cough index /h (n=15)  Wake: 6.2 (4.4-8.2)  Sleep: 0.6 (0.1-1.1)  Cough Attack Index /h (n=15)  Wake: 0.9 (0.7-1.3)  Sleep: 0.1 (0-0.4) | LCQ: 11.7 (3.7)  Physical: 4.3 (1.1)  Physiological: 3.6 (1.4)  Social: 3.8 (1.5) |  |  |
| Wilson 2021 | - | 56.2 (8.9) | VAS: 44.7 (27.0) | LCQ: 16.08 (3.55) | - | Cough correlation with QoL not reported, after 18 months the co-trimoxazole group had better VAS cough but not LCQ or KBILD |
|  |  |  |  | KBILD: 53.7 (9.71) |  |  |
|  |  |  |  |  |  |  |
| Jastrzębski 2023 | - | 82.02 (17.65) |  | LCQ: 14.47 (3.74) | - | After 12 months of pirfenidone treatment, 12% of patients experienced improvements in their QoL and cough and dyspnoea reduction but better mean LCQ was not statistically significant (14.47 ± 3.74 vs. 15.24 ± 3.61; p = 0.26) |
|  |  |  |  | SGRQ: 46.69 (20.62)  Symptoms: 52.04 (21.24)  Activity: 55.63 (23.31)  Impact: 39.54 (23.95) |  |  |
|  |  |  |  | SF-36 PCS: 42.33 (8.73)  SF-36 MCS: 44.60 (11.85) |  |  |
| Observational studies | |  |  |  |  |  |
| Saari 2023 | IPF with chronic cough | 78.50 (73.7–82.3) | Cough response to paint or fumes: 5.5 (5–7)  Cough bout frequency per day: 4 (3–6) | Cough disturbing sleep: 5.5 (4–6)  LCQ: 14.8 (11.5–18.1)  Physical: 4.9 (3.9–6.1)  Psychological: 4.6 (3.7–5.9)  Social: 5.5 (3.7–6.5) | IPF patients with chronic cough had significantly lower LCQ scores than those without. Scores for cough disturbing sleep and cough bout frequency per day were also lower.  There were no significant differences between the IPF chronic cough and community-based chronic cough groups in LCQ total scores and the LCQ physical, psychological, and social impact scores or individual LCQ question scores | The prevalence of chronic cough was 68% among patients with IPF.  The results suggested that in early stage IPF disease, cough is undistinguishable from a community-based chronic cough |
|  |  |  |  |  |  |  |
|  | IPF without chronic cough | 84.5 (77.0–91.25) | Cough response to paint or fumes: 6 (6–7)  Cough bout frequency per day: 6 (5.75–7) | Cough disturbing sleep: 7 (6–7) |  |  |
|  |  |  |  | LCQ: 18.2 (16.4–19.4)  Physical: 5.9 (5.1–6.4)  Psychological: 6 (5–6.7)  Social: 6.4 (5.8–6.8) |  |  |
|  | Chronic cough | NR | Cough response to paint or fumes: 6 (4–7)  Cough bout frequency per day: 5 (4–5) | Cough disturbing sleep: 6 (4–6) |  |  |
|  |  |  |  | LCQ: 15.4 (13–17.5)  Physical: 5.1 (4.5–5.6)  Psychological: 4.7 (3.9–5.7)  Social: 5.5 (4.5–6.3) |  |  |
| Glaspole 2017 | - | 81.0 (22.5) | VAS: 40.3 (21–56) | SGRQ: 46.6 (20.9) | - | Cough was an independent predictor of QoL |
|  |  |  |  | HADS-A: 4 (2–7)  HADS-D: 4 (2–7) |  |  |
| Jones 2011 | IPF | 80.4 (20.9) | VAS: 38 (15–60) | LCQ: 15.9 (11.9-19.5) | IPF patients had significantly higher median cough symptom scores than healthy controls | Subjective cough measures showed strong correlation |
|  |  |  | CSS: 4 (2–6) |  |  |  |
|  | Healthy control | 120.6 (13.7) | VAS: 0 (0–4.5) | LCQ: 20.8 (20.5-21) |  |  |
|  |  |  | CSS: 0 (0–0) |  |  |  |
| Wuyts 2018/ Wuyts 2022 | - | 80.6  (19.9) | VAS: 30.5 (25.2)  Median (IQR): 24.0 (9.0–50.0) Minimum–maximum: 0.0–100.0 | SGRQ: 47.0 (20.2) | - | SGRQ total scores and SGRQ impact scores remained stable over time, but cough VAS, SGRQ activity scores and SGRQ symptom scores increased in all patients;  Cough was not significantly associated with mortality |
|  |  |  |  | EQ-5D VAS: 61.1 (19.2) |  |  |
|  |  |  |  | Current health perception poor/very poor: 15.9% |  |  |
| Key 2010 | - | 78.5 (24.4) | Median 24-hr cough rate 9.4/h (1.5–39.4) | LCQ: 15.4 (6.95–20.88)  Physical: 5.13 (2.38–6.63)  Psychological: 5.29 (1.57–7) Social: 5.75 (2.25–7) | In *post-hoc* analyses cough rates in IPF were higher than healthy volunteers and patients with asthma and similar to patients with chronic cough | Strong correlations between objective cough frequency and cough VAS and cough-related QoL |
|  |  |  | VAS: 32 (2–77) |  |  |  |
| Yount 2016 | - | - | - | ATAQ-IPF cough scale: 23.6 (5.8)  FACIT: 2.5 (1.2) | - | Cough was significantly associated with worse QoL |
| Tzouvelekis 2020 | - | 77.0 (21.2) | - | LCQ: 107.6 (30.2)** | - | Depression severity had a significant association with cough |
|  |  |  |  | BDI-II: 13.7 (8.4) |  |  |
|  |  |  |  | KBILD: 69.3 (18.7) |  |  |
|  |  |  |  | SGRQ: 39.7 (23.7) |  |  |
| Scholand 2014 | - | 72.57 (20.43) | - | LCQ: 16.16 (3.66) | - | Results suggested a genetic component to cough burden in IPF |
| Saunders 2023 | - | 74.1 (17.6) | - | LCQ: 16.1 (IQR 6.5),  Physical: 5.1 (IQR 1.7) Psychological: 5 (IQR 2.4)  Social: 5 (IQR 2.5) | There was no significant difference in survival between those with mild (LCQ >14), moderate (LCQ >10–<14) or worst cough (LCQ <10). Patients with progressive disease experienced worsening cough-related QoL with a 12-month change in LCQ score of -2.2 (±5.0) for the comparison between stable and progressive groups (P<0.001) | Greater cough burden was not associated with worse survival when corrected for age, gender, baseline lung function, and smoking history (HR, 1.01; 95% CI = 0.97–1.03, P= 0.34)  Longitudinal assessment of LCQ data suggested that the impact of cough- related QoL changes little over time for the majority of patients with IPF. |
| Prasad 2021 | - | 69.9 (16.7) | - | LCQ: 14.46 (0.71)  Physical: 4.77 (0.21)  Psychological: 4.83 (0.25)  Social: 4.86 (0.25) | - | LCQ not associated with physical activity decline |
|  |  |  |  | SGRQ: 47.2 (2.84) |  |  |
|  |  |  |  | HADS-A: 6.11 (0.62)  HADS-D: 5.74 (0.59) |  |  |
| Park 2022 | Without airway disease | 77.47 (1.9) | VAS: 3.31 (0.36) | CQLQ: 47 (1.66) | No significant difference in baseline CAT, CQLQ and SGRQ scores between groups | Respiratory and cough related QoL declined more in patients with IPF and airway disease than IPF alone |
|  |  |  |  | SGRQ: 29.09 (2.65) |  |  |
|  |  |  |  | CAT: 14.78 (1.14) |  |  |
|  |  |  |  | EQ-5D index: 0.85 (0.072) |  |  |
|  | With airway disease | 75 (5.03) | VAS: 4.33 (1.2) | CQLQ: 49.83 (8.6) |  |  |
|  |  |  |  | SGRQ: 33.16 (9.05) |  |  |
|  |  |  |  | CAT: 13.5 (13.9) |  |  |
|  |  |  |  | EQ-5D index: 0.744 (0.064) |  |  |
| de Andrade 2021 | Implementation score ≤0.6 | 71.9 (62.7–82.1) | - | CASA-Q  Impact: 81.3 (59.4–96.9) Symptom: 58.3 (41.7–83.3) | There were no associations between the implementation score and patient-reported outcomes except a trend in SGRQ impact domain score | Cough burden not associated with guideline implementation, correlation with HRQoL was not presented |
|  |  |  |  | SGRQ: 33.2 (19.4–47.8) |  |  |
|  |  |  |  | EQ-5D index: 0.8 (0.7–1.0)  EQ-5D VAS: 80 (70–90) |  |  |
|  |  |  |  | SF-36  Physical: 41.8 (34–50)  Mental: 54.3 (45.9–59.7) |  |  |
|  | Implementation score >0.6 | 68.2 (57.2– 79.9) | - | CASA-Q  Impact: 81.3 (59.4–96.9) Symptom: 62.5 (41.7–75.0) |  |  |
|  |  |  |  | SGRQ:40.6 (29.4–53.3) |  |  |
|  |  |  |  | EQ-5D index: 0.8 (0.7–1.0)  EQ-5D VAS: 75 (60–85) |  |  |
|  |  |  |  | SF-36  Physical: 37.9 (32.1–44.4)  Mental: 54.1 (46–59.9) |  |  |
| Case 2020 | - | NR | - | CASA-Q  Symptoms: 58.3 (41.7–75) Impact: 78.1 (56.3–93.8) | - | CASA-Q was not associated with death or lung transplant after adjustment |
|  |  |  |  | SGRQ: 39.5 (25.8–52.9) |  |  |
|  |  |  |  | SF-36  Physical: 39.2 (31.4–46.6)  Mental: 54.1 (46.5–58.8) |  |  |
|  |  |  |  | EQ-5D index: 0.8 (0.7–1.0) |  |  |
| Kim 2022 | CPFE | 71.8 (63.4– 90.8) | - | CASA-Q  Symptoms: 66.7 (50.0–83.3)  Impact: 87.5 (68.8–96.9) | The effect of cough on HRQoL was significantly lower in CPFE than IPF as measured by CASA-Q. The only other significant difference was SGRQ activity domain with worse scores in CPFE | The reasons for the lower impact of cough in patients with CPFE are  unclear, but the more frequent use of inhaler therapy and systemic corticosteroids may be partly responsible |
|  |  |  |  | SGRQ: 41.8 (27.3–53.7) |  |  |
|  |  |  |  | EQ-5D index: 0.8 (0.7–0.9)  EQ-5D VAS: 70 (60–85) |  |  |
|  |  |  |  | SF-12  Physical: 37.5 (32.2–43.6)  Mental: 53.3 (45.1–60.2) |  |  |
|  | IPF | 69.4 (58.8– 79.0) | - | CASA-Q  Symptoms: 58.3 (41.7–75)  Impact: 75 (56.3–93.8) |  |  |
|  |  |  |  | SGRQ: 39.5 (25.1–53.7) |  |  |
|  |  |  |  | EQ-5D index: 0.8 (0.7–1.0)  EQ-5D VAS: 75 (61.0–85) |  |  |
|  |  |  |  | SF-12  Physical: 39.4 (31.1–46.6)  Mental: 54.1 (45.8–59.2) |  |  |
| Hollmen 2023 |  | NR | - | I-Prefer questionnaire | Cough was more likely to prevent men, those on pirfenidone (vs nintedanib) and severe patients from doings things (p<0.001) | Around 30% of the patients restricted their time outside to 1–3 hours; 58% restricted outdoor time to under 1 hour. Coughing prevented around 60% of the patients from their daily activities (Shortness of breath prevented around 80%) |
| Validation studies | |  |  |  |  |  |
| Swigris 2010 | - | NR | - | ATAQ-IPF scores: 210 (46)  ATAQ-IPF cough: 16 (7) | - | Cough domain included in the QoL tool |
| Swigris 2018 | - | 79.6 (17.8) | - | CASA-Q  Symptoms: 59.9 (23.2)  Impact: 75.2 (23) | - | CASA-Q had moderate correlation with SGRQ domains |
|  |  |  |  | SGRQ: 39.5 (18.9) |  |  |
| Mixed methods studies | |  |  |  |  |  |
| Igai 2022 | - | - | - | CAT cough: 1.75 (0.83) | - | There was a significant difference in the SGRQ-I symptom domain but not in the other domains or the CAT cough score after the intervention |
|  |  |  |  | SGRQ-I: 54.19 (17.97) |  |  |
|  |  |  |  | HADS-A: 13.25 (2.05) HADS-D: 14.25 (3.82) |  |  |
| Bacci 2018 | Phase 1 | 59.3 (9.5) | 83% patients reported cough | - | - | 95% of patients endorsed cough as a symptom |
|  | Phase 2 | 70.2 (13.21) | Urge to Cough: 1.7 (0.8)  Discomfort due to Cough: 1.2 (1.0)  Intensity of Cough: 1.2 (0.9) | E-RS: IPF Domain Scores  Breathlessness: 6.3 (3.6)  Cough: 1.7 (0.8)  Chest: 3.0 (2.1)  Sputum: 2.1 (1.5) |  | The findings indicated that RS-Breathlessness and RS-Chest were most sensitive to disease severity, then RS-Cough and RS-Sputum |
| Gries 2013 | - | 87.2 (30.7) |  | CASA-Q  Symptom: 46.8 (19.2)  Impact: 57.1 (22.3) | Burden slightly worse than in COPD or chronic bronchitis | All the cough items in CASA-Q were generally perceived as highly relevant |
|  |  |  |  | Self-reported overall health  Very good/Good: 10 (55.5%)  Fair/Poor: 8 (44.5%) |  |  |
| Qualitative studies | |  |  |  | **Interview details** |  |
| Lindell 2017 | IPF and their family caregivers |  | - | - | Participants responded to the FG guide, developed following a review of the literature | Cough was the most frequently mentioned symptom, bothersome to both the patient and the caregiver. |
| Belkin 2014 | Informal caregivers defined as IPF patients loved ones | 66 (16) | - | - | FGs were facilitated by a semi structured topic guide designed in part to probe insights generated from the study team’s previous work | ICs described shortness of breath and cough as patients' main symptoms of IPF and most troubling. . These symptoms led to significant limitations in patients’ physical activity and led to frustration and resentment for the IC. |
| Duck 2012 | IPF and informal caregivers | 68 (range 44–104) | - | - | Semi-structured interviews were undertaken, audio-recorded and transcribed, and field notes made alongside interviews | Cough was often described as a presenting symptom with varying severity and in some patients a cause of incontinence |
| Overgaard 2016 | IPF patients and family caregivers | 83.1 (range 31–120) | CAT cough | CAT score: 20.1 (range 5-40) | Two dyadic pilot interviews were conducted with patients and family caregivers. | Some patients could continuously and caregivers were perpetually vigilant to coughing attacks that could be fatal |

Mean (SD) or median (range or IQR)

*Reported on alternative 0–10 cm scale

** Reported on alternative 100 point scale

ATAQ-IPF, A Tool to Assess Quality of Life in Idiopathic Pulmonary Fibrosis; BL, baseline; CAT, COPD Assessment Test; CI, confidence interval; CIC, chronic idiopathic cough; COPD, chronic obstructive pulmonary disease; CPFE, combined pulmonary fibrosis and emphysema; CQLQ, Cough Quality of Life Questionnaire; CSS, cough severity score; EQ-5D, EuroQol EQ-5D; FACIT, Functional Assessment of Chronic Illness Therapy; FG, focus group; FVC, forced vital capacity; HADS-A, Hospital Anxiety and Depression Scale Anxiety score; HADS-D, Hospital Anxiety and Depression Scale Depression score; HR, hazard ratio; HRQoL, health-related quality of life; ILD, interstitial lung disease; IPF, idiopathic pulmonary fibrosis; IQR, interquartile range; KBILD, King’s Brief Interstitial Lung Disease; LCM, Leicester Cough Monitor; LCQ, Leicester Cough Questionnaire; MCID, minimal clinically important difference; MCS, Mental Component score; NA, not applicable; NR, not reported; Physical Component score; QoL, quality of life; RCT, randomised controlled trial; SF-36, Short Form 36; SGRQ, St. George’s Respiratory Questionnaire; SGRQ-I, St. George’s Respiratory Questionnaire version for Idiopathic Pulmonary Fibrosis; VAS, visual analogue scale.

Studies in Mixed ILDs

## Table 3.1 Mixed ILD Study Designs

| Author year | Cough type | ILD Type | Location | Study type | | Trial registration | | Study duration | Setting | | |
| --- | --- | --- | --- | --- | --- | --- | --- | --- | --- | --- | --- |
| Interventional trials | |  |  |  | |  | |  |  | | |
| Bassi 2021 | Broader includes | ILD | Italy | Open label RCT | | NCT02929966 | | 18 months | Single centre | | |
| Sato 2021 | Chronic cough | CTD-ILD, IIP | Japan | Pre-post intervention study | | - | | 3 years | Multicentre | | |
| Observational studies | | | | | | | | | | | |
| Lan 2021 | Chronic cough | ILD | Australia | Cross-sectional study | | - | | 1 year | Single centre | | |
| Cheng 2017 | Chronic cough | IPF, Chronic HP, SSc-ILD | Canada | Cohort study | | - | | 5 years | Single centre | | |
| Minuk 2023 | Broader includes | ILD | Canada | Cohort study | - | | 3 years | | | Single centre |  |
| Sato 2019 | Minority cough | CHP, IIP, CTD-ILD | Japan | Cross-sectional study | | - | | 3 years | Multicentre | | |
| Veit 2023 | Majority cough | Non-IPF ILD, IPF | Germany | Prospective cohort | | - | | 6 months | Single centre | | |
| Yuan 2020 | Broader includes | CTD-ILD, IIP | China | Cross sectional, longitudinal and prospective study | | - | | 12 month | Single centre | | |
| Validation studies | | | | | | | | | | | |
| Nagata 2012 | Broader includes | ILD (exl IPF) | Japan | Validation study | | - | | NR | Single centre | | |
| Pan 2019 | Broader includes | Non-IPF ILD, IPF | China | Validation study | | - | | 2 weeks | Single centre | | |
| Yates 2018 | Broader includes | ILD | United Kingdom | Validation study | | - | | 3–6 months | Multicentre | | |
| Kirsten 2022 | Broader includes | IPF, NSIP | Germany | Validation study | | - | | 6 months | Multicentre | | |
| Mixed methods study | | | | | | | | | | | |
| Paixão 2023 | Broader includes | ILD, IPF | Portugal | Mixed methods | | NCT04224233 | | 12 months | Single centre | | |
| Qualitative studies | | |  |  | |  | |  |  | | |
| Mann 2023 | Chronic cough | HP, IPF, CTD-ILD | Australia | Qualitative (semi-structured telephone interviews) | | - | | - | Single centre | | |
| Boland 2016 | Broader includes | ILD patients, current caregivers, and clinicians | United Kingdom | Validation study | | - | | 7 months | Multicentre | | |
| Swigris 2021 | Majority cough | PF-ILD | United States and Germany | Qualitative study | | Pro00023847 | | NR | Multicentre | | |
| Bajwah 2013 | Minority cough | PF-ILD patients and informal caregivers of decedents or relatives with PIF-ILD | United Kingdom | Qualitative study | | - | | 4 months | Multicentre | | |
| Economic Studies | | | | | | | | | | | |
| Algamdi 2019 | Minority cough | Fibrotic ILD (excluding systemic diseases) | Canada | Cross sectional study | | CARE-PF | | - | Multicentre | | |

CHP, chronic hypersensitivity pneumonitis; CTD-ILD, connective tissue disease-associated ILD, HP, hypersensitivity pneumonitis; IIP, idiopathic interstitial pneumonia; ILD, interstitial lung disease; IPF, idiopathic pulmonary fibrosis; NR, not reported; NSIP, nonspecific interstitial pneumonia; PF-ILD, progressive fibrosing ILD; RCT, randomised controlled trial; SSc-ILD, systemic sclerosis-associated ILD.

## Table 3.2 Mixed ILD Patient Characteristics

| Author year | ILD type | N | Age, mean (SD)* | Female (%) | Race (%) | Comorbidities | Treatment | Disease duration, mean (SD)* |
| --- | --- | --- | --- | --- | --- | --- | --- | --- |
| Interventional trials | |  |  |  |  |  |  |  |
| Bassi 2021 | Fibrosing ILD | 50 | 75.9 (7.8) | 24% | NR | Myocardial infarction: 12 (24%), GERD 23 (46%), Pulmonary hypertension: 9 (18%) | Multidisciplinary palliative care approach | NR |
| Sato 2021 | CTD-ILD, IIP | 11 | 75.0 (64.0-77.5) | 64% | NR | NR | Chest band | NR |
| Observational studies | | | | | |  |  |  |
| Lan 2021 | ILD with cough | 118 | 69 (59-76) | 46% | White: 83%, Asian 8%, Aboriginal and Torres Strait Islander 7%, African 2% | Potential causes of cough: GORD 72 (61%), ACE-inhibitor 20 (17%), Asthma 15 (13%), UACS 15 (13%) Infection 11 (9%) | For Cough: Anti-reflux medications 69%, Inhalers with bronchodilators 45%, Cough suppressants 25%, Immune therapies for lung disease 25%, Over-the-counter medications 17%, Nasal spray or flush 13%, Antibiotics 12%, Antifibrotics 12%, Low dose azithromycin 7% | Duration of cough 6.9 (9.5) years |
|  | ILD with no cough | 46 | 69 (62-76) | 50% | White: 72%, Asian: 13%, Aboriginal and Torres Strait Islander: 13%, African: 2% | Potential causes of cough: 20 (43%), ACE-inhibitor 7 (15%) Asthma 5 (11%) UACS 3 (7%) Infection 1 (2%) | NR | NR |
| Cheng 2017 | IPF | 77 | 68.7 (7.8) | 40.3% | NR | ACE inhibitor, GERD, Chronic sinus disease, Chronic heart disease, Non-ILD chronic lung disease | NR | NR |
|  | Chronic HP | 32 | 63.8 (8.6) | 65.6% |  |  |  |  |
|  | SSc-ILD | 67 | 59.4 (12.3) | 71.6% |  |  |  |  |
| Minuk 2023 | ILD | 102 | 62 (8) | 51% | NR | Lung transplant patients | NR | NR |
|  | COPD | 24 | 57 (7) | 62% | NR | Lung transplant patients | NR | NR |
| Sato 2019 | CHP | 10 | 71.3 (7.5) | 30% | NR | GERD and dyspnoea | Glucocorticoid: 40%  Antifibrotic agent: 30%  Proton pump inhibitor: 40% | NR |
|  | CTD-ILD | 49 | 66.5 (11.4) | 63% |  |  | Glucocorticoid: 61%  Antifibrotic agent: 0%  Proton pump inhibitor: 53% |  |
|  | IIPs (incl IPF | 70 | 69.4 (8.9) | 20% |  |  | Glucocorticoid: 13%  Antifibrotic agent: 30%  Proton pump inhibitor: 30% |  |
| Veit 2023 | Non IPF-ILD | 22 | 59.8 (12.1) | 54.5% | NR | Arterial hypertension: 10 (45.5%)  Diabetes mellitus: 2 (9.1%)  Hyperlipoproteinemia: 1 (4.5%)  Coronary artery disease: 7 (31.8%)  History of revascularization: 4 (18.2%)  Osteoporosis: 2 (9.1%)  Obstructive sleep apnea syndrome: 2 (9.1%)  GERD: 5 (22.7%)  Atrial fibrillation: 1 (4.5%)  Pulmonary hypertension: 6 (27.3%) | NR | NR |
|  | IPF | 13 | 65.3 (7.2) | 23.1% | NR | Arterial hypertension: 6 (46.2%)  Diabetes mellitus: 2 (15.4%)  Hyperlipoproteinemia: 2 (15.4%)  Coronary artery disease: 1 (7.7%)  History of revascularization: 1 (7.7%)  Osteoporosis: 1 (7.7%) Obstructive sleep apnea syndrome: 2 (15.4%)  GERD: 3 (23.1%)  Atrial fibrillation: 0 (0) Pulmonary hypertension: 3 (23.1%) | NR | NR |
| Yuan 2020 | IIP | 139 | 64.3 (11) | 38% | Han: 93.5%  Other 6.5% | Asthma, pulmonary hypertension, COPD/ emphysema, lung cancer, pulmonary embolism, GERD, cardiovascular disease and metabolic diseases | Corticosteroids or/and immunosuppressants 17.3%,  Antifibrotic drugs 3.0%, No intervention 71.2%  Others 8.6% | 20.7 (26.9) months |
|  | CTD-ILD | 30 | 61.1 (10.3) | 80% | Han: 86.7%  Other 13.3% |  | Corticosteroids or/and immunosuppressants 33.3%,  Antifibrotic drugs 3.3%, No intervention 46.7%  Others 16.7% | 26.3 (37.3) months |
| Validation studies | | | | | | | | |
| Nagata 2012 | ILD (exl IPF) | 55 | 68.7 (8.3) | 58% | NR | Dyspnoea | NR | NR |
| Kirsten 2022 | IPF and NSIP | 200 | 71 (50-90) | 18% | NR | 36% had comorbidities including diabetes, arthrosis, CAD, Hypertension, Asthma etc. | NR | NR |
| Pan 2019 | ILD total  IPF  Non-IPF ILD | 92  20  72 | 58.25 (10.58) | 30% | Chinese subjects | Nearly 40% of the participants had moderate to severe dyspnoea | NR | 80% had been diagnosed less than 3 years ago |
| Yates 2018 | ILD | 95 | 66 (14) | 41% | White: 97% | Dyspnoea and fatigue | NR | NR |
| Mixed methods study | | | | | | | | |
| Paixão 2023 | IPF and ILD | 10 | 77 (3) | 50% | NR | NR | Antifibrotic (n=4), Bronchodilators (n=5), Oral corticosteroids (n=4), Immunosuppressants (n=6) | NR |
| Qualitative studies | |  |  |  |  |  |  |  |
| Mann 2023 | HP, IPF, CTD-ILD | 16 | 39 to 87 (mean/median not reported) | 50% | NR | Obstructive sleep apnoea (n=4)  Gastro-oesophageal reflux disease (n=3)  Allergic rhinitis (n=1) | NR | NR |
| Boland 2016 | ILD patients, current caregivers, and clinicians | 15 | 70 (55–89) | 36% | White: 100% |  | NR | 3.3 (0.1-6.8) years |
| Swigris 2021 | PF-ILD | 20 | 70.1 (9.5) | 50% | NR | History of unstable or deteriorating cardiac disease, COPD, Sleep disorders, GORD, Articular joint involvement | NR | 4.3 (3.1) years |
| Bajwah 2013 | PF-ILD patients and informal caregivers | 12 | 41 to 81 | 50% | White: 66.7%, 1 Black, Asian, Cypriot and S. American | COPD, osteoporosis, rheumatological  disease | NR | NR |
| Economic study | | |  |  |  |  |  |  |
| Algamdi 2019 | fibrotic ILD including IPF, NSIP, CHP | 650 | 69 (10) | 39% | NR | Dyspnoea | ILD pharmacotherapy 46% | 1.5 (0.3-3.9) years |

*Mean (SD) unless presented as median (IQR)

ACE, angiotensin-converting enzyme; CAD, coronary artery disease; CHP, chronic hypersensitivity pneumonitis; COPD, chronic obstructive pulmonary disease; CTD-ILD, connective tissue disease-associated ILD, GERD/GORD, gastroesophageal reflux disease; HP, hypersensitivity pneumonitis; IIP, idiopathic interstitial pneumonia; ILD, interstitial lung disease; IPF, idiopathic pulmonary fibrosis; NR, not reported; NSIP, nonspecific interstitial pneumonia; PF-ILD, progressive fibrosing ILD; RA, rheumatoid arthritis; RCT, randomised controlled trial; SSc-ILD, systemic sclerosis-associated ILD; UACS, upper-airway cough syndrome.

## Table 3.3 Mixed ILD Patient Outcomes

| Author year | ILD type | Disease severity (FVC % pred.) | Cough severity measures | HRQoL/impact measures | Group comparisons | Burden of cough |
| --- | --- | --- | --- | --- | --- | --- |
| Interventional trials | |  |  |  |  |  |
| Bassi 2021 | Fibrosing ILD | 69.6 (2.15) | VAS: 52.3 (32.8) | MRQr: 11.6 (6.0) | VAS cough worsened both in intervention and usual care group (p-value group effect: 0.88) | Depression (CES-D) and dyspnoea (MRQr) were improved by the intervention, but cough was not. |
|  |  |  |  | CES-D: 13.8 (8.1) |  |  |
| Sato 2021 | CTD-ILD, IIP | 77.8 (63.9–88.1) | VAS* | LCQ (acute): 13.4 (IQR,11.0–14.9) | - | Correlation not presented |
| Observational studies | |  |  |  |  |  |
| Cheng 2017 | IPF | 73.2 (18.1) | VAS: no baseline | SGRQ: No baseline | Cough was most prevalent in IPF and most productive in CHP | Cough severity was an independent predictor of total SGRQ after adjustments in SSc-ILD and IPF but not CHP |
|  | CHP | 67.0 (19.8) |  |  |  |  |
|  | SSc-ILD | 74.6 (22.0) |  |  |  |  |
| Lan 2021 | ILD with cough | 74.6 (18.7) | VAS: 41.8 (25.9) | LCQ: 14.9 (4.3)  Physical: 5.1 (1.5) Physiological: 5.3 (1.7) Social: 4.5 (1.5) | Prevalence of cough was highest in IPF, NSIP and sarcoidosis patients (>70%) | Cough ranked as worse ILD symptom in over a third of patients |
|  | ILD with no cough | 87.0 (15.9) |  |  |  |  |
| Sato 2019 | IIPs (incl IPF) | 85.2 (74.2–97.3) | VAS: 31 (17–55) | LCQ: 19.3 (IQR:17.5–20.4) | Patients with the IIPs had the greatest intensity of cough but not frequency of cough | Patients in whom cough frequency was predominant had a greater impairment of health status relative to other patients.  Significant correlation between total LCQ scores and intensity and frequency of cough were − 0.675 and − 0.762, respectively |
|  | CTD-ILD | 93.6 (80.2–106.1) | VAS: 24 (8-46) | LCQ: 18.7 (IQR:15.4–20.5) |  |  |
|  | CHP | 73.6 (68.8–93.3) | VAS: 18 (6-20) | LCQ: 19.6 (IQR:18.3–20.6) |  |  |
| Veit 2023 | Non IPF-ILD | 63.4 (23.5) | VAS: 2.5 (2.4)* | SGRQ: 48.9 (20) | Patients with IPF not only had a higher burden of cough at the beginning of the study, but also experienced a greater increase in cough over time than those with non-IPF ILD.  Patients with IPF had significantly more limitations in terms of KBILD values compared to those with non-IPF ILD (p = 0.022). SGRQ did not show significant differences between IPF and non-IPF ILD (p = 0.193). | For KBILD, but not SGRQ, there was a significant inverse correlation with VAS cough |
|  |  |  |  | KBILD: 53.1 (12.1) |  |  |
|  | IPF | 68.5 (18.7) | VAS: 4.6 (2.7)* | SGRQ: 51.1 (9.8) |  |  |
|  |  |  |  | KBILD: 48.2 (2.6) |  |  |
| Minuk 2023 | ILD | 46 (12) |  | ESAS cough score: 7 (IQR 4–9) | Cough was worse in patients with ILD at baseline and they had lower drowsiness scores compared to COPD patients. |  |
|  | COPD | NR |  | ESAS cough score: 4 (IQR 1–7) |  |  |
| Yuan 2020 | IIP | 86.9 (22.2) | Chinese LCQ | LCQ: 16.7 (3.7)  Physical: 5.4 (1.3) Physiological: 5.5 (1.3) Social: 5.8 (1.3) | Average cough scores were comparable between IIP and CTD-ILD, HRQoL was lower in CTD-ILD | Cough impact correlates with quality of life at baseline and over time |
|  |  |  |  | SGRQ: 32.9 (19.1) |  |  |
|  |  |  |  | HADS-A: 5.0 (3.0–7.0)  HADS-D: 5.0 (1.0–7.0) |  |  |
|  |  |  |  | SF-36  Physical: 37.2 (12.0)  Mental: 48.3 (11.6) |  |  |
|  | CTD-ILD | 74.4 (19.1) | Chinese LCQ | LCQ: 16.3 (3.7)  Physical: 5.3 (1.3) Physiological: 5.4 (1.3) Social: 5.7 (1.3) |  |  |
|  |  |  |  | SGRQ: 43.3 (20.6) |  |  |
|  |  |  |  | HADS-A: 6.0 (3.0–9.0)  HADS-D: 5.5 (2.7–9.2) |  |  |
|  |  |  |  | SF-36  Physical: 31.1 (14.2)  Mental: 45.6 (11.1) |  |  |
| Validation studies | |  |  |  |  |  |
| Nagata 2012 | ILD (exl IPF) | 72.7 (17.3) |  | LCQ: 97.5 (39–133) | - | Cough strongly contributes to quality of life |
|  |  |  |  | SGRQ: 43.2 (0.0–83.9) |  |  |
|  |  |  |  | CAT: 13 (1–33) |  |  |
|  |  |  |  | HADS-A: 4 (0–15)  HADS-D: 4 (0–13) |  |  |
| Pan 2019 | IPF | NR |  | SGRQ:78.65 (10.84) | Cough domains were significantly worse in IPF than non-IPF ILD (p<0.001) | Many aspects of health-related quality of life were impaired in IPF according to the cATAQ-IPF score |
|  |  |  |  | cATAQ-IPF total: 287.90 (22.56)  cough: 24.70 (4.66) |  |  |
|  | Non IPF ILD | NR |  | SGRQ: 57.47 (21.81) |  |  |
|  |  |  |  | cATAQ-IPF total: 250.74 (47.39)  cough: 17.58 (7.80) |  |  |
| Yates 2018 | IPF | NR | VAS: 38 (20) | NR | No significant difference in cough severity at baseline or interval change between IPF and non IPF subgroups. | VAS Cough did not correlate with KBILD |
|  | Non-IPF ILD | NR | VAS: 44 (29) | NR |  |  |
|  | Total initial cohort | 82.5 (18.8) | VAS: 43 (26) | KBILD: 62.6 (21.4) |  |  |
|  | Total validation cohort | 88.9 (20.1) | VAS: 41 (30) | KBILD: 62.5 (22.7) |  |  |
| Kirsten 2022 | IPF and NSIP | NR |  | SGRQ: 38.8 | - | Cough scale included in the quality-of-life tool |
|  |  |  |  | QPF-scale total: 97.11  QPF-scale cough: 4.23 |  |  |
| Mixed methods study | | | | | | |
| Paixão 2023 | ILD and IPF | 77.1 (4.4) |  | CASA-Q cough symptoms: 83.3 (75–100)  CASA-Q cough impact: 100 (78.1–100) | Cough symptoms improved after 12 weeks of intervention | Correlation with quality of life not presented |
|  |  |  |  | SGRQ total: 48.6 (19.4) |  |  |
|  |  |  |  | CAT: 14.9 (8.4) |  |  |
|  |  |  |  | HADS-A: 5.3 (5)  HADS-D: 7.1 (4.5) |  |  |
| Qualitative studies | |  |  |  | **Interview details** |  |
| Mann 2023 | HP, IPF, CTD-ILD | FVC: 53-107 (mean/median NR) | VAS: 25-99 (mean/median NR) | Major themes identified: triggers for cough, cough impacts, and educational needs and management  Minor themes identified: cough interpretation and impacts of the COVID-19 pandemic. | Individual semi-structured telephone interviews were conducted by a researcher with training in qualitative study conduct using a topic guide conceived from the investigator team’s clinical experience and literature review. | Chronic cough significantly restricted public engagement and social participation, with many patients feeling self-conscious and having the fear of uncontrollable coughing fit in public.  Cough was described as a reminder of their own disease; however it was generally accepted as a symptom of ILD. |
| Boland 2016 | ILD patients, current caregivers, and clinicians |  | - | NAT:PD-ILD | Two patient groups FG; one carer group FG; one clinician group FG and a single caregiver interview | Cough bothersome to patients and caregivers |
| Swigris 2021 | PF-ILD | 67.0 (13.6) | Cough symptoms in 24hrs | L-PF questionnaire with a range of cough sufferers | Patients interviews regarding the L-PF | Cough was reported by the majority of patients (19 out of 20 patients). Nine patients described their cough as occurring daily. When asked about the severity of their cough, one participant described it as severe, one as affecting activities, three as not severe but nagging/annoying, and two as “usually not bad” |
| Bajwah 2013 | PF-ILD patients, informal caregivers, relatives |  | 5/12 reported cough |  | The interviews followed a topic guide following a review of the literature and a review of deceased patients’ notes. | Patient participants highlighted that cough was a common and highly irritating problem |
| Economic study | |  |  |  | **Group comparisons** |  |
| Algamdi 2019 | Fibrotic ILD employed | 74.8 (20) | VAS: no baseline | Estimated annual costs of productivity loss:  11,610 CAD per patient  Hours lost, mean (SE): 7.8 (0.9) | The costs of productivity loss were comparable between employed male and female patients and between IPF and non-IPF patients ($11,737 vs $11,535) | Cough was an independent predictor of workplace productivity loss |
|  | Fibrotic ILD unemployed | 73.3 (20.5) |  |  |  |  |

Mean (SD) or Median (range or IQR)

*Reported on alternative 0–10 cm scale

CAN, Canadian dollar; CAT, COPD Assessment Test; cATAQ-IPF, Chinese version of the A Tool To Assess Quality of Life in Idiopathic Pulmonary Fibrosis; CES-D, Center for Epidemiologic Studies Depression Scale; CHP, chronic hypersensitivity pneumonitis; CTD-ILD, connective tissue disease-associated interstitial lung disease, FVC, forced vital capacity; HADS-A, Hospital Anxiety and Depression Scale Anxiety score; HADS-D, Hospital Anxiety and Depression Scale Depression score; HRQoL, health-related quality of life; IIP, idiopathic interstitial pneumonia; ILD, interstitial lung disease; IPF, idiopathic pulmonary fibrosis; IQR, interquartile range; KBILD, King’s Brief Interstitial Lung Disease; LCM Leicester Cough Monitor; LCQ, Leicester Cough Questionnaire; MRQr, Maugeri Respiratory Questionnaire; NR, not reported; NSIP, non-specific interstitial pneumonia, PF-ILD, progressive fibrosing interstitial lung disease; QPF, Quality of life in patients with idiopathic pulmonary fibrosis tool; RA, rheumatoid arthritis; RCT, randomised controlled trial; SE, standard error; SGRQ, St. George’s Respiratory Questionnaire; SSc, systemic sclerosis; VAS, visual analogue scale.

Studies in CTD-ILDs

## Table 4.1 CTD-ILD Study Designs

| Author year | ILD Type | Cough type | Location | Study type | Trial registration | Study duration | Setting |
| --- | --- | --- | --- | --- | --- | --- | --- |
| Interventional trials | |  |  |  |  |  |  |
| Theodore 2012 | SSc-ILD | Majority cough | United States | RCT | NCT000004563/ Scleroderma Lung Study | 12 months | Multicentre |
| Tashkin 2016/ Tashkin 2017/ Volkmann 2020 | SSc-ILD | Majority cough | United States | *Post hoc* analysis (RCT) | NCT00883129/ Scleroderma Lung Study II | 2 years | Multicentre |
| Volkmann 2022 | SSc-ILD | Majority cough | United Kingdom, United States, 18 European countries and 12 other countries | *Post hoc* analysis (RCT) | NCT02597933/ SENSCIS | 12 months | Multicentre |
| Observational study | |  |  |  |  |  |  |
| Topcu 2021 | RA-ILD, CTD-ILD (incl SSc-ILD) | Broader includes | Turkey | Cross sectional study | - | 1 month | Multicentre |
| Validation study | |  |  |  |  |  |  |
| Fisher 2019 | SSc-ILD | Minority cough | United States | Validation study | - | 18 months | Single centre |
| Mixed methods study | |  |  |  |  |  |  |
| Mittoo 2015 | CTD-ILD | Broader includes | United States | Mixed method design | - | - | Multicentre |

CTD-ILD, connective tissue disease-associated interstitial lung disease; SSc-ILD, systemic sclerosis interstitial lung disease; RA-ILD, rheumatoid arthritis-associated interstitial lung disease; RCT, randomised controlled trial.

## Table 4.2 CTD-ILD Patient Characteristics

| Author year | ILD type | N | Age (mean (SD))* | | | Female (%) | Race (%) | | Comorbidities | | Treatment | | Disease duration (mean (SD))* |
| --- | --- | --- | --- | --- | --- | --- | --- | --- | --- | --- | --- | --- | --- |
| Interventional trials | | | |  |  | | |  | |  | |  | |
| Tashkin 2016/SLS II | SSc-ILD | 142 | 52.3 (9.7) | | 74% | | NR | | Dyspnoea | | Cyclophosphamide, Mycophenolate | | Non-Raynauds:  2.6 (range 0.3–7.1) years |
| Tashkin 2017/SLS II | SSc-ILD with frequent cough | 87 | 52.9 (9.23) | | NR | | NR | | Diffuse cutaneous disease: 56%  GERD: 77% | | Mycophenolate Mofetil and oral cyclophosphamide | | Non-Raynauds:  2.5 (1.7) years |
|  | SSc-ILD without frequent cough | 54 | 51 (10.49) | | NR | | NR | | Diffuse cutaneous disease: 61%  GERD: 59% | |  |  | Non-Raynauds:  2.7 (1.9) years |
| Volkmann 2020/SLS II | SSc-ILD | 73 | 52.0 (9.8) | | 78% | | NR | | Dyspnoea | | Cyclophosphamide | | Non-Raynauds:  2.5 (1.8) years |
|  |  | 69 | 52.6 (9.7) | | 70% | | NR | | Dyspnoea | | Mycophenolate | | Non-Raynauds:  2.6 (1.7) years |
| Theodore 2012/SLS | SSc-ILD | 156 | 47.9 (1.0) | | 70% | | NR | | GERD, dyspnoea | | oral cyclophosphamide | | SSc: 3.2 (0.2) years |
| Volkmann 2022 | SSc-ILD with cough | 229 | 54.8 (11.8) | | 75% | | NR | | Asthma, GERD, dyspnoea | | Nintedanib | | Non-Raynauds:  3.5 (1.6) years |
|  |  | 232 | 53.5 (12.8) | | 72% | |  |  |  |  | Placebo | | Non-Raynauds:  3.5 (1.8) years |
|  | SSc-ILD without cough | 58 | 53.8 (11.9) | | 85% | |  |  |  |  | Nintedanib | | Non-Raynauds:  3.5 (1.6) years |
|  |  | 56 | 52.6 (11.8) | | 82% | |  |  |  |  | Placebo | | Non-Raynauds:  3.5 (1.8) years |
| Observational study | | |  | |  | |  | |  | |  | |  |
| Topcu 2021 | RA-ILD, CTD-ILD (incl SSc-ILD) | 39 | 60 (7) | | 71% | | NR | | Dyspnoea, diabetes, hypertension, hyperlipidaemia, CAD, COPD, bronchiectasis | | NR | | 10 (IQR = 10) years |
| Validation study | | | | | | | | | | | | | |
| Fisher 2019 | SSc-ILD | 73 | 51.9 (11.8) | | 81% | | White: 86.3%,  Black 8.2%,  Other 5.5% | | Dyspnoea | | NA | | Non-Raynauds:  7.9 (8.3) years  ILD: 4.7 (7.4) years |
| Mixed methods study | | | | | | | | | |  | |  | |
| Mittoo 2015 | CTD-ILD | 45 | Mean age across focus groups ranged from 52.4 to 64.3 | | 73% | | Caucasian: 67%  African American: 24%  Asian: 4%  African/Caribbean: 2%  Hispanic: 2%  Other: 2% | | NR | | NR | | NR |

CAD, coronary artery disease; COPD, chronic obstructive pulmonary disease; CTD-ILD, connective tissue disease-associated interstitial lung disease; GERD, gastroesophageal reflux disease; NR, not reported; SSc-ILD, systemic sclerosis interstitial lung disease; RA-ILD, rheumatoid arthritis-associated interstitial lung disease; RCT, randomised controlled trial.

## Table 4.3 CTD-ILD Patient Outcomes

| Author year | ILD type | Disease severity (FVC % pred.) | Cough severity measures | HRQoL/impact measures | Comparison groups | Burden of cough |
| --- | --- | --- | --- | --- | --- | --- |
| Interventional trials | | | | | | |
| Theodore 2012/SLS | SSc-ILD with cough | 65.85 (11.16) | Cough index (scale of severity and frequency) | SF 36  Mental: 47.88 (10.82)  Physical: 29.65 (8.76) | Those with cough were analysed by severity and frequency of cough – mild (62%) moderate (32%) and severe (5%) and infrequent (62%), intermittent (33%) and persistent (6%) | Prescence of cough was significantly correlated with lower physical QoL but severity of cough did not correlate significantly with any baseline variable |
|  |  |  |  | HAQ-DI: 0.98 (0.68) |  |  |
|  | SSc-ILD without cough | 69 (10.5) | - | SF 36  Mental: 48.62 (11.7)  Physical: 38.47 (10.4) |  |  |
|  |  |  |  | HAQ-DI: 0.74 (0.63) |  |  |
| Volkmann 2022 | SSc-ILD with cough | 71.5 (16.1) |  | Nintedanib group  SGRQ: 43.9 (18.8) | HRQoL scores worse in patients with cough | Cough correlates with fibrosis extent at baseline |
|  |  |  |  | Placebo group  SGRQ: 42.3 (20.3) |  |  |
|  | SSc-ILD without cough | 76.7 (18.3) | - | Nintedanib group  SGRQ: 28.0 (20.7) |  |  |
|  |  |  |  | Placebo group  SGRQ: 27.1 (19.4) |  |  |
| Volkmann 2020/SLS II | SSc-ILD treated with cyclophosphamide | 66.5 (9.9) |  | LCQ: 16.7 (4) | Both drugs improved PRO scores | Baseline LCQ scores all correlated with the extent of quantitative radiographic fibrosis and ILD as well as with measures of cutaneous sclerosis.  Change in LCQ scores did not correlate significantly with any objective measure of SSc-ILD disease severity |
|  |  |  |  | SGRQ: 36.8 (17.5) |  |  |
|  |  |  |  | HAQ-DI: 0.7 (0.7) |  |  |
|  |  |  |  | SF-36  Physical: 35.6 (9.8)  Mental: 49.8 (10) |  |  |
|  | SSc-ILD treated with mycophenolate | 66.5 (8.3) |  | LCQ: 16.8 (4) |  |  |
|  |  |  |  | SGRQ: 37.3 (17.4) |  |  |
|  |  |  |  | HAQ-DI: 0.7 (0.6) |  |  |
|  |  |  |  | SF-36  Physical: 36 (10)  Mental: 49.1 (7.9) |  |  |
| Tashkin 2016/SLS II | SSc-ILD | 66.5 (9.1) |  | LCQ:16.7 (4.0) | - | Cough correlates with fibrosis extent |
|  |  |  |  | HAQ-DI: 0.7 (0.7) |  |  |
|  |  |  |  | SF-36  Physical 35.8 (9.9)  Mental 49.4 (9.0) |  |  |
| Tashkin 2017/SLS II | SSc-ILD with frequent cough | 65.6 (8.8) |  | LCQ: 15.4 (3.7) | Study participants who reported FC at baseline (61.3%) reported significantly more dyspnoea, exhibited more extensive ILD on high-resolution CT, had a lower diffusing capacity for carbon monoxide, and reported more GERD symptoms than did those without FC. | Cough related quality of life significantly correlated with HRQoL both at baseline and over treatment time. |
|  |  |  |  | SF 36  Mental: 48.8 (8.6)  Physical: 35.0 (9.8) |  |  |
|  |  |  |  | HAQ-DI: 0.66 (0.57) |  |  |
|  | SSc-ILD without frequent cough | 67.8 (9.4) | - | SF 36  Mental: 50.7 (9.6) Physical: 37.2 (9.9) |  |  |
|  |  |  |  | HAQ-DI: 0.80 (0.81) |  |  |
| Observational study | | | | | | |
| Topcu 2021 | RA-ILD | 92 (28) |  | LCQ: 17.8 (5.1), | Compared to patients with CTD, patients with RA-ILD have worse HRQoL, as measured by the SGRQ and SF-36 physical functioning score; median scores of LCQ were similar. | No correlation reported but concluded PROs may not differentiate ILD cough from non-ILD cough in RA/CTD. |
|  |  |  |  | SGRQ: 79.4 (8.7), |  |  |
|  |  |  |  | SF-36: 60.9 (13.7) |  |  |
|  | CTD-ILD (incl SSc-ILD) | 91 (47) |  | LCQ: 18.5 (3.7) |  |  |
|  |  |  |  | SGRQ: 27.1 (26.5) |  |  |
|  |  |  |  | SF-36: 63.9 (21.9) |  |  |
|  | Total | 91.5 (38.5) |  | LCQ: 18.4 (4.2) |  |  |
|  |  |  |  | SGRQ: 40.4 (57.6) |  |  |
|  |  |  |  | SF-36: 63.2 (19.4) |  |  |
| Validation study | | | | | | |
| Fisher 2019 | SSc-ILD | 73.9 (15.5) |  | LCQ: 17.5 (3.1) | - | The average LCQ score indicated mild cough and the scores did not correlate with the corresponding PROMIS domains even in patients who reported cough (41% of the cohort) |
|  |  |  |  | SGRQ: 32.6 (19.0) |  |  |
|  |  |  |  | SF-36  Physical 35.9 (12.7)  Mental 46.6 (11.3) |  |  |
|  |  |  |  | PROMIS*  Physical function: 41.4 (8.1)  Social role: 45.9 (8.2)  Anxiety: 52.5 (9.6)  Depression: 51.2 (11.0)  Fatigue: 56.4 (10.4)  Pain Interference: 55.9 (11.0)  Sleep Disturbance: 52.9 (11.0)  Pain: 3.5 (2.7) |  |  |
| Mixed methods study | | | |  | **Interview details** |  |
| Mittoo 2015 | CTD-ILD | Available for 2 of 6 focus groups:  55 (45–67)  46 (23-75) | Post focus group questionnaire | WHOQOL-100 | FG interviews and subsequent quantitative self-administered questionnaire | Cough a hallmark symptom of ILD affecting social and physical QoL |

*All reported on a 0–100-point scale aside from pain, which is reported on a 0-10 scale

CT, computed tomography; CTD-ILD, connective tissue disease-associated interstitial lung disease; FC, frequent cough; FG, focus group; FVC, forced vital capacity; GERD, gastroesophageal reflux disease; HAQ-DI, Health Assessment Questionnaire Disability Index; HRQoL,health-related quality of life; IQR, interquartile range; LCQ, Leicester Cough Questionnaire; NR, not reported; PRO, patient-reported outcome; QoL, quality of life; RA-ILD, rheumatoid arthritis-associated interstitial lung disease; RCT, randomised controlled trial; SF-36, Short Form 36; SGRQ, St. George’s Respiratory Questionnaire; SLS, Scleroderma Lung Study; SSc-ILD, systemic sclerosis interstitial lung disease; WHOQOL, World Health Organization Quality of Life tool.

Studies in Sarcoidosis

## Table 5.1 Sarcoidosis Study Designs

| **Author year** | **Cough type** | | **Location** | **Study type** | **Trial registration** | **Study duration** | **Setting** |
| --- | --- | --- | --- | --- | --- | --- | --- |
| **Interventional trial** |  | |  |  |  |  |  |
| Fraser 2020 | Chronic cough | | United Kingdom | Open label single-arm trial | EudraCT 2019-000580-24 | 3 months | Single centre |
| **Observational studies** | |  | |  |  |  |  |
| Sinha 2016 | Majority cough | | United Kingdom | Cross-sectional | - | NR | Single centre |
| Judson 2017 | Minority cough | | United States | Cohort study | - | 2 years | Single centre |
| Gvozdenovic 2020 | Minority cough | | Serbia | Cohort study | - | 18 months | Single centre |

## Table 5.2 Sarcoidosis Patient Characteristics

NR, not reported.

| Author year | N | Age (mean (SD))* | Female (%) | Race (%) | Comorbidities | Treatment | Disease duration (mean (SD))* | |
| --- | --- | --- | --- | --- | --- | --- | --- | --- |
| Interventional trial | |  |  |  |  |  | | |
| Fraser 2020 | 21 | 57 (48-71) | 57% | White: 100% | NR | Azithromycin | | 3 (1 –13) years |
| Observational studies | |  |  |  |  |  | | |
| Sinha 2016 | Sarcoidosis patients: 32 | 50 (13) | 63% | Afro-Caribbean 66%,  White: 25%, South Asian 6%, Other 3% | Common comorbidities excluded | Immunosuppressant 38%,  steroid 6% | | 2 (1–4) years  Duration of cough: 96 (28–144) weeks |
|  | Healthy subjects: 40 | 49 (13) | 68% | NR | NR | NR | | NR |
| Judson 2017 | 355 | 53 (13) | 56% | White: 81%,  Black 19%,  Asian 1% | NR | Prednisone | | NR |
| Gvozdenovic 2020 | 275 | 50.13 (11.07) | 66% | NR | Dyspnoea, all else excluded | Prednisone 35.6% Methotrexate 53.1% Chloroquine 1.8% Prednisone + Methotrexate 3.3% | | 15.62 (8.56) years |

## Table 5.3 Sarcoidosis Patient Outcomes

| Author year | ILD type | Disease severity (FVC % pred.) | Cough severity measures | HRQoL/impact measures | Comparing groups | | Burden of cough |
| --- | --- | --- | --- | --- | --- | --- | --- |
| Interventional trial | | | |  | |  |  |
| Fraser 2020 | Pulmonary sarcoidosis | 91.5 (63–128) | HACC 24hr cough count: 228 (43–1950)  Coughs per hour: 10 (2–81) | LCQ: 14.63 (4.07) | Baseline cough count was significantly higher in patients with baseline cough severity VAS>40 mm compared with <40 mm | | Changes in cough counts correlated with changes in LCQ and KSQ GH but not with KSQ lung domain scores. |
|  |  |  | VAS: 38.8 (25.7)  Urge to cough VAS: 38.7 (26.2) | KSQ: 57.3 (9.1)  KSQ GH: 52.93 (18.3)  KSQ Lung: 52.0 (10.4) |  |  |  |
| Observational studies | | | |  | |  |  |
| Sinha 2016 | Pulmonary sarcoidosis with cough | NR | VAS: 53 (20–66) | LCQ: 14.8 (3.7)  Physical 4.8 (1.3) Psychological 5.0 (1.3)  Social 5.0 (1.5)  CHQ: 10 (5–14) | Cough frequency was significantly higher than healthy subjects, but less than that reported in patients with idiopathic chronic cough | | Cough (both 24h counts and VAS) was significantly associated with health status, affecting all LCQ health domains |
|  |  |  | LCM 24hr cough count: 244 (2)  Coughs per hour: 10 (2) |  |  |  |  |
|  |  |  | Cough reflex sensitivity (C_5_ μmol·L^−1^): 6.8 (3.2) |  |  |  |  |
|  | All pulmonary sarcoidosis | 83.7 (16.8) | LCM 24hr cough count: 67 (5) | CHQ: 6 (3–12) |  |  |  |
|  |  |  | Cough reflex sensitivity (C_5_ μmol·L^−1^): 13.3 (4.5) |  |  |  |  |
|  | Healthy subjects | 100.9 (29) | LCM 24hr cough count: 18 (3) | NR |  |  |  |
|  |  |  | Cough reflex sensitivity (C_5_ μmol·L^−1^): 61.5 (6.5) |  |  |  |  |
| Judson 2017 | All sarcoidosis | No baseline | VAS: 2.5 (1.5)* | LCQ: 17.5 (3.0)  Physical: 5.9 (1.3) Psychological: 5.9 (1.3)  Social: 5.3 (0.9) | Cough was significantly worse in patients with pulmonary involvement compared to those without. | | Cough severity measured by VAS correlated significantly with LCQ domains except social.  Those with VAS scores >4 had higher total LCQ scores than those with VAS scores <4 |
|  | Pulmonary sarcoidosis |  | VAS: 2.6 (2.8)** | LCQ: 16.9 (3.5)  Physical: 5.7 (1.5) Psychological: 5.9 (1.5)  Social: 5.2 (1) |  |  |  |
|  | Non pulmonary sarcoidosis |  | VAS: 1.3 (2.2)** | LCQ: 18.8 (3)  Physical: 6.3 (1.3) Psychological: 6.5 (1.2)  Social: 5.9 (1) |  |  |  |
| Gvozdenovic 2020 | Pulmonary sarcoidosis | 108.52 (17.40) | - | LCQ: 16.94 (3.68) Physical: 5.48 (1.18) Psychological: 5.64 (1.29)  Social: 5.82 (1.33) | - | | Dyspnoea was the strongest predictor of cough-specific and generic QoL and the physical domain of the LCQ was a significant predictor of QoL. |
|  |  |  |  | 15D: 0.85 (0.11) |  |  |  |

*Reported on alternative 0–10 cm scale

******Data amended due to author correspondence^[[1]](#footnote-1)^

15D; fifteen-dimensional measure of health-related quality of life; CHQ, Cough Hypersensitivity Questionnaire; FVC, forced vital capacity; GH, General Health score; HACC, The Hull Automatic Cough Counter; HRQOL, health-related quality of life; KSQ, King’s Sarcoidosis Questionnaire; LCM, Leicester Cough Monitor; LCQ, Leicester Cough Questionnaire; NR, not reported; QoL, quality of life; VAS, visual analogue scale.

1. Judson M. Correction [Personal communication]. 22.1.24. [↑](#footnote-ref-1)
